# Translating community-wide spectral library into actionable chemical knowledge: a proof of concept with monoterpene indole alkaloids

**DOI:** 10.1186/s13321-025-01009-0

**Published:** 2025-04-28

**Authors:** Sarah Szwarc, Adriano Rutz, Kyungha Lee, Yassine Mejri, Olivier Bonnet, Hazrina Hazni, Adrien Jagora, Rany B. Mbeng Obame, Jin Kyoung Noh, Elvis Otogo N’Nang, Stephenie C. Alaribe, Khalijah Awang, Guillaume Bernadat, Young Hae Choi, Vincent Courdavault, Michel Frederich, Thomas Gaslonde, Florian Huber, Toh-Seok Kam, Yun Yee Low, Erwan Poupon, Justin J. J. van der Hooft, Kyo Bin Kang, Pierre Le Pogam, Mehdi A. Beniddir

**Affiliations:** 1 Équipe, Chimie des Substances Naturelles, Université Paris-Saclay, CNRS, BioCIS, 17 avenue des Sciences, 91400 Orsay, France; 2 Institute of Molecular Systems Biology, ETH Zürich, 8093 Zurich, Switzerland; 3 College of Pharmacy and Research Institute of Pharmaceutical Sciences, Sookmyung Women’s University, Seoul, 04310 Republic of Korea; 4 Université Paris-Dauphine, PSL Research University, CNRS, LAMSADE, 75016 PARIS, France; 5 Laboratory of Pharmacognosy, Center of Interdisciplinary Research On Medicines (CIRM), University of Liège, Liège, Belgium; 6 Department of Chemistry, Faculty of Science, Universiti Malaya, 50603 Kuala Lumpur, Malaysia; 7 El Batan, Instituto de BioEconomia, Quito, 170135 Ecuador; 8 Département Science Fondamentale, Service Chimie-Biochimie, Université Des Sciences de La Santé, Owendo, Gabon; 9 Department of Pharmaceutical Chemistry, Faculty of Pharmacy, College of Medicine, University of Lagos, Idiaraba Campus, Surulere, Lagos Nigeria; 10 Natural Products Laboratory, Institute of Biology, Leiden University, Sylviusweg 72, 2333 BE Leiden, the Netherlands; 11 EA2106 Biomolécules et Biotechnologies Végétales, Université de Tours, 31 Avenue Monge, 37200 Tours, France; 12 UMR 8038 CiTCoM, Faculté de Santé, Université Paris Cité, CNRS, 75006 Paris, France; 13 Centre for Digitalisation and Digitality, Düsseldorf University of Applied Sciences, 40476 Düsseldorf, Germany; 14 Bioinformatics Group, Wageningen University & Research, 6708 PB Wageningen, the Netherlands; 15 Department of Biochemistry, University of Johannesburg, Johannesburg, 2006 South Africa

**Keywords:** Monoterpene indole alkaloids, MS/MS, Query, Scaffold, Similarity, Expert knowledge

## Abstract

With over 3000 representatives, the monoterpene indole alkaloids (MIAs) class is among the most diverse families of plant natural products. The MS/MS spectral space exploration of these complex compounds using chemoinformatic and computational mass spectrometry tools offers a valuable opportunity to extract and share chemical insights from this emblematic family of natural products (NPs). In this work, we first present a substantially updated version of the MIADB, a database now containing 422 MS/MS spectra of MIAs that has been uploaded to the GNPS library versus 172 initial entries. We then introduce an innovative workflow that leverages hundreds of fragmentation spectra to support the FAIRification, extraction and dissemination of chemical knowledge. This workflow aims at the extraction of spectral patterns matching finely defined MIA skeletons. These extracted signatures can then be queried against complex biological extract datasets using MassQL. By applying this strategy to an LC-MS/MS dataset of 75 plant extracts, our results demonstrated the efficiency of this approach in identifying the diversity of MIA skeletons present in the analyzed samples. Additionally, our work enabled the digitization of structural data for diverse MIA skeletons by converting them into machine-readable formats and thereby enhancing their dissemination for the scientific community.

**Scientific contribution** A comprehensive investigation of the monoterpene indole alkaloid chemical space, aiming to highlight skeleton-dependent fragmentation similarity trends and to generate valuable spectrometric signatures that could be used as queries.

**Graphical Abstract:**

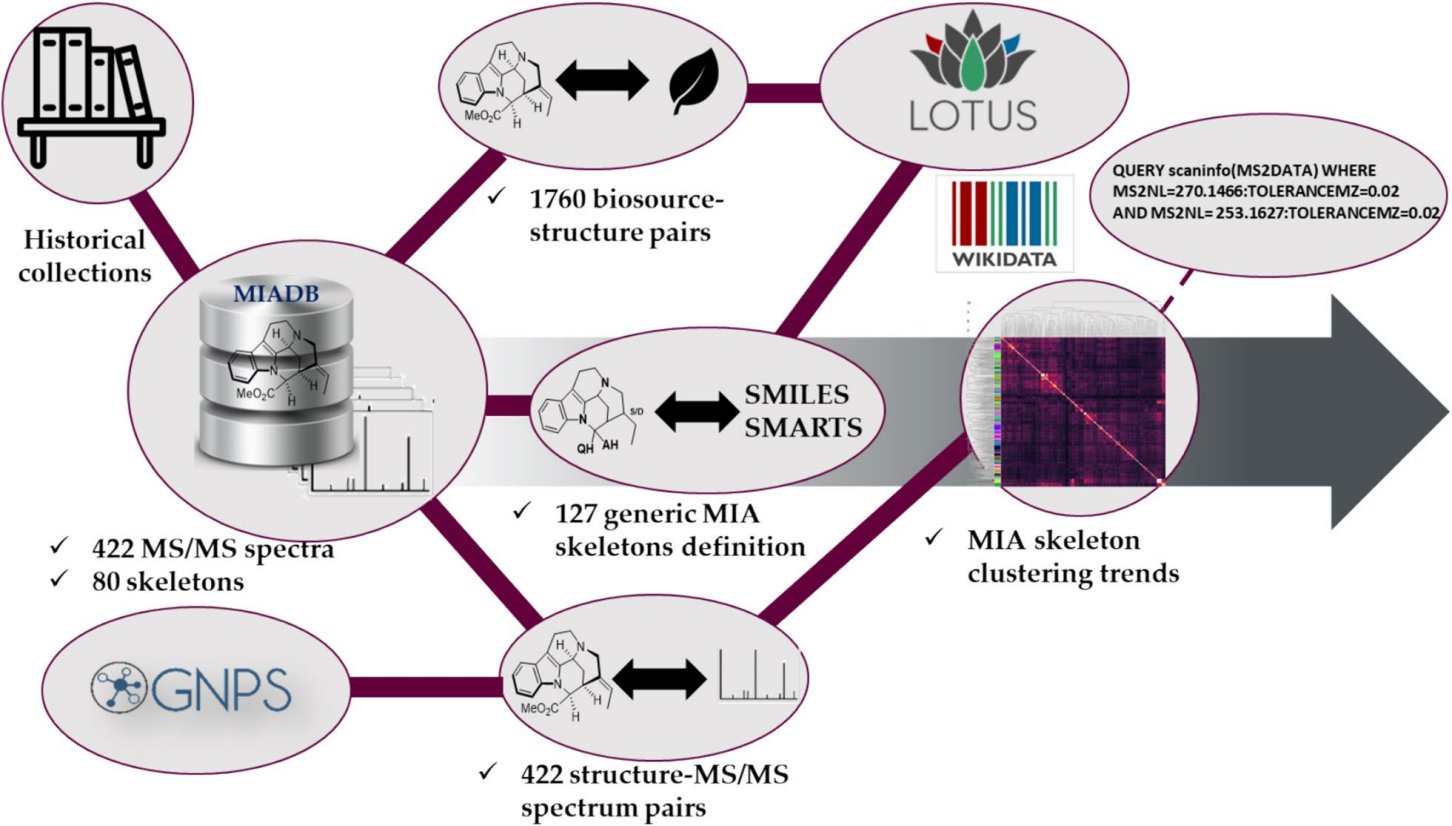

**Supplementary Information:**

The online version contains supplementary material available at 10.1186/s13321-025-01009-0.

## Introduction

**Fig. 1:**
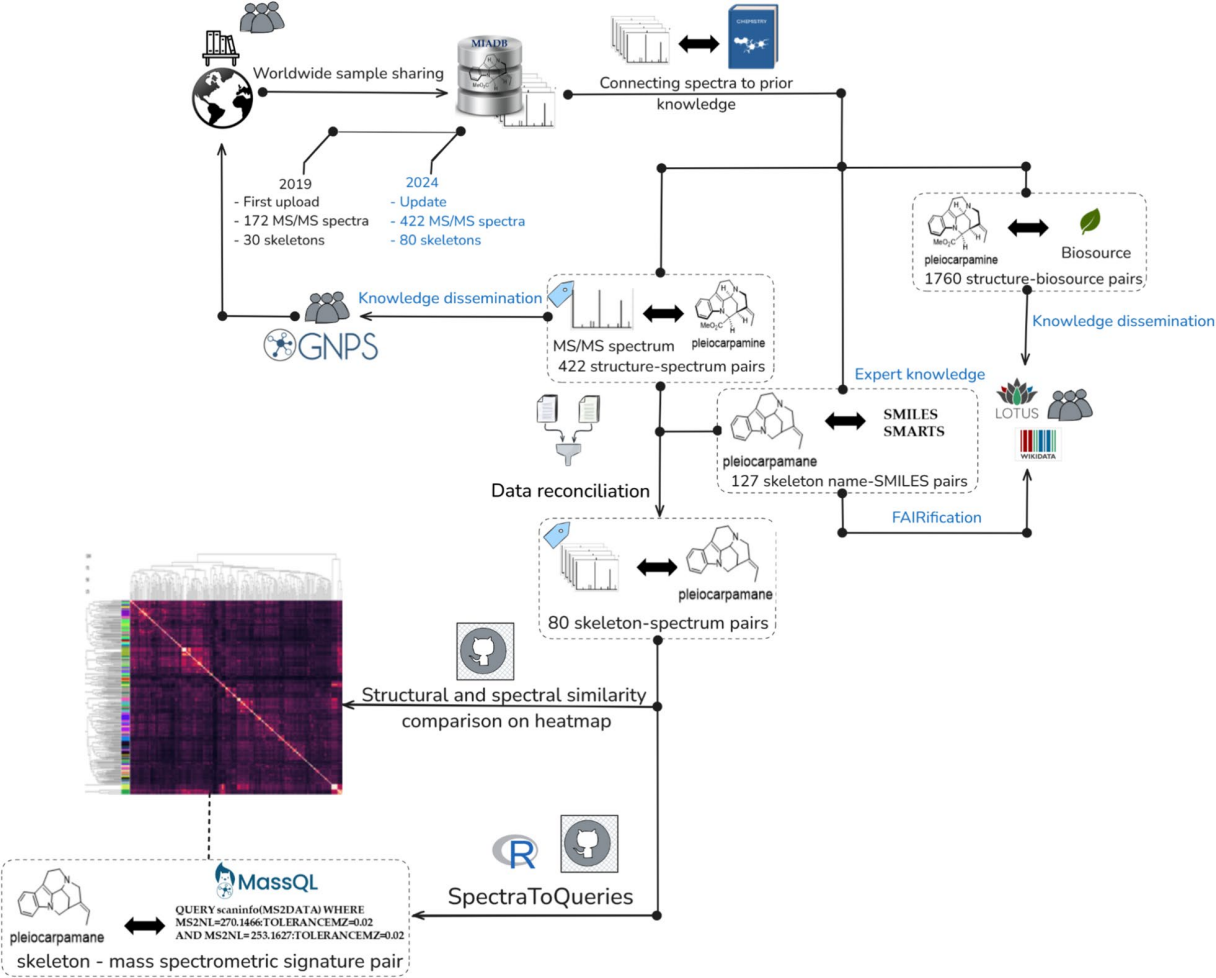
Overview and philosophy of the workflows developed in this work

Monoterpene indole alkaloids (MIA) are undeniably the most chemodiverse subfamily of indole alkaloids [1, 2]. With presumably more than 3000 structurally unique compounds, these polycyclic alkaloids have attracted contemporary attention from the organic chemists and the cheminformatics communities [3]. From a chemical perspective, MIAs offer a vast array of monomers (2300+), further enlarged by the occurrence of far more complex oligomeric representatives, of which we currently know 703 dimers, 13 trimers, and 1 tetramer [2]. To identify them in plant extracts, mass spectrometry (MS) has been the most widely-used technology so far, with tandem mass spectrometry (MS/MS) becoming increasingly used to support structural annotation and network-based analysis [4]. The ten past years saw the development of a worldwide sharing effort of tandem mass spectrometry data through the Global Natural Products Social platform (GNPS [5]). Accordingly, public spectral libraries have grown in size over the past decade to include hundreds of thousands to millions of MS/MS spectra and tens of thousands of compounds, forming an important knowledge base for the interpretation of metabolomics experiments [5, 6]. In 2019, eight prominent laboratories renowned for their commitment in MIA chemistry shared their historical collections, leading to the implementation of a MS/MS spectral knowledge base dedicated to this family of natural products (NPs), that we named Monoterpene Indole Alkaloids DataBase (MIADB) [7]. The MIADB contained MS/MS data of 172 standard compounds, comprising 128 monomers and 44 dimers and covered more than 70% of the known MIA skeletons. The MIADB has been uploaded to the GNPS [5] and MetaboLights [8]. This repository still constitutes the largest MS/MS spectral knowledge base dedicated to this emblematic family of NPs. Almost 6 years later, new and established collaborations collected 250 new MIAs that have been appended to the MIADB, reaching 422 MS/MS spectra with full structural annotations. Satisfyingly, this update enhanced the chemical space coverage from 30 to 80 skeletons (Fig. 1, Figs. S12 and S13).

The purpose of this work is to announce the update of MIADB and the submission of the corresponding MS/MS spectra on the GNPS platform, as well as the uploading of the associated metadata for these molecules, including the corresponding biological source and related references on LOTUS (1760 triples) [9]. Moreover, advances and/or democratization in computational mass spectrometry [10] and cheminformatics [11] including mass spectra similarity indices, molecular fingerprints, and domain-specific language-based query fueled our endeavors to use this unique MIA spectral space as a starting point to extract and disseminate valuable chemical knowledge for the community. To reach this goal, expert knowledge-informed algorithms have been designed and are shared herein (Fig. 1) [10].

## Methods

### Sample preparation and data acquisition

#### Pure compounds

Samples were dissolved at a concentration of approximately 1 mg/mL in MeOH or DMSO or in a CH_2_Cl_2_/MeOH (50/50, *v*/*v*) mixture, depending on their individual solubility.

#### Plant extracts preparation

Aerial parts of 75 plant species, most of which are known to be MIA producers, were collected in China, Costa Rica, Ecuador, Laos, Nigeria, Gabon, Malaysia, Mongolia, Myanmar, Nicaragua, Solomon Islands, and Vietnam. Samples were extracted with MeOH or 95% EtOH after drying and pulverizing. The extraction solvents were immediately removed by freeze-drying, and the dried extracts were stored at 20 °C until being analyzed. The samples were authenticated by local collectors, and voucher specimens are deposited in the International Biological Material Research Center of the Korea Research Institute of Bioscience and Biotechnology, together with the extract library.

The plant extracts were suspended in H_2_O and acidified using HCl to reach a pH range of 2–3. These acidic aqueous phases were extracted with EtOAc, prior to being treated with NH_4_OH to reach a pH value of 11–12. The basified aqueous layers were extracted using CH_2_Cl_2_ to obtain an alkaloid-rich fraction. The 75 selected plant extracts were then dissolved at a concentration of approximately 1 mg/mL in MeOH.

#### LC–MS/MS analyses

Exact mass LC–MS/MS data were recorded using an Agilent 1290 Infinity II UHPLC coupled to an Agilent 6546 hybrid quadrupole time of flight (QTOF) mass spectrometer (Agilent Technologies, Santa Clara, CA ,USA) equipped with an ESI source, operating in positive ion mode with data-dependent MS/MS acquisition. A BEH Acquity® C_18_ UPLC column (2.1 × 100 mm; i.d. 1.8 µm, Waters Co., Milford, MA, USA) was used. A 15 min method using a flow rate of 0.5 mL/min was applied including a 11 min linear gradient from 5% B (A: Milli-Q® H_2_O + 0.1% UHPLC-grade formic acid, B: UHPLC-grade MeCN + 0.1% UHPLC-grade formic acid) to 100% B, a 2 min washing at 100% B and a 2 min equilibration step at 5% B. Column and sampler temperatures were set at 40 °C and 15 °C, respectively. MS scans were recorded from *m/z* 100 to 1200 (3 spectra/sec). Source parameters were set as follows: capillary temperature at 320 °C, source voltage at 3500 V, sheath gas flow rate at 9 L/min. MS^1^ and MS^2^ scans were operated in full-scan mode from *m/z* 100 to 1200 (0.3 s scan time) with a mass resolution of 60,000 at *m/z* 922. In the positive-ion mode, purine C_5_H_4_N_4_ [M+H]^+^ ion (*m/z* 121.0509) and the hexakis(1*H*,1*H*,3*H*-tetrafluoropropoxy)-phosphazene C_18_H_18_F_24_N_3_O_6_P_3_ [M+H]^+^ ion (*m/z* 922.0098) were used as internal lock masses. Data collection was carried out using a data dependent acquisition (DDA) mode, where an MS^1^ scan was followed by MS^2^ scans of the 3 most intense ions above an absolute intensity threshold of 10,000 counts. Selected parent ions were fragmented at a collision energy set at 50 eV and an isolation window of 1.3 amu. A permanent MS/MS exclusion list criterion was set to prevent oversampling of the internal calibrant. LC-UV and MS data acquisition and processing were performed using MassHunter® Workstation software (Agilent Technologies).

### Data processing, algorithms and workflows

#### MS/MS data processing for spectral database generation

The pure compounds analysis resulted in obtaining 422 files in the Agilent .d format. The MS/MS data related to the signal of interest of each file were subsequently converted into a .mgf file using a tailored intensity threshold thanks to the dedicated “Export” option of the MassHunter® software (.mgf files available at https://github.com/spectra-to-knowledge/MIADB-data-files/tree/main/GNPS_upload).

#### MIADB molecular networking

These 422 MS/MS data files were then uploaded onto the GNPS platform for subsequent classical molecular networking analysis. To do so, a molecular network was created using the online workflow (https://ccms-ucsd.github.io/GNPSDocumentation/) on the GNPS website (http://gnps.ucsd.edu). The data was filtered by removing all MS/MS fragment ions within ± 17 Da of the precursor *m/z*. MS/MS spectra were window filtered by choosing only the top 6 fragment ions in the ± 50 Da window throughout the spectrum. The precursor ion mass tolerance was set to 0.02 Da and a MS/MS fragment ion tolerance of 0.02 Da. A network was then created where edges were filtered to have a cosine score above 0.9 and more than 6 matched peaks. Further, edges between two nodes were kept in the network if and only if each of the nodes appeared in each other’s respective top 10 most similar nodes. Finally, the maximum size of a molecular family was set to 100, and the lowest scoring edges were removed from molecular families until the molecular family size was below this threshold. The spectra in the network were then searched against GNPS spectral libraries. The library spectra were filtered in the same manner as the input data. All matches kept between network spectra and library spectra were required to have a score above 0.7 and at least 6 matched peaks. The resulting job can be accessed via the following link: https://gnps.ucsd.edu/ProteoSAFe/status.jsp?task=fca55f3aa80d421fb0d090099234983d.

#### Skeletons structural similarity study using the Tanimoto index

**Fig. 2:**
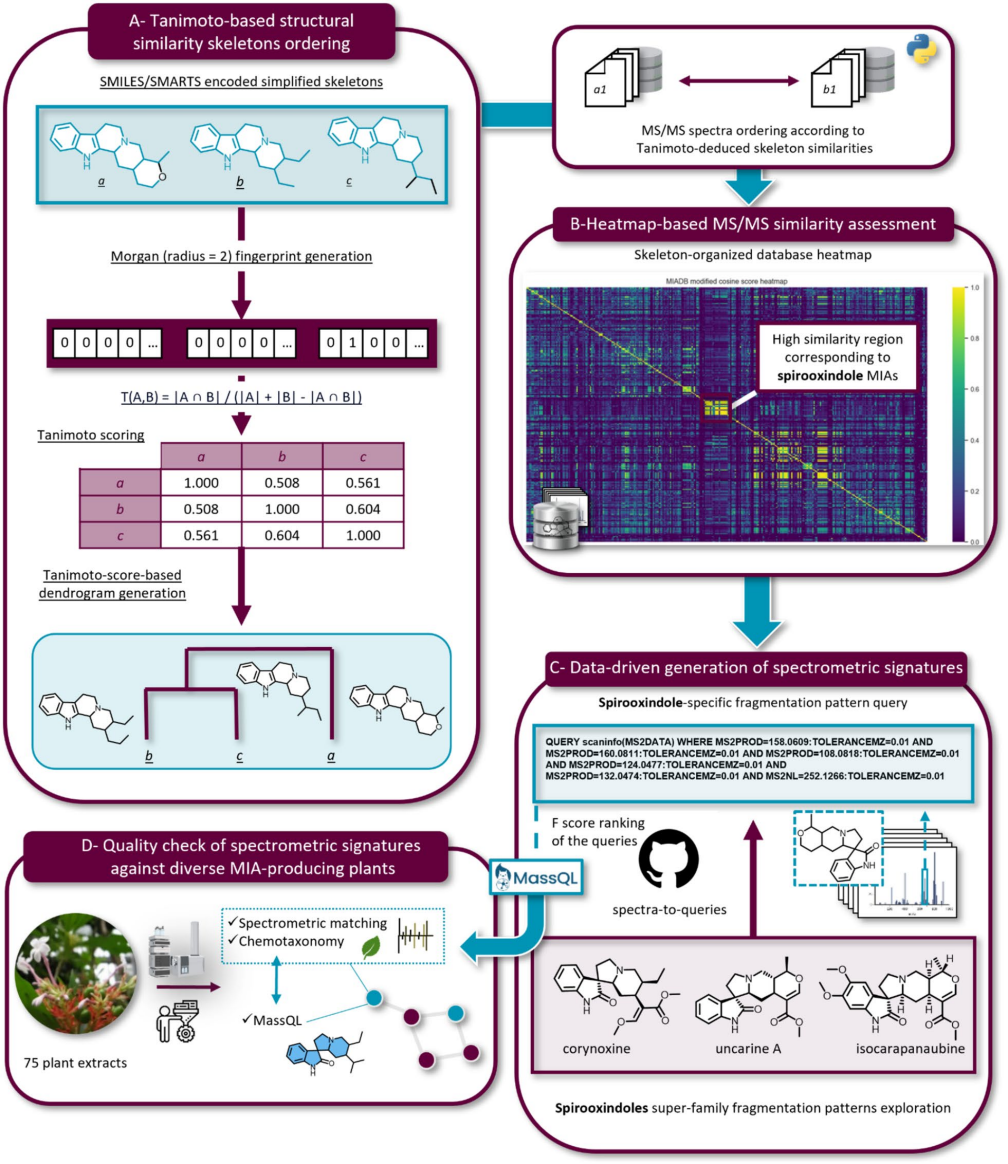
General workflow: data-driven generation of spectrometric signatures based on MS/MS spectral similarity shared across structurally related-skeletons and subsequent quality check on a diverse plant extracts dataset

Prior to undertaking the Tanimoto-based comparison of the different skeletons, the difficulties related to the presence of different skeletons within multimeric compounds (e.g., [vinblastine contains cleavamine and aspidosperma, and tabernamine contains vobasine and iboga]) were circumvented by only considering a subset of the compounds containing molecules with a single MIA unit from MIADB (321 compounds). These 321 compounds were then assigned to one of the 80 expert-defined skeleton (Fig. S12 and Fig. S13), intended to mainly retain the gross, unsubstituted backbones. Each of these 80 skeletons was paired to a SMILES (obtained through the dedicated “Copy as SMILES” option of the ChemDraw^®^ software) and a SMARTS (obtained from previously generated SMILES using the rdkit Python library, the executable code is made available at: https://github.com/spectra-to-knowledge/miadb-visualization/blob/main/notebooks/smiles-to-smarts.py). SMILES and SMARTS of the skeletons appear in Table S1. These SMILES strings were then converted into molecular objects via the “MolFromSmiles” module included in the rdkit library (v.2023.3.1) and fingerprints were generated from these molecular objects using the Morgan algorithm [12] (size = 2, nBits = 2048). The fingerprints were finally compared through Tanimoto scoring and results were displayed as a dendrogram (Fig. 2), illustrating the retained structural affiliations between each SMILES-encoded skeleton for MS/MS spectra ordering across the axes of the different heatmaps (Figs. S2–S11). The code used to perform the skeletons structural similarity study is available using this link: https://github.com/spectra-to-knowledge/miadb-visualization/blob/main/notebooks/generate_tanimoto_heatmap_and_dendrogram.py.

#### Heatmaps generation

The data file collating the subset of MS/MS spectra for the aforementioned 321 monomeric MIAs retained for heatmaps generation is available at: https://github.com/spectra-to-knowledge/miadb-visualization/blob/main/src/miadbviz/data/MIADB-monomers.mgf. Each spectrum was associated with a skeleton metadata corresponding to the chemist-defined compound skeleton. The fragment intensities of the pure compounds were then normalized using the “normalize_intensities” filter of the matchms Python library [13]. modified cosine score, classic cosine score, Spec2Vec [14] and MS2DeepScore [15] matrices were then obtained using the dedicated Python libraries for all the MIADB (all codes regarding the generation of modified cosine score, Classic cosine score, Spec2Vec and MS2DeepScore matrices are available here: https://github.com/spectra-to-knowledge/miadb-visualization/blob/main/notebooks/generate_heatmap.py). Results were displayed as heatmaps using the seaborn library [16] (Figs. S1 to S11).

#### Spectral signature extraction from the subset of monomer-enriched MS/MS spectra

For all the preprocessing of the spectra, a (10 mDa or 25 ppm) tolerance was used. First, spectra were deisotoped and concurrent fragments within the tolerance were reduced, keeping only the most intense. Fragments with a *m/z* value above (and including) the precursor were removed. Then, intensities of the fragments were normalized to the highest fragment and *m/z* values were harmonized among all spectra of the library. Neutral losses were computed from the precursor ion mass, allowing only for losses from ions smaller than the precursor. Spectra were then binned (with the above-mentioned tolerance) and the *m/z* value was calculated back to obtain peaks and losses matrices with sufficient mass precision, then rounded to 4 decimals. For signature extraction, only signals occurring in at least 3 spectra were kept as candidates. If a signal was found in all members of a group, it was directly considered. If not, an *F*_*2*_ score was calculated, and only the 10 highest scores were kept.

$$F_{2 } = \left( {1 + 2^{2} } \right) * \left( {Precision* Recall} \right)/ \left( {\left( {2^{2} *Precision} \right) + Recall} \right)$$

The choice of β = 2 was made to favor recall over precision, as the combination of multiple diagnostic signals can only decrease recall and increase precision. An optional filter for minimal score value was made available (0 by default). For example, the fragment with a *m/z* ratio of 124.0429 was found in 16 spectra in the updated MIADB, of which 11 genuinely corresponded to ajmalicine spirooxindoles (out of 12 ajmalicine spirooxindoles present in the library). In such a case,

$$F_{2 } = \left( {1 + 2^{2} } \right) * \left( {11/16 * 11/12} \right)/ \left( {\left( {2^{2} *11/16} \right) + 11/12} \right) = 0.90$$

was a high score and was considered for further evaluation. From the remaining signals, for each skeleton family containing at least 5 members, all possible additive combinations of maximum 10 signals (to avoid too complex queries and long computation time) were generated and tested back against the original (non-processed) library, in addition to the signals with a specificity of 1. Performance was evaluated using an F_0.5_ score and the queries leading to the best performance (with ties) were kept for further use.

#### LC–MS/MS molecular networking and annotation of plant extracts

75 plant extracts selected on the basis of their diverse MIA content and 14 blanks were analyzed, resulting in 89 Agilent .d MS/MS data files that were converted into .mzXML files via MSConvert software, part of the ProteoWizard package [17]. The .mzXML files were then uploaded to MZmine 3 v3.9.0 [18] and processed with the following parameters: the mass detection was realized by keeping the noise level at 6.0E3 (MS^1^) and 3.0E1 (MS^2^). The ADAP [19] chromatogram builder was used using a minimum group size of scans of 4, a group intensity threshold of 6.0E3, a minimum highest intensity of 1.2E4 and *m/z* tolerance of 10 ppm. The local minimum feature resolver was then used with the following settings: MS/MS scan pairing (MS^1^ to MS^2^ precursor tolerance 0.01 Da or 10.0 ppm, retention time filter = use feature edges, minimum relative feature height = 25.0%, minimum required signals = 1, minimum signal intensity (relative) = 1.0%), dimension = t_R_, chromatographic threshold = 95%, minimum search range t_R_(min) (absolute) = 1.0, minimum relative height = 1.0%, minimum absolute height = 1.0E3, min ratio of peak top/edge = 0.00, peak duration range (min) = 0.00–1.0, minimum scans = 4. Isotopologues were grouped using the ^13^C isotope filter algorithm with a tolerance of 0.00 Da or 10 ppm and a t_R_ tolerance of 0.15 min, the maximum charge was set to 2 and the representative isotope was set to most intense. Peak alignment was performed using the join aligner module (*m/z* tolerance = 10 ppm, weight for *m/z* = 50, weight for t_R_ = 50, t_R_ tolerance 0.15 min). Gap filling was performed using the peak finder module (*m/z* tolerance = 10 ppm and intensity tolerance = 1%, t_R_ tolerance = 0.15 min and min scan = 1). Blank features were removed using the feature list blank subtraction (min # detection in blanks: 1, quantification: height, ratio type: maximum). Finally, data were filtered using the filter list rows filter module (standard settings, only peaks with MS/MS were kept). The .mgf and .csv (for t_R_ and areas) files were exported using the dedicated Export Molecular Networking files built-in option (job ID = https://gnps.ucsd.edu/ProteoSAFe/status.jsp?task=ec37fe7e20764d38924c881ab9dd006e). The complete batch file in .xml format is available at the following repository: https://github.com/spectra-to-knowledge/miadb-supplementaries/blob/main/mzmine_params.xml. This molecular network was further enriched with a Taxonomically Informed Metabolite Annotation (TIMA, version 2.10.0) following the methodology of [18]. Parameters used for this complementary annotation were the software default ones [19]. The full list of parameters is accessible at: https://github.com/spectra-to-knowledge/miadb-supplementaries/blob/main/tima_parameters.yaml, and the corresponding outputs are available via this Zenodo link: https://zenodo.org/records/14148771.

Additionally, previously extracted spectral signatures of the ajmalicine spirooxindole, corynantheane spirooxindole, and ajmalicine/corynantheane spirooxindole skeletons were queried against our dataset of 75 plant extracts using MassQL. Parameters and outputs for these queries are accessible via the following GNPS job links:

- ajmalicine spirooxindole skeleton spectral signature query: https://gnps.ucsd.edu/ProteoSAFe/status.jsp?task=3e385e5bd89e434e910acca9b9d0f210
- corynantheane spirooxindole skeleton spectral signature query: https://gnps.ucsd.edu/ProteoSAFe/status.jsp?task=737b11b4de904808af7776a1c9a4e9ed
- ajmalicine/corynantheane spirooxindole skeletons spectral signature query: https://gnps.ucsd.edu/ProteoSAFe/status.jsp?task=4cd9ff97d0fd45adb3e2a4e9dd8be4a5

#### Pie charts generation

The entire metadata related to the molecular network annotated by the three aforementioned MassQL queries were exported as a .tsv file using the built-in Cytoscape function. Individual Excel spreadsheets (accessible here: https://github.com/spectra-to-knowledge/miadb-visualization/tree/main/src/miadbviz/data) were generated to retain only the non-null results of a single MassQL query, i.e., *ajmalicine-spirooxindole*, *corynantheane-spirooxindole*, and *ajmalicine/corynantheane-spirooxindole*. A Python script (available at: https://github.com/spectra-to-knowledge/miadb-visualization/blob/main/notebooks/generate_pie_chart.py) was created to display the distribution of skeletons across plant genera in the form of pie charts. Notably, in cases where multiple extracts of the same plant appeared in the dataset, columns with identical plant names were merged, and the average ion intensities were calculated and retained.

## Results and discussion

As a way to guide the readers throughout the different steps of our study, a general workflow is illustrated in Fig. 2.

### Mining MIA skeletons

In 2010, a seminal effort, collated in the *Dictionary of Alkaloids* and focusing on the impressive chemodiversity of monomeric MIAs, revealed that they can be divided into 42 skeletons [20]. The past decade saw intense phytochemical efforts dedicated to this family of natural products leading to the description of new members [21–24]. Since this first foray in MIA skeletons inventory, we conducted extensive database explorations using a combination of keywords such as “new skeleton”, “unprecedented carbon skeleton” against *SciFinder Scholar* and the *Dictionary of Natural Products*. This investigation resulted in a compendium of 127 skeletons that we share, through this work, for the first time in a machine-readable format (Table S1, Figs. S12 and S14). The 42 preceding skeleton names have been reused and 85 new names have been proposed by us for the additional representatives starting from the name of the first described natural product. 80 skeletons are represented in the MIADB and serve as a foundation for connecting chemical expert knowledge to the MIADB spectral data.

### Investigating the hypothesis of spectral and structural similarity equivalence in MIA skeletons

Our first goal was to evaluate the spectral similarities across the MIADB to identify MS/MS clustering trends within this diverse chemical family. Our initial attempt involved using molecular networking on the MIADB (Fig. S19). Nodes were annotated with colors representing their skeletons (Fig. S12). Our first observation was that most ions tended to cluster with ions from the same skeleton or with ions from related skeletons (Figs. S19–S21). After conducting several tests with different GNPS parameter values, it appeared, as could be expected, that molecular networking results heavily depended on factors like minimum modified cosine scores and topK values. Consequently, any conclusions drawn were only meaningful for the specific parameters selected [25]. Additionally, while molecular networking provides a valuable visual tool for observing ions with the most similar MS/MS spectra, it does not highlight relationships between ions revealing less pronounced MS/MS similarities and thus may not represent the best approach for capturing the results of a systematic pairwise comparison within large MS/MS datasets, as is the case here. To address these limitations, we opted for a heatmap visualization. The first attempt was performed using the modified cosine score on the randomly listed spectroscopic files related to the 422 MIA MS/MS spectra. However, the results were difficult to interpret as the ‘high-scores’ (above 0.7) were scattered across the heatmap (Fig. S1). To remedy this, we decided to order our compounds along the axes according to their structural similarity. In order to perform a scaffold-centric comparison of structures and avoid possible biases associated with substituents that may create non-specific similarities between different skeletons, compounds were assigned to a specific MIA subtype (Figs. S12 and S13), for which tailored SMILES were generated (Table S1) to encompass all the individual molecules belonging to a given skeleton.

#### Tanimoto-based similarity assessment of the MIADB-skeletons

**Fig. 3:**
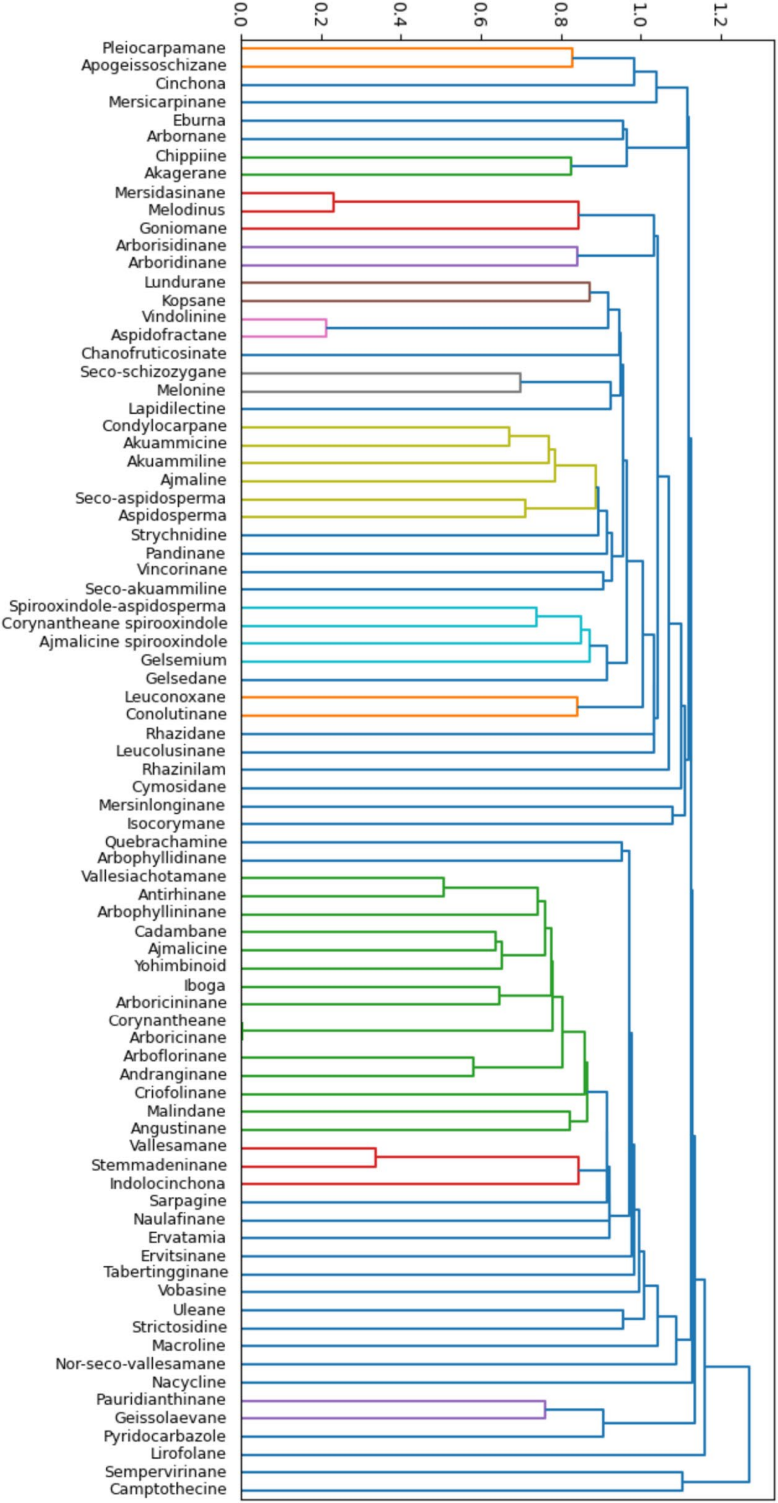
Structural similarity dendrogram between generic skeletons designed for compounds included in MIADB (Generic skeleton structures, number of representatives and SMILES are provided in Fig. S12 and Fig. S13 and Table S1). It should be noted that the Gelsemium skeleton had first been retained as a generic MIA skeleton based on its appearance in the *Dictionary of Alkaloids* in 2010 [20]. However, we have the feeling that this skeleton should be split into different subtypes to best capture the structural diversity currently referred to as ‘Gelsemium’. See ‘Gelseleginane’, ‘Gelsemamidane’, ‘Gelseminane’, ‘Humanteninane’, ‘Gelsedane’ and ‘Isohumanteninane’ for further details (Fig. S14)

After generating SMILES representations for the skeletons of the MIADB, we calculated pairwise Tanimoto coefficients to assess structural similarity among the 80 encoded skeletons (Fig. 2A). This similarity analysis was then visualized as a dendrogram (Fig. 3), illustrating the relationships among the skeletons.

Although it is difficult to comment on the classification of these various skeletons exhaustively, it can be noted that their classification turned out to be largely similar to what a ranking based on chemical expert knowledge would have produced. There are, however, a few examples where Tanimoto coefficients revealed a greater distance between two skeletons compared to the NP chemist's point of view. This was the case for the skeletons of sarpagine and vobasine (the latter is often regarded as a simple indole acyl of the former), but also for the appendages of aspidosperma and aspidofractane (the latter differing from the former only by a single additional bond) (Fig. S12). In both cases, it appears that these pairs of skeletons revealed a limited degree of MS^2^ pattern similarity, supporting the Tanimoto-based classification.

#### Heatmap-based MS/MS similarity index assessment

The recent development of several MS/MS similarity indices [26] led us to assess their ability to relate spectrometric information to structural similarity applied to the monomeric MIADB subset of 321 spectra. Accordingly, MS/MS spectral similarity heatmaps were generated using the modified cosine score [5, 27, 28] (Fig. 2B, Figs. S2 and S3), the Spec2Vec score [14] (Figs. S4 and S5), the MS2DeepScore [15] (Fig. S6), and the classic cosine score [26, 27, 29] (Fig. S7). An inspection of those heatmaps revealed that the modified cosine score outperformed both the Spec2Vec score and the MS2DeepScore in discriminating MIAs with structurally unrelated skeleton. Indeed, most scores obtained using Spec2Vec or MS2DeepScore were notably high (above 0.7) across the heatmaps, which limited their ability to discriminate between different skeletons. While these scores proved highly effective in highlighting spectral similarities within compounds of the same natural product (NP) chemical class, they lacked the specificity required for differentiating subtypes of monoterpene indole alkaloids (MIAs). Interestingly, the modified cosine score also surpassed the classic cosine score in highlighting spectral similarities between representatives of the same skeleton type (Figs. S2 and S7), making it more suitable for further investigation of skeleton-dependent MS/MS-based landmarks.

#### MS/MS-based structural deductions

Having classified the compounds according to their skeleton along the axes, several hotspots were observed near the diagonal (Fig. 2B and Fig. S2), providing compelling evidence of significant spectral similarities within identical or related skeletons. This observation is even more pronounced when only similarity scores above 0.9 are displayed (Fig. S3) highlighting two structurally homogeneous groups of compounds with high spectrometric similarity that will be further discussed.

**Fig. 4:**
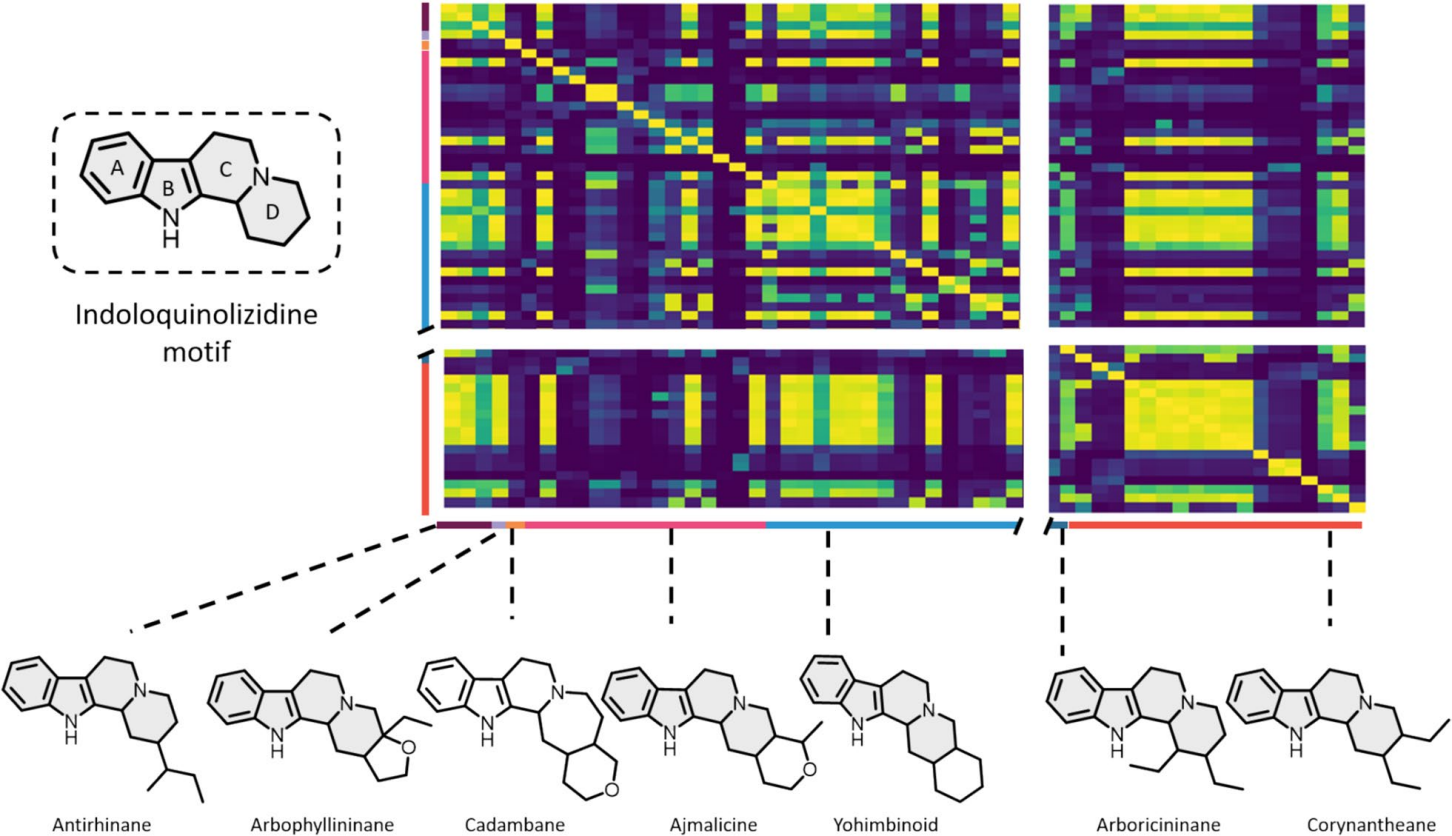
Expanded view of the modified cosine heatmap region revealing a high similarity score (Fig. S2A). The various skeletons of compounds appearing in the region, most of which reveal an indoloquinolizidine motif, are shown below and color-coded on the left-hand side

Closer examination of these two groups of compounds with a high degree of MS/MS similarity revealed a first set of structurally related tetracyclic or pentacyclic skeletons featuring a common indoloquinolizidine motif (viz*.* antirhinane, corynantheane, ajmalicine and yohimbinoid, to only retain skeletons represented by more than three different compounds) (Fig. S2A for an enlarged view). Within a given skeleton, it appears that some individual representatives show a limited degree of MS/MS similarity to other compounds belonging to the same structural family (Fig. 4).

**Fig. 5:**
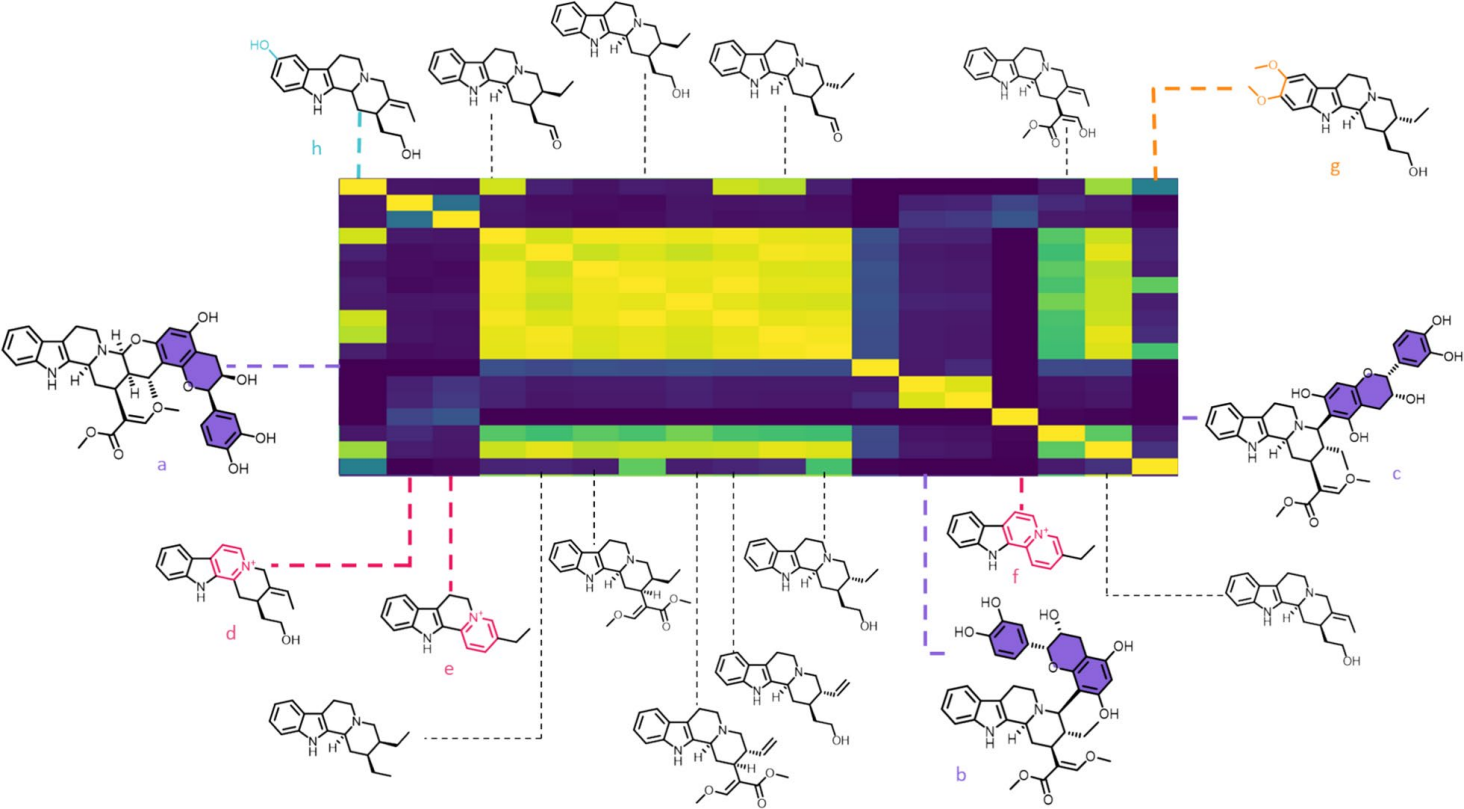
Closer view of the modified cosine heatmap disclosing the structures of all corynantheane-type MIA members included in the updated MIADB. Structural features likely to be responsible for disparities in MS/MS landmarks compared with other representatives of the series are marked in color

A detailed structural examination of these compounds could reveal certain structural features possibly responsible for their different fragmentation pathways. In the specific example of corynantheane-type MIAs, some compounds appear to reveal a limited degree of MS/MS similarity (Fig. 5) when (i) the backbone is substituted by a massive moiety of different biosynthetic origin [e.g., epicatechocorynantheidine (Fig. 5a) and epicatechocorynantheines A–B (**b**–**c**) carrying a foreign epicatechin component], (ii) aromaticity appears on the C ring [3,4,5,6-tetradehydrogeissoschizol (Fig. 5d)], D ring [6,7-dihydroflavopereirine, (Fig. 5e)] or both C and D rings [flavopereirine, (Fig. 5f)], or (iii) A ring is disubstituted by oxygenated moieties [e.g., the 10,11-dimethoxylated ochropposinine (Fig. 5g)]. It may also be noted that compounds which accumulate several minor differences with other compounds in their family [e.g., 10-hydroxygeissoschizol (Fig. 5h), which has both a 10-OH group and an ethanolic side chain] may also reveal different MS/MS data at the end compared with these other members. Such structural variations seem to exert a greater influence on spectrometric similarities than their belonging to a given skeleton. For example, the dimethoxylated corynantheane-type ochropposinine (**h**) shows a high degree of MS/MS similarity with dimethoxylated ajmalicine analogues such as isoreserpiline and rauvanine, and with dimethoxylated yohimbinoids like seredine and seredone (Fig. S10). Conversely, ochropposinine shows a limited degree of MS/MS similarity to other corynantheane-type MIAs, with the exception of corynantheidol derivatives which share a common ethanolic side chain. In this context (i.e., pronounced MS/MS similarities with compounds from different skeletons), it would seem that MS/MS behavior is more likely to capture certain sharp structural features than to define the membership of such a compound to a precise skeleton.

**Fig. 6:**
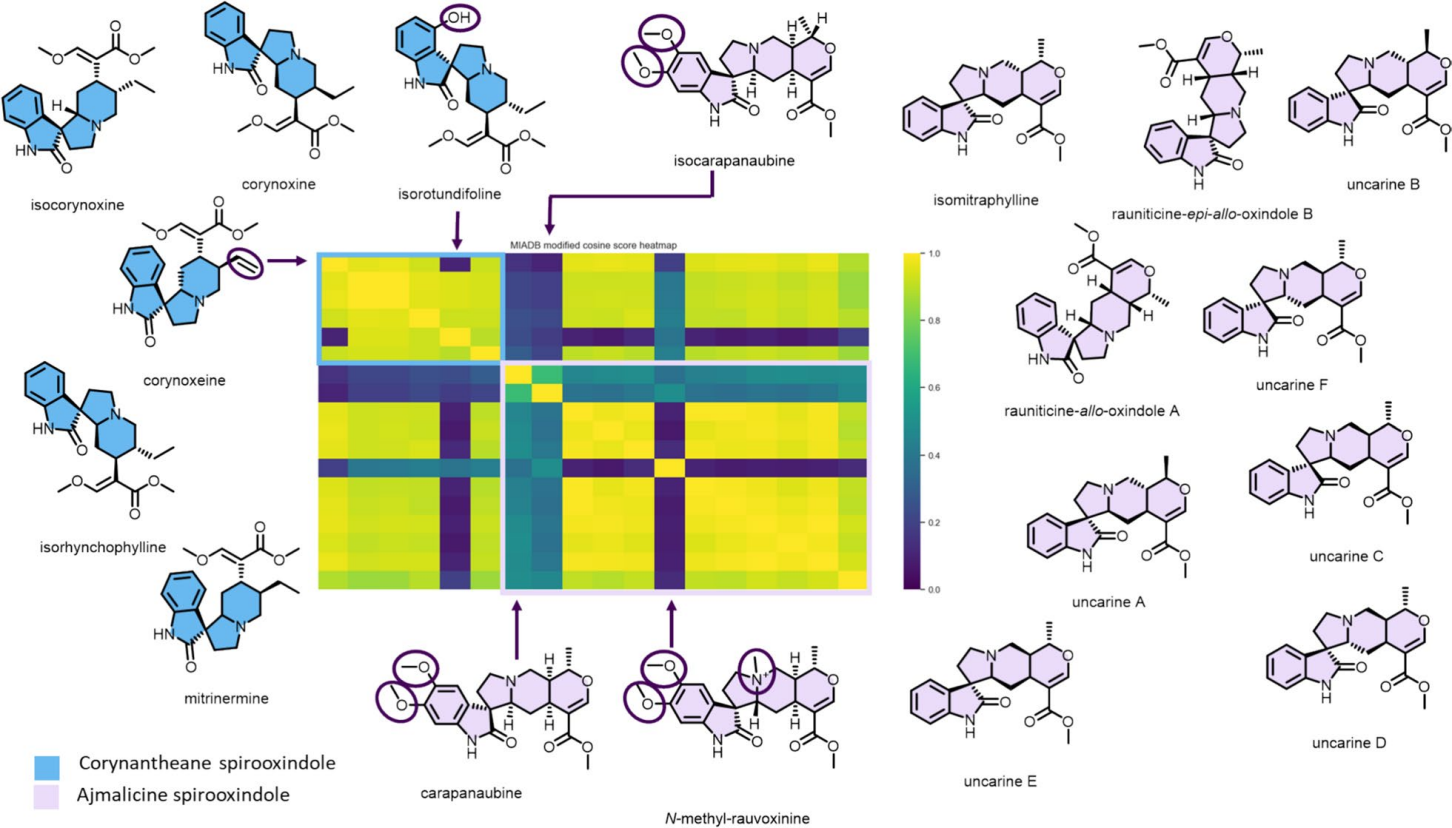
Closer view of the modified cosine heatmap region containing the ajmalicine spirooxindoles and corynantheane spirooxindoles, along with their chemical structures (Fig. S2B). Structural features thought to lead to different fragmentation patterns compared with other representatives of their families are highlighted. Heatmap regions related to both skeletons are delimited by color tagged rectangles

A second region of notable interest in the heatmap groups together spirooxindole-type MIAs, namely ajmalicine spirooxindoles and corynantheane spirooxindoles, two types of skeletons which, although traditionally distinct, are closely related to each other (Fig. 6). As indicated in the previous section, our series of compounds reveals that the methoxylation status of the A ring exerts a significant influence on the MS/MS fragmentation pattern of ajmalicine spirooxindoles (heatmaps reveal a pronounced difference between the MS/MS spectra of the dimethoxylated carapanaubine or isocarapanaubine and those of their A-ring unsubstituted analogues).

The correlation between structural and spectral similarity within skeletons is therefore demonstrated by this study, although it is not homogeneous throughout the database. All these observations led us to look for potential spectrometric signatures responsible for the coherence between structural and spectral similarities within a skeleton/related skeletons in a data-driven manner.

### Extracting and evaluating MIA skeleton-specific spectrometric signatures

#### Skeleton-specific spectrometric signature extraction

Following the conclusions reached in the previous section, a skeleton-specific spectrometric signature extraction algorithm, called SpectraToQueries, has been set up and implemented (https://github.com/spectra-to-knowledge/spectra-to-queries/blob/main/inst/scripts/spectra_to_queries.R). The latter intends to look for spectrometric patterns (fragments and/or neutral losses) shared specifically by representatives of the same skeleton (or by compounds of structurally related skeletons). That being said, this tool expects MS/MS data acquired at an optimized collision energy. In this work, a 50 eV value was selected based on preliminary assays conducted at different collision energies on a structurally diverse subset of MIAs, including various types of oligomers and monomers, as well as molecules belonging to different skeletons. Starting from the MIADB spectrometric space, skeleton-specific spectrometric signatures were extracted and combinations of the latter were generated and tested systematically to serve as queries. Remarkably, three skeleton-specific queries related to lapidilectine, ajmalicine spirooxindole, and corynantheane spirooxindole reached an F-score > 95%. Best generated queries and their respective F-scores are available at https://github.com/spectra-to-knowledge/spectra-to-queries/tree/main/data/interim and Table S3. Since ajmalicine spirooxindole and corynantheane spirooxindole skeletons had already been pinpointed as MS/MS similarity hotspots (Fig. 6), further evaluation efforts will be directed toward those specific skeletons (Fig. 2C). While their creation was based on a set of chemically related skeletons, their applicability to a real case scenario encompassing a wider chemical diversity was further assessed against a chemodiverse extract library comprising 75 extracts obtained from plants pertaining to families known to produce MIAs.

#### Assessing the performances of skeleton-specific spectral signatures on a collection of 75 plant extracts

To assess the ability of our generated skeleton specific-signatures to provide accurate structural information, a massive molecular network was generated from 75 UPLC-HRMS/MS analyses of various plants pertaining to MIA-producing families (Apocynaceae, Rubiaceae, Loganiaceae) (Table S2). This collection can be presumed to encompass a wide range of structurally diverse MIAs, likely to be annotated by any of our tentative skeleton-specific spectral signatures. On the contrary, some of the plants studied here are not known to produce MIA, which we decided to include herein as negative controls. The above-mentioned skeleton-spectrometric signatures were exploited with the recently introduced domain specific language-based query MassQL [30] to query the 75 plant extracts dataset. MassQL enables LC–MS/MS data sets to be queried for specific features, including MS^1^/MS^2^ precursor *m/z*, MS/MS fragments and their intensity, neutral losses, retention time, and mass tolerance. The likeliness of MassQL-based annotations was further evaluated on the obtained molecular network, against annotations obtained from experimental matches with GNPS spectral libraries and provided by *Taxonomically Informed Metabolite Annotation* (TIMA) [31], and also assessed by regards to chemotaxonomic considerations for unannotated features (Fig. 2D).

#### Evaluation of the ajmalicine spirooxindole query

Integration of the ajmalicine spirooxindole MassQL query yielded 78 tags, 16 of which also benefited from experimental annotation by GNPS (with two sets of duplicates, hence a total of 14 unique annotations). Overall, it appears that 7 nodes out of 16 were annotated as ajmalicine spirooxindoles by the GNPS spectral libraries, the accuracy of this query is therefore of 43.75%. In the whole molecular network, 7 nodes were annotated as ajmalicine spirooxindoles so that the recall for this query is 100% on the GNPS-annotated ions (Table S4). Interestingly, three additional nodes were annotated as representatives of the structurally related corynantheane spirooxindole-type MIAs, in line with the high degree of MS/MS similarity between these two structural classes described earlier (Fig. 6). Skeleton details related to the other GNPS hits, corresponding to a few miscellaneous MIA skeletons, can be found in the supporting information (Table S5).

To further evaluate the likeliness of our MassQL requests by regards to chemotaxonomic consistency, we analyzed the plant origin of the different nodes that had been tagged. Plotting the ion intensity of all MassQL-annotated nodes against the genus of the plant source identified three predominant producing plant genera: *Rauvolfia* (29.6%), *Mitragyna* (25.5%), and *Uncaria* (19.7%) (Fig. S15). Similarly, the intensity ranking of all nodes annotated by MassQL reveals that these plant genera are the most represented at the scale of individual ions (Tables S5 and S6). These conclusions are in line with current knowledge of the chemistry of these different plant genera [32–34].

#### Evaluation of the corynantheane spirooxindole query

The MassQL command aimed at hooking corynantheane spirooxindole-type MIAs yielded analogous results. Out of 38 nodes being tentatively MassQL-annotated as corynantheane spirooxindole-type MIAs, 7 benefitted from a tentative annotation against the GNPS spectral libraries. Three of them were provisionally identified as corynantheane spirooxindoles and two others had been annotated as possible ajmalicine spirooxindoles (Table S7). Ion intensities of these MassQL-annotated nodes related to the genera of producing plants reveal the dominance of *Mitragyna* (52.8%) and *Rauvolfia* (21.3%) among producers of the corresponding molecules, consistent with former literature reports (Fig. S16) [35]. Assessment of individual ion intensities reveals a similar trend (Table S8).

#### Towards a combined query

The difficulties encountered by our MassQL queries in distinguishing the corynantheane spirooxindole and ajmalicine spirooxindole skeletons led us to consider the possibility of developing a query capable of identifying either of these two closely related skeletons. This seems more realistic, especially considering the high degree of MS/MS similarities noted between the members of these two skeletons, as already highlighted in the heatmap displayed in Fig. 6. The resulting common spectrometric patterns were queried against our dataset, yielding 108 features annotated by MassQL. Eighteen such features could have been annotated against the GNPS repositories, 10 of which were of the corynantheane spirooxindole or the ajmalicine spirooxindole subtype (Table S9) determining an accuracy of 55.56%. Of the ions annotated by GNPS, 11 were either corynantheane spirooxindole or ajmalicine spirooxindole-type MIAs, determining a recall of 90.91% for this MassQL request. Once again, chemotaxonomic considerations supported the value of this MassQL command, as labeled ions are mainly found in plants producing such spiranic oxindoles: *Mitragyna* (28.7%), *Rauvolfia* (28.2%), and *Uncaria* (19.5%) (Fig. S17 and Table S10). These MassQL-based annotations also found support in some of the structure assignments proposed by TIMA, which have also retained a corynantheane spirooxindole or an ajmalicine spirooxindole constitution for certain nodes and/or compounds found in the same cluster (Fig. S18).

## Conclusion

The present work takes advantage of a major update of the MIADB, which now incorporates more than twice as much MS/MS data as its initial version submitted in 2019 (422 entries versus 172 initially). In this work, the MIADB spectral space served as a starting point for valuable chemical knowledge mining using well tailored chemoinformatics tools further enhanced with chemical expert knowledge. As such, 127 MIA skeletons have been defined and disseminated in machine-readable format for the first time. Evaluation of the spectral and structural similarities of MIA subtypes revealed that certain skeleton, such as ajmalicine spirooxindole, corynantheane spirooxindole, and a set of indoloquinolizidine-containing MIAs, exhibit a strong correlation between structural and spectral similarities. From this spectral similarity study, relevant specific MS/MS spectral signatures have been discovered for a few skeletons and permitted to establish reliable MassQL queries. The latter have been validated against a chemodiverse extract collection of MIA-producing plants. At last, while spectral libraries are often put forward to empower the next generation of machine learning tools in computational metabolomics, an additional layer of chemical expert knowledge, combined to well tailored tools, helps in mining and disseminating valuable information. We hope that the methodological aspect of our work will result in a mind shift among the metabolomics community concerning spectral libraries.

## Supplementary Information

Supplementary Material 1.

## Data Availability

All .d (Agilent), .mzXML, and .mgf datafiles in positive mode along with metadata and metabolite annotation tables of the 75 plant extracts dataset are available on the MassIVE repository under accession number MSV000096137, and with the following: 10.25345/C56Q1SV35. The 75 plant extracts molecular network job is accessible via this link: https://gnps.ucsd.edu/ProteoSAFe/status.jsp?task=ec37fe7e20764d38924c881ab9dd006e. Taxonomically Informed Metabolite Annotation outputs of the 75 plant extracts are accessible here: https://zenodo.org/records/14148771. All supporting data and materials related to the MIADB submission on the GNPS are available as follows: GNPS library link to the MIADB: https://gnps.ucsd.edu/ProteoSAFe/gnpslibrary.jsp?library=MIADB. All data used as input for heatmaps, dendrogram and pie charts are accessible here: https://github.com/spectra-to-knowledge/Data-processing-and-visualization/tree/main/Data. All codes used for data processing and visualization are available in the following GitHub repository: https://github.com/spectra-to-knowledge/Data-processing-and-visualization/tree/main/Codes. All resulting files (outputs) of the previous processing and visualization steps are included in this GitHub repository: https://github.com/spectra-to-knowledge/Data-processing-and-visualization/tree/main/Outputs. All tables depicted in Supplementary Material are accessible in .tsv formats via the following link: https://github.com/spectra-to-knowledge/mia-supplementaries/tree/main.

## Abbreviations

MeCN: Acetonitrile
CH_2_Cl_2_: Dichloromethane
DMSO: Dimethyl sulfoxide
FA: Formic acid
MeOH: Methanol
t_R_: Retention time
