# Supplementary material for: Translating community-wide spectral library into actionable chemical knowledge: a proof of concept with monoterpene indole alkaloids
Source: J Cheminform. 2025 Apr 28;17:62. doi: 10.1186/s13321-025-01009-0 (PMC12039057; doi:10.1186/s13321-025-01009-0)
Supplement: Supplementary file 1 — Supplementary Material 1. [file 13321_2025_1009_MOESM1_ESM.docx]

Supplementary Material

Translating community-wide spectral library into actionable chemical knowledge: A proof of concept with monoterpene indole alkaloids

Sarah Szwarc^1,✝^, Adriano Rutz^2✝^, Kyungha Lee^3^, Yassine Mejri^1,4^, Olivier Bonnet^5^, Hazrina Hazni^6^, Adrien Jagora^1^, Rany Berty Mbeng Obame^1^, Jin Kyoung Noh^7^, Elvis Otogo N’Nang^8^, Stephenie C. Alaribe^9^, Khalijah Awang^6^, Guillaume Bernadat^1^, Young Hae Choi^10^, Vincent Courdavault^11^, Michel Frederich^5^, Thomas Gaslonde^12^, Florian Huber^13^, Toh-Seok Kam^6^, Yun Yee Low^6^, Erwan Poupon^1^, Justin J.J. van der Hooft^14,15^, Kyo Bin Kang^3^, Pierre Le Pogam^1^* and Mehdi A. Beniddir^1^*

Affiliations

^1^Université Paris-Saclay: *Équipe, Chimie des Substances Naturelles, Université Paris-Saclay, CNRS, BioCIS, 17 avenue des Sciences, 91400 Orsay, France.*

^2^ETH Zurich:  *Institute of Molecular Systems Biology, ETH Zürich, 8093 Zürich, Switzerland.*

^3^Sookmyung Women’s University: *College of Pharmacy and Research Institute of Pharmaceutical Sciences, Seoul 04310, Republic of Korea.*

^4^Université Paris-Dauphine: *Université Paris-Dauphine, PSL Research University, CNRS, LAMSADE, 75016 Paris, France.*

^5^Université de Liège: *Laboratory of Pharmacognosy, Center of Interdisciplinary Research on Medicines (CIRM), University of Liège, Liège, Belgium.*

^6^Universiti Malaya: *Department of Chemistry, Faculty of Science, Universiti Malaya, 50603 Kuala Lumpur, Malaysia.*

^7^Instituto de BioEconomia: *El Batan, Quito, 170135, Ecuador.*

^8^Université des Sciences de la Santé, *Département Science Fondam.entale, Service Chimie-Biochimie, Owendo, Gabon.*

^9^University of Lagos: *Department of Pharmaceutical Chemistry, Faculty of Pharmacy, College of Medicine, Idiaraba Campus, Surulere, Lagos, Nigeria.*

^10^Leiden University: *Natural Products Laboratory, Institute of Biology, Leiden University, Sylviusweg 72, 2333 BE Leiden, the Netherlands.*

^11^Université de Tours: *EA2106 Biomolécules et Biotechnologies Végétales, Université de Tours, 31 avenue Monge, 37200 Tours, France.*

^12^Université Paris-Cité*: UMR 8038 CiTCoM, Faculté de santé, Université Paris Cité, CNRS, 75006 Paris, France.*

^13^Düsseldorf University of Applied Sciences: *Centre for Digitalisation and Digitality, Düsseldorf University of Applied Sciences, 40476 Düsseldorf, Germany.*

^14^Wageningen University & Research: *Bioinformatics Group, Wageningen University, 6708PB Wageningen, the Netherlands.*

^15^University of Johannesburg: *Department of Biochemistry, University of Johannesburg, 2006 Johannesburg, South Africa.*

^✝^ these authors contributed equally to the work.

* Corresponding authors: [pierre.le-pogam-alluard@universite-paris-saclay.fr](mailto:pierre.le-pogam-alluard@universite-paris-saclay.fr) and [mehdi.beniddir@universite-paris-saclay.fr](mailto:mehdi.beniddir@universite-paris-saclay.fr)

Summary

Fig. S1 Heatmap disclosing the modified cosine score pairwise similarity matrix for the whole, non-ordered MIADB - Monoterpene Indole Alkaloids DataBase skeletons

Fig. S2 Heatmap disclosing the modified cosine score pairwise similarity matrix for the whole, ordered MIADB

Fig. S3 Whole-ordered MIADB heatmap filtered by a minimum modified cosine score threshold of 0.9

Fig. S4 Heatmap displaying the Spec2vec score pairwise similarity matrix for the whole, ordered MIADB

Fig. S5 Whole-ordered MIADB heatmap filtered by a minimum Spec2Vec score threshold of 0.9

Fig. S6 Heatmap displaying the MS2DeepScore pairwise similarity matrix for the whole, ordered MIADB

Fig. S7 Heatmap disclosing the cosine score pairwise similarity matrix for the whole, ordered MIADB

Fig. S8 Expanded MIADB modified cosine heatmap of the indoloquinolizidine-containing MIAs

Fig. S9 Expanded MIADB modified cosine heatmap of the indoloquinolizidine-containing MIAs retaining only modified cosine scores over 0.9

Fig. S10 MS/MS similarities of ochropposinine (A-ring dimethoxylated corynantheane spirooxindole) as evidenced by the modified cosine heatmap score across the indoloquinolizidine-containing MIA region shown in Fig. 3. Its MS/MS spectrum appears to be close to that of similarly substituted MIAs, irrespective of their skeletons.

Fig. S11 Expanded MIADB modified cosine heatmap of the ajmalicine spirooxindole representatives and corynantheane spirooxindole representatives retaining only modified cosine scores over 0.9

Table S1 Monoterpene Indole Alkaloid Skeletons and their respective simplified SMILES and SMARTS-encoded chemical structures

Fig. S12 Monoterpene Indole Alkaloids skeletons included in the MIADB

Fig. S13 Number of spectra per skeleton included in the MIADB

Fig. S14 Additional Monoterpene Indole Alkaloids skeletons

Table S2 Plant extracts selected for analysis

Table S3 MassQL queries using product ions and neutral losses

Table S4 GNPS-annotation based accuracy and recall of the MassQL queries on the 75 plant dataset

Table S5 Plant source disclosing the most elevated signal intensity for each feature tentatively identified by MassQL as an ajmalicine spirooxindole

Table S6 Most common plant sources genera for ions with the highest signal intensity for features tentatively identified by MassQL as an ajmalicine spirooxindole

Table S7 Plant source disclosing the most elevated signal intensity for each feature tentatively identified by MassQL as a corynantheane spirooxindole

Table S8 Most common plant sources genera for ions with the highest signal intensity for features tentatively identified by MassQL as a corynantheane spirooxindole

Table S9 Plant source disclosing the most elevated signal intensity for each feature tentatively identified by MassQL as a “corynantheane spirooxindole or ajmalicine spirooxindole”

Table S10 Plant sources genera for ions with the highest signal intensity for features tentatively identified by MassQL as “corynantheane spirooxindole or ajmalicine spirooxindole”

Fig. S15 Genus repartition of the ions annotated as ajmalicine spirooxindoles by the MassQL query in the 75 plant extracts dataset

Fig. S16 Genus repartition of the ions annotated as corynantheane spirooxindoles by the MassQL query in the 75 plant extracts dataset

Fig. S17 Genus repartition of the ions annotated as ajmalicine spirooxindoles or corynantheane spirooxindoles by the MassQL query in the 75 plant extracts dataset

Fig. S18 Example of a cluster containing ions annotated as ajmalicine spirooxindoles or Corynantheane spirooxindoles by the GNPS, the MassQL query in the 75 plant extracts dataset, and/or TIMA-R tool on the 75 plants dataset

Fig. S19 Global MIADB network with skeletons-annotated features

Fig. S20 Global MIADB network: zoom on the indoloquinolizidine-containing MIA cluster

Fig. S21 Global MIADB network: zoom on the ajmalicine spirooxindole cluster


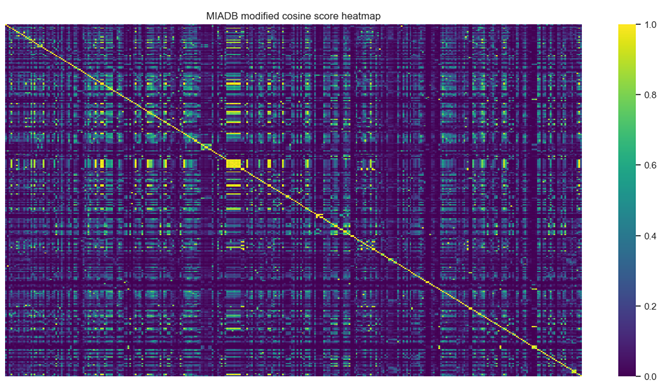


Fig. S1 Heatmap disclosing the modified cosine score pairwise similarity matrix for the whole, non-ordered MIADB- Monoterpene Indole Alkaloids DataBase skeletons


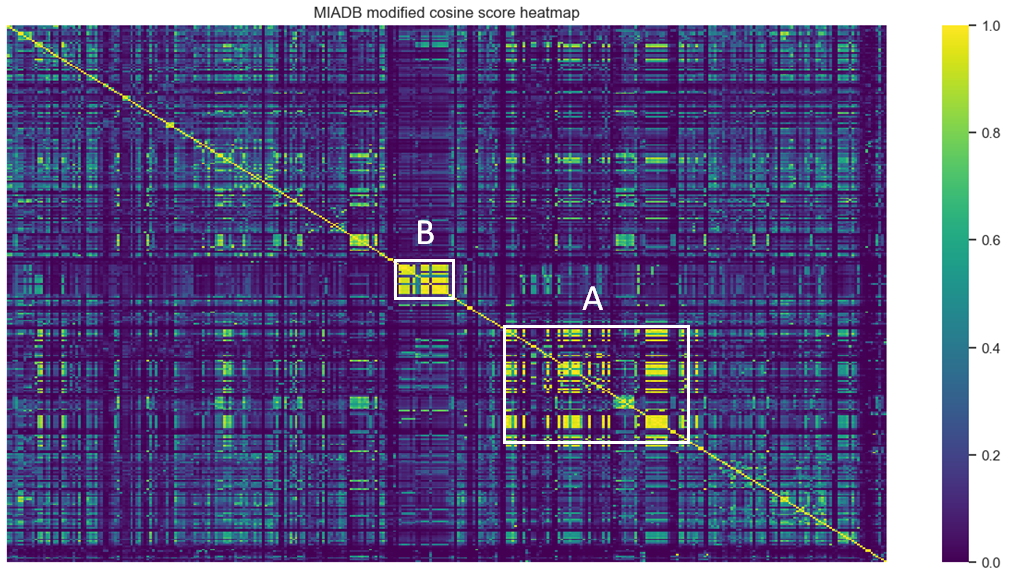
Fig. S2 Heatmap disclosing the modified cosine score pairwise similarity matrix for the whole MIADB. Axes are ordered according to the Tanimoto-based assessment of skeleton similarity. A and B refer to regions used to discuss the relationship between structure and MS/MS similarity later on.


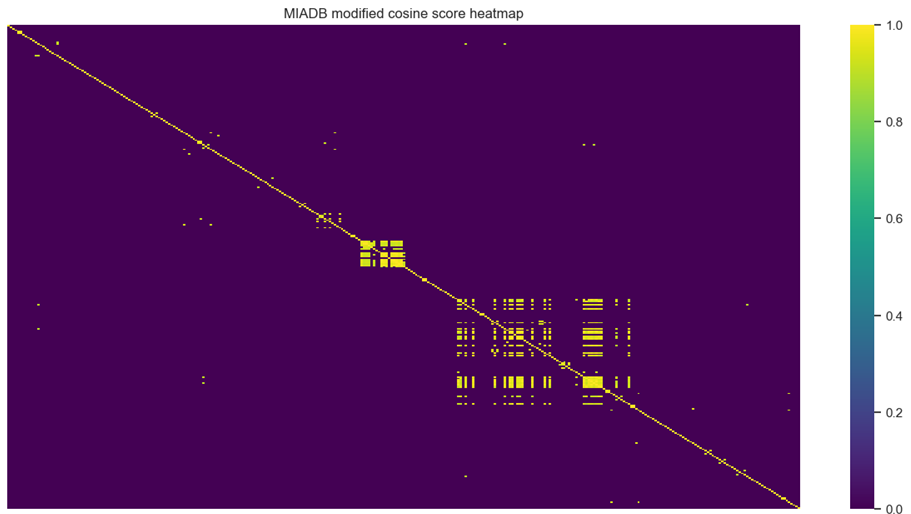
Fig. S3 Whole-ordered MIADB heatmap filtered by a minimum modified cosine score threshold of 0.9


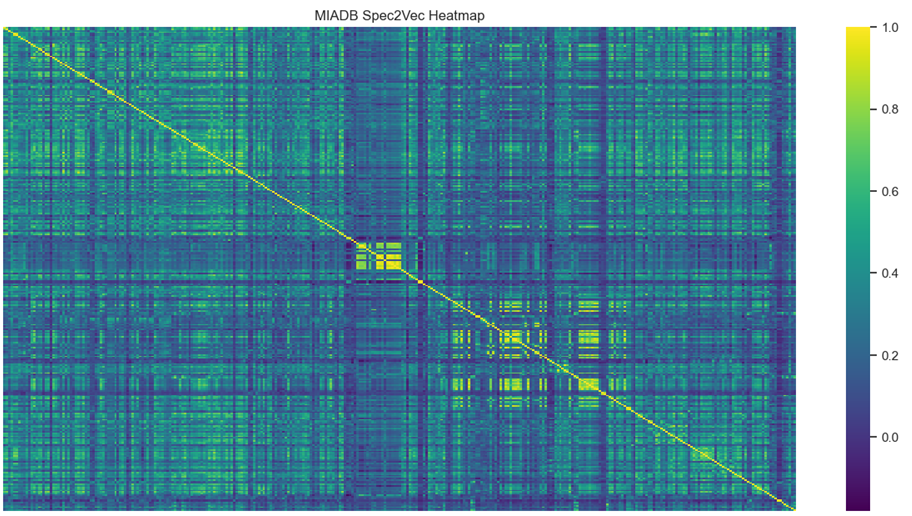
Fig. S4 Heatmap displaying the Spec2vec score pairwise similarity matrix for the whole, ordered MIADB


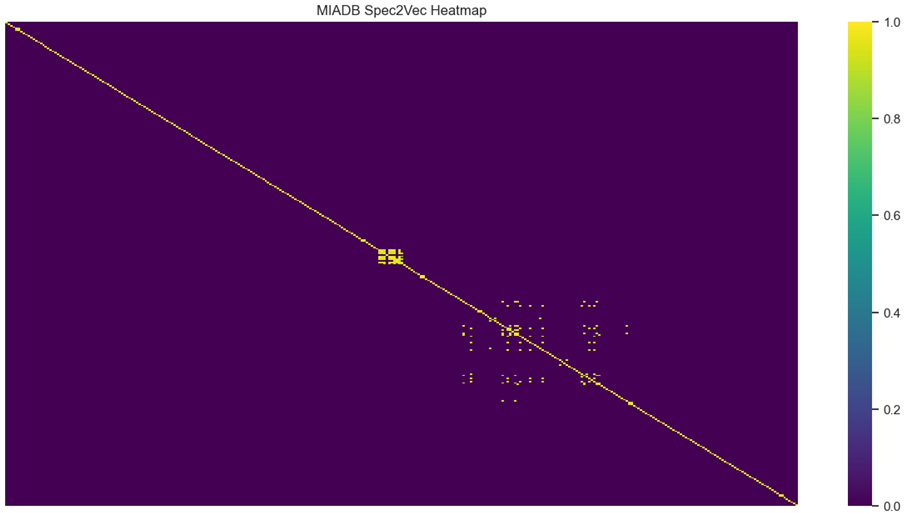
Fig. S5 Whole-ordered MIADB heatmap filtered by a minimum Spec2Vec score threshold of 0.9


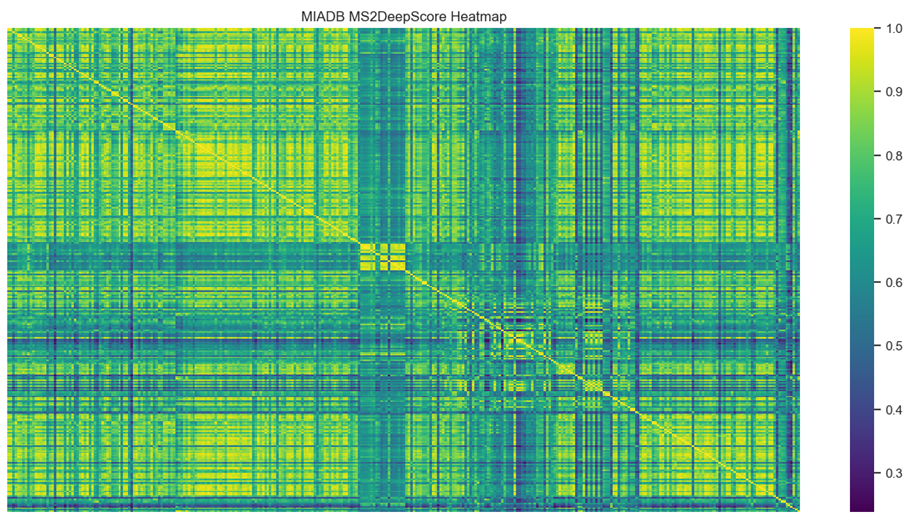
Fig. S6 Heatmap displaying the MS2DeepScore pairwise similarity matrix for the whole, ordered MIADB


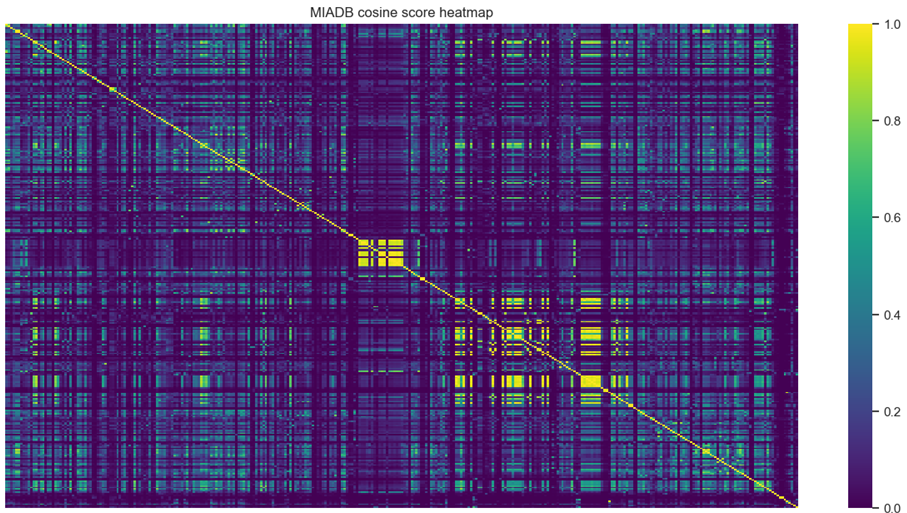
Fig. S7 Heatmap disclosing the classic cosine score pairwise similarity matrix for the whole, ordered MIADB


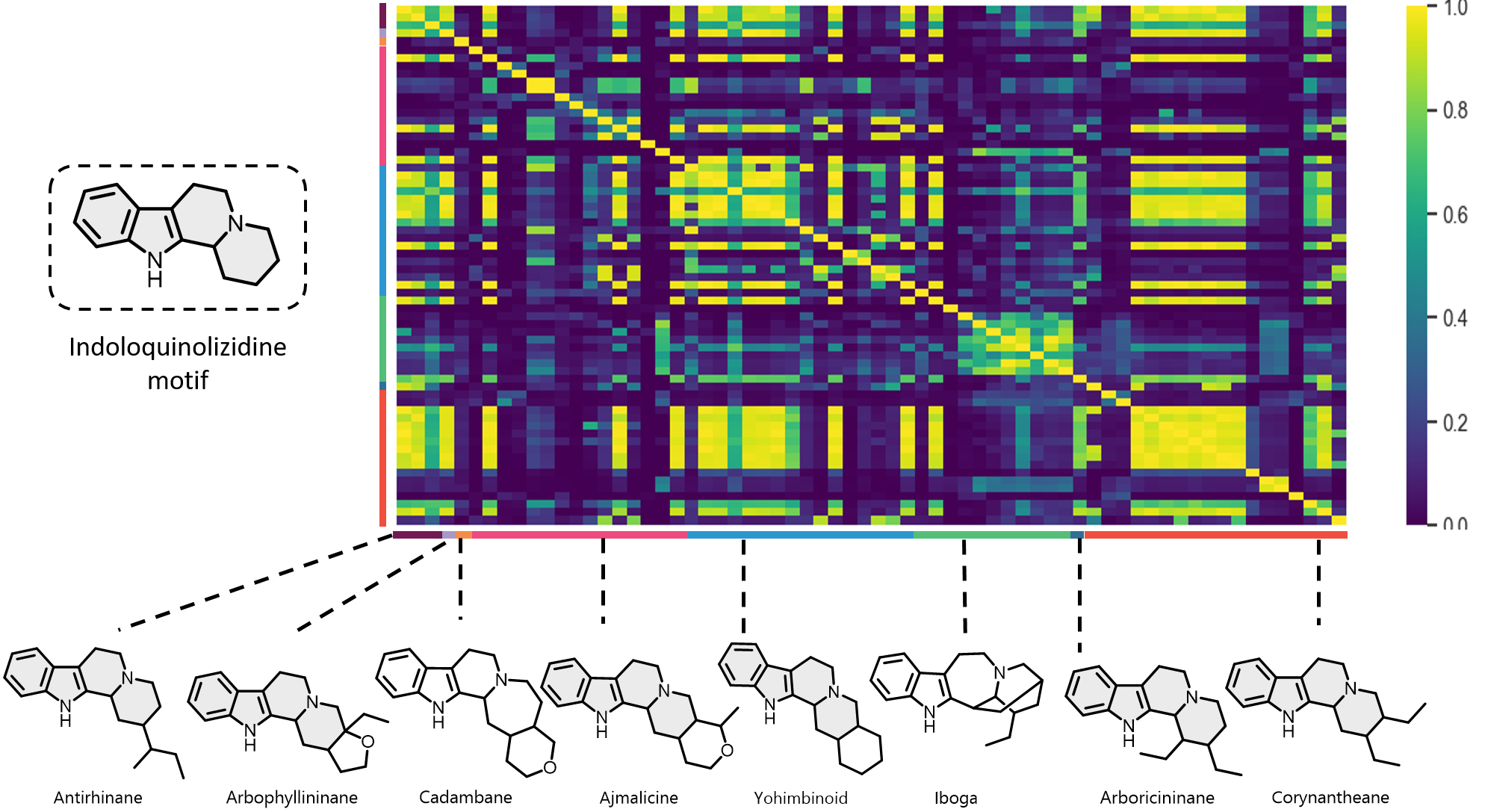


Fig. S8 Expanded MIADB modified cosine heatmap of the indoloquinolizidine-containing MIAs


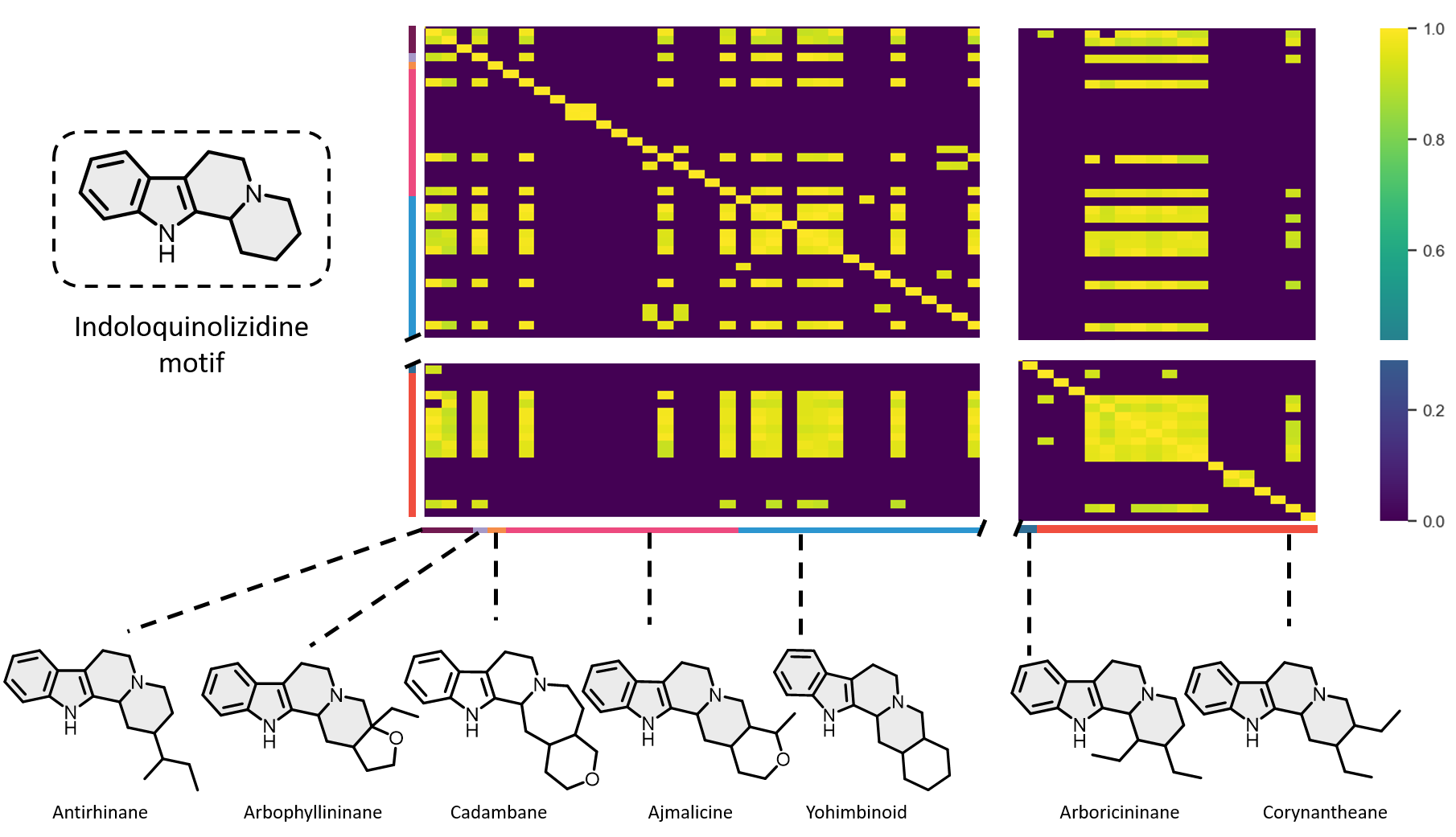


Fig. S9 Expanded MIADB modified cosine heatmap of the indoloquinolizidine-containing MIAs retaining only modified cosine scores over 0.9 (others appear to be null)


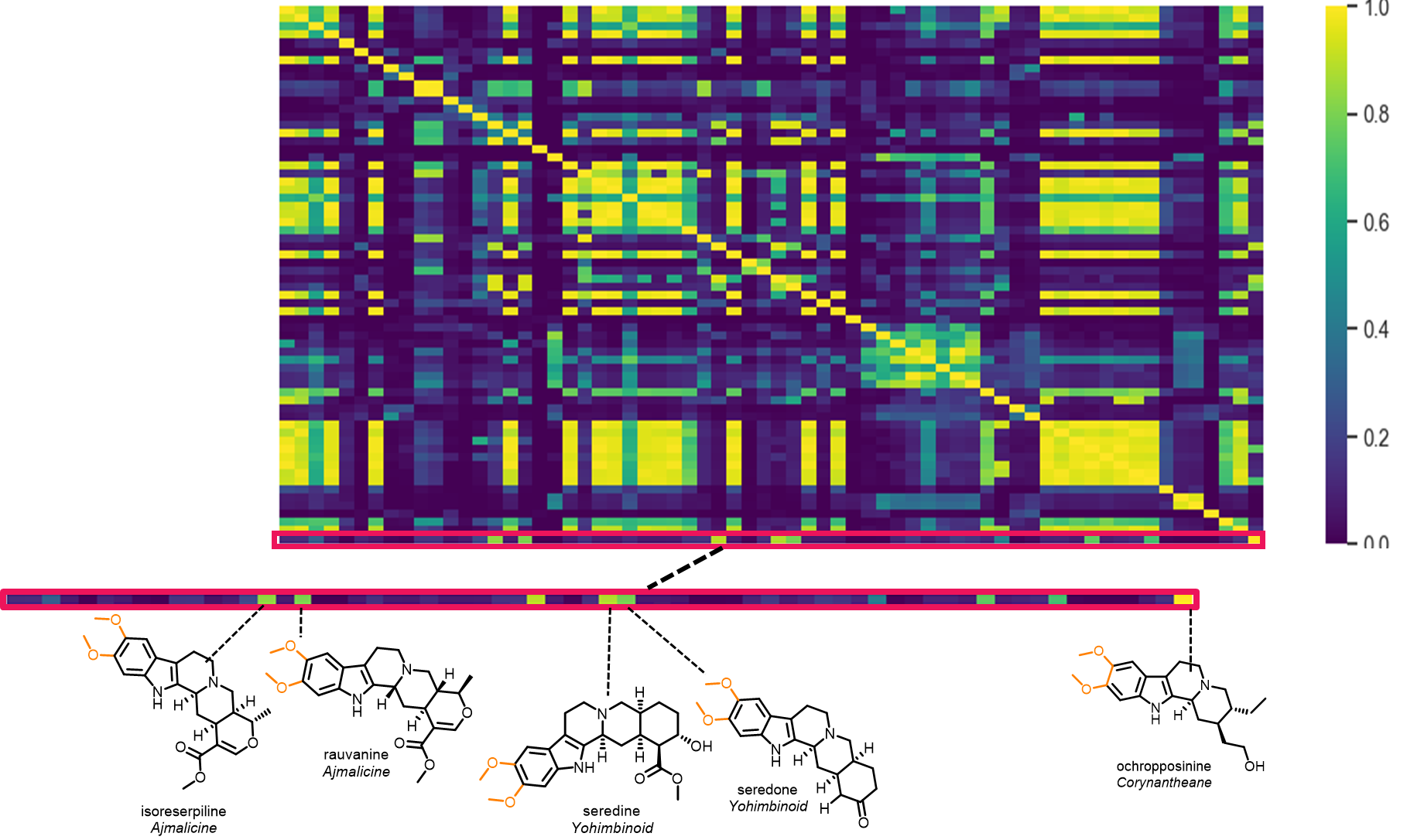


Fig. S10 MS/MS similarities of ochropposinine (A-ring dimethoxylated corynantheane spirooxindole) as evidenced by the modified cosine heatmap score across the indoloquinolizidine-containing MIA region shown in Fig. 3. Its MS/MS spectrum appears to be close to that of similarly substituted MIAs, irrespective of their skeletons.


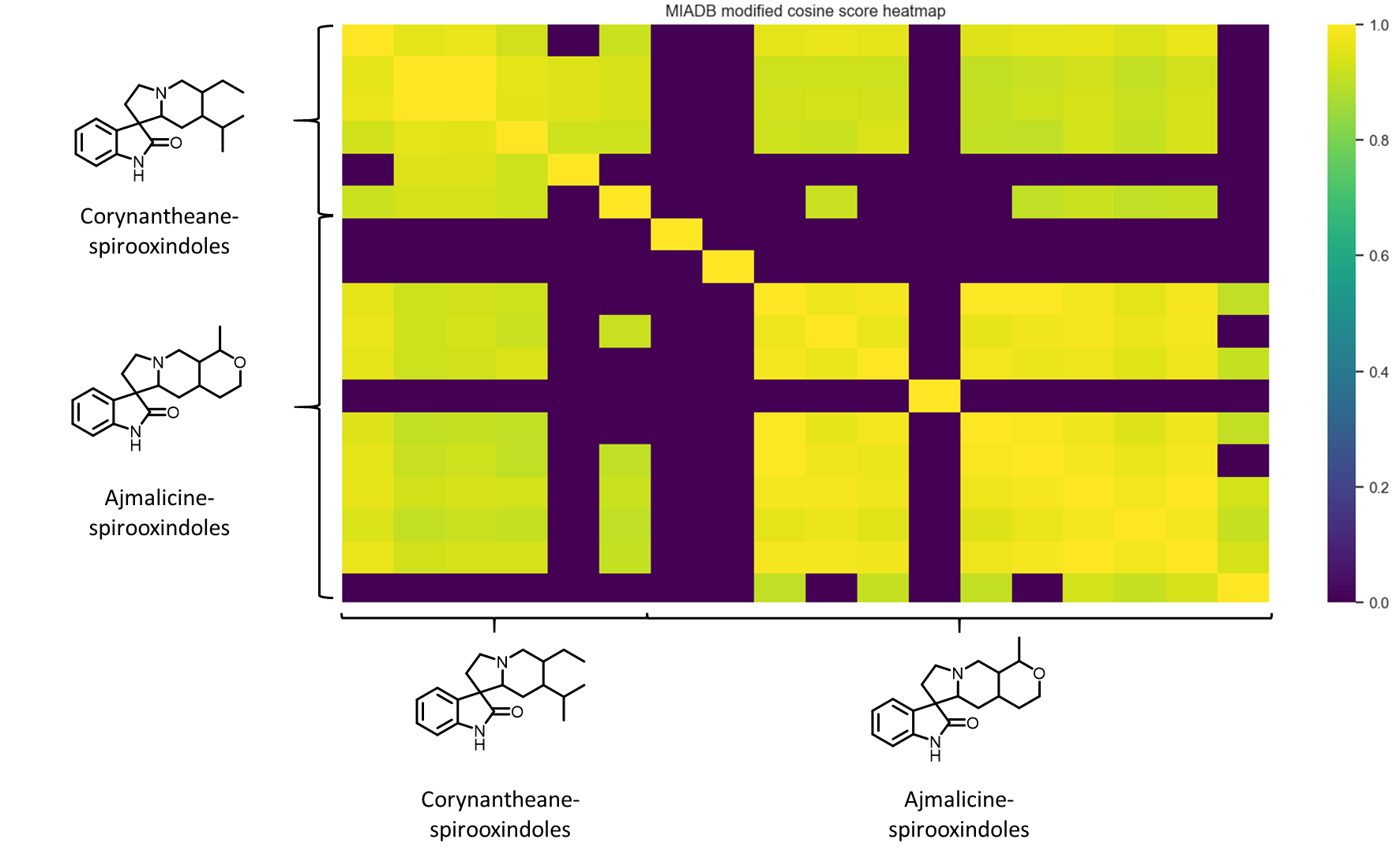
Fig. S11 Expanded MIADB modified cosine heatmap of the ajmalicine spirooxindole representatives and corynantheane spirooxindole representatives retaining only modified cosine scores over 0.9 (others appear to be null)

Table S1 Monoterpene Indole Alkaloid Skeletons and their respective simplified SMILES and SMARTS-encoded chemical structures

| Skeleton | SMILES | SMARTS | Skeleton_qid |
| --- | --- | --- | --- |
| Ajmalicine | CC1OCCC2CC3C4=C(CCN3CC12)C5=C(C=CC=C5)N4 | [#6]-[#6]1-[#8]-[#6]-[#6]-[#6]2-[#6]-[#6]3-[#6]4:[#6](-[#6]-[#6]-[#7]-3-[#6]-[#6]-1-2):[#6]1:[#6](:[#6]:[#6]:[#6]:[#6]:1):[#7H]:4 | Q131287066 |
| Ajmalicine spirooxindole | CC1C2CN(C3CC2CCO1)CCC43C5=C(NC4=O)C=CC=C5 | [#6]-[#6]1-[#6]2-[#6]-[#7]3-[#6](-[#6]-[#6]-2-[#6]-[#6]-[#8]-1)-[#6]1(-[#6]-[#6]-3)-[#6]2:[#6](-[#7]-[#6]-1=[#8]):[#6]:[#6]:[#6]:[#6]:2 | Q131287129 |
| Ajmaline | CCC1CN2C3C(C45CC2C(C5)C1C3)NC6=C4C=CC=C6 | [#6]-[#6]-[#6]1-[#6]-[#7]2-[#6]3-[#6]4-[#6]5(-[#6]-[#6]-2-[#6](-[#6]-5)-[#6]-1-[#6]-3)-[#6]1:[#6](-[#7]-4):[#6]:[#6]:[#6]:[#6]:1 | Q131287132 |
| Akagerane | CCCC1CC2NCCC3=C2N(CC1)C4=C3C=CC=C4 | [#6]-[#6]-[#6]-[#6]1-[#6]-[#6]2-[#7]-[#6]-[#6]-[#6]3:[#6]-2:[#7](-[#6]-[#6]-1):[#6]1:[#6]:3:[#6]:[#6]:[#6]:[#6]:1 | Q131287139 |
| Correantane | CCC1CC2C(N(C3=CC=CC=C34)CC1C)=C4CCN2 | [#6]-[#6]-[#6]1-[#6]-[#6]2-[#6]3:[#7](:[#6]4:[#6]:[#6]:[#6]:[#6]:[#6]:4:[#6]:3-[#6]-[#6]-[#7]-2)-[#6]-[#6]-1-[#6] | Q131287141 |
| Akuammicine | CCC1C2CC(C34C(N(CC4)C1)C2)NC5=C3C=CC=C5 | [#6]-[#6]-[#6]1-[#6]2-[#6]-[#6]3-[#6]4(-[#6](-[#7](-[#6]-[#6]-4)-[#6]-1)-[#6]-2)-[#6]1:[#6](-[#7]-3):[#6]:[#6]:[#6]:[#6]:1 | Q131287144 |
| Akuammiline | CCC1CN2CCC34C(C=CC=C5)=C5NC3C2CC1C4 | [#6]-[#6]-[#6]1-[#6]-[#7]2-[#6]-[#6]-[#6]34-[#6]5:[#6]:[#6]:[#6]:[#6]:[#6]:5-[#7]-[#6]-3-[#6]-2-[#6]-[#6]-1-[#6]-4 | Q131287147 |
| Seco-akuammiline | CCC1CNC2CC1CC3(CCO4)C24NC5=C3C=CC=C5 | [#6]-[#6]-[#6]1-[#6]-[#7]-[#6]2-[#6]-[#6]-1-[#6]-[#6]13-[#6]-[#6]-[#8]-[#6]-2-1-[#7]-[#6]1:[#6]-3:[#6]:[#6]:[#6]:[#6]:1 | Q131287150 |
| Arbornane | CCC1CN2CCC3=C4N(CCC1C24)C5=CC=CC=C35 | [#6]-[#6]-[#6]1-[#6]-[#7]2-[#6]-[#6]-[#6]3:[#6]4:[#7](-[#6]-[#6]-[#6]-1-[#6]-2-4):[#6]1:[#6]:[#6]:[#6]:[#6]:[#6]:3:1 | Q131287152 |
| Aspidofractane | C1(NC23CCC4(C5C36CCN5CCC4)CC2)=C6C=CC=C1 | [#6]12-[#7]-[#6]34-[#6]-[#6]-[#6]5(-[#6]6-[#6]-3(-[#6]-[#6]-[#7]-6-[#6]-[#6]-[#6]-5)-[#6]:1:[#6]:[#6]:[#6]:[#6]:2)-[#6]-[#6]-4 | Q131287154 |
| Aspidosperma | CCC12CCC(C34C1N(CCC2)CC4)NC5=C3C=CC=C5 | [#6]-[#6]-[#6]12-[#6]-[#6]-[#6]3-[#6]4(-[#6]-1-[#7](-[#6]-[#6]-[#6]-2)-[#6]-[#6]-4)-[#6]1:[#6](-[#7]-3):[#6]:[#6]:[#6]:[#6]:1 | Q131287155 |
| Cadambane | C1(NC2=C3CCN4CCC(COCC5)C5CC24)=C3C=CC=C1 | [#6]12:[#7H]:[#6]3:[#6](-[#6]-[#6]-[#7]4-[#6]-[#6]-[#6]5-[#6]-[#8]-[#6]-[#6]-[#6]-5-[#6]-[#6]-3-4):[#6]:1:[#6]:[#6]:[#6]:[#6]:2 | Q131287157 |
| Camptothecine | O=C1CC(C=C2N3CC4=CC5=C(N=C24)C=CC=C5)=C(CO1)C3=O | [#8]=[#6]1-[#6]-[#6]2:[#6]:[#6]3:[#7](-[#6]-[#6]4:[#6]:[#6]5:[#6](:[#7]:[#6]:4-3):[#6]:[#6]:[#6]:[#6]:5):[#6](:[#6]:2-[#6]-[#8]-1)=[#8] | Q131287158 |
| Seco-  camptothecine | CC1=C(C=C2C3=NC4=C(C=C3CN2C1=O)C=CC=C4)C(CC)C | [#6]-[#6]1:[#6](:[#6]:[#6]2-[#6]3:[#7]:[#6]4:[#6](:[#6]:[#6]:3-[#6]-[#7]:2:[#6]:1=[#8]):[#6]:[#6]:[#6]:[#6]:4)-[#6](-[#6]-[#6])-[#6] | Q131287159 |
| Chanofruticosinate | O=C(C1)C2CN(CCC3)C4C13CCC5C42C(C=CC=C6)=C6N5 | [#8]=[#6]1-[#6]-[#6]23-[#6]-[#6]-[#6]-[#7]4-[#6]-[#6]-1-[#6]1(-[#6]-4-2)-[#6](-[#6]-[#6]-3)-[#7]-[#6]2:[#6]-1:[#6]:[#6]:[#6]:[#6]:2 | Q131287160 |
| Chippiine | CCC1CC2CC(C1NCC3)C4=C3C5=C(C=CC=C5)N4C2 | [#6]-[#6]-[#6]1-[#6]-[#6]2-[#6]-[#6]3-[#6]-1-[#7]-[#6]-[#6]-[#6]1:[#6]-3:[#7](:[#6]3:[#6]:1:[#6]:[#6]:[#6]:[#6]:3)-[#6]-2 | Q131287161 |
| Cinchona | CCC1C(CCN2C1)CC2CC3=CC=NC4=C3C=CC=C4 | [#6]-[#6]-[#6]1-[#6]2-[#6]-[#6]-[#7](-[#6]-1)-[#6](-[#6]-2)-[#6]-[#6]1:[#6]:[#6]:[#7]:[#6]2:[#6]:1:[#6]:[#6]:[#6]:[#6]:2 | Q131287162 |
| Indolocinchona | CCC1CN2C(CC1CC2)C3=C(C4=CC=CC=C4N3)CC | [#6]-[#6]-[#6]1-[#6]-[#7]2-[#6](-[#6]-[#6]-1-[#6]-[#6]-2)-[#6]1:[#6](:[#6]2:[#6]:[#6]:[#6]:[#6]:[#6]:2:[#7H]:1)-[#6]-[#6] | Q131287163 |
| Condylocarpane | CCC1C2CC(C34C1N(CC4)CC2)NC5=C3C=CC=C5 | [#6]-[#6]-[#6]1-[#6]2-[#6]-[#6]3-[#6]4(-[#6]-1-[#7](-[#6]-[#6]-4)-[#6]-[#6]-2)-[#6]1:[#6](-[#7]-3):[#6]:[#6]:[#6]:[#6]:1 | Q131287164 |
| Goniomane | C12=CC=CC=C1C3(CCN4CCC5CC3)C4C5CN2 | [#6]12:[#6]:[#6]:[#6]:[#6]:[#6]:1-[#6]13-[#6]-[#6]-[#7]4-[#6]-[#6]-[#6](-[#6]-[#6]-1)-[#6](-[#6]-3-4)-[#6]-[#7]-2 | Q131287165 |
| Corynantheane | CCC1C(CC)CN(C2C1)CCC3=C2NC4=CC=CC=C43 | [#6]-[#6]-[#6]1-[#6](-[#6]-[#6])-[#6]-[#7]2-[#6](-[#6]-1)-[#6]1:[#6](-[#6]-[#6]-2):[#6]2:[#6](:[#7H]:1):[#6]:[#6]:[#6]:[#6]:2 | Q131287166 |
| Corynantheane spirooxindole | O=C1NC2=CC=CC=C2C13C4CC(C(CC)CN4CC3)C(C)C | [#8]=[#6]1-[#7]-[#6]2:[#6]:[#6]:[#6]:[#6]:[#6]:2-[#6]-12-[#6]1-[#6]-[#6](-[#6](-[#6]-[#6])-[#6]-[#7]-1-[#6]-[#6]-2)-[#6](-[#6])-[#6] | Q131287168 |
| Eburna | CCC12CCCN3C1C(N(CC2)C4=C5C=CC=C4)=C5CC3 | [#6]-[#6]-[#6]12-[#6]-[#6]-[#6]-[#7]3-[#6]-1-[#6]1:[#7](-[#6]-[#6]-2):[#6]2:[#6](:[#6]:[#6]:[#6]:[#6]:2):[#6]:1-[#6]-[#6]-3 | Q131287169 |
| Ervatamia | CCC1CNCC2C1CCC3=C(C4=C(N3)C=CC=C4)C2 | [#6]-[#6]-[#6]1-[#6]-[#7]-[#6]-[#6]2-[#6]-1-[#6]-[#6]-[#6]1:[#6](:[#6]3:[#6](:[#7H]:1):[#6]:[#6]:[#6]:[#6]:3)-[#6]-2 | Q131287170 |
| Gelseminane | CC(C1CCC23C([N]C4=CC=CC=C34)=O)C5C2C1(CC)CN5 | [#6]-[#6]1-[#6]2-[#6]-[#6]-[#6]3(-[#6](-[#7]-[#6]4:[#6]:[#6]:[#6]:[#6]:[#6]:4-3)=[#8])-[#6]3-[#6]-1-[#7]-[#6]-[#6]-2-3-[#6]-[#6] | Q131287172 |
| Gelsedane | CCC1NC2CC3(CCC1C2)C(NC4=CC=CC=C34)=O | [#6]-[#6]-[#6]1-[#7]-[#6]2-[#6]-[#6]3(-[#6]-[#6]-[#6]-1-[#6]-2)-[#6](-[#7]-[#6]1:[#6]:[#6]:[#6]:[#6]:[#6]:1-3)=[#8] | Q131287173 |
| Gelseleginane | CC[C@@]1(C)[N][C@@H]2C[C@]3(CCC1[C@@H]2C)C4=CC=CC=C4NC3=O | [#6]-[#6]-[#6@@]1(-[#6])-[#7]-[#6@@H]2-[#6]-[#6@]3(-[#6]-[#6]-[#6]-1-[#6@@H]-2-[#6])-[#6]1:[#6]:[#6]:[#6]:[#6]:[#6]:1-[#7]-[#6]-3=[#8] | Q131287174 |
| Isohumanteninane | O=C1NC(C=CC=C2)=C2C1(C3)CCC4C(C)C(C)NC3C4C | [#8]=[#6]1-[#7]-[#6]2:[#6]:[#6]:[#6]:[#6]:[#6]:2-[#6]-12-[#6]-[#6]1-[#7]-[#6](-[#6](-[#6](-[#6]-[#6]-2)-[#6]-1-[#6])-[#6])-[#6] | Q131287176 |
| Iboga | CCC1C2N3CC(CC2C4=C(CC3)C5=C(N4)C=CC=C5)C1 | [#6]-[#6]-[#6]1-[#6]2-[#7]3-[#6]-[#6](-[#6]-[#6]-2-[#6]2:[#6](-[#6]-[#6]-3):[#6]3:[#6](:[#7H]:2):[#6]:[#6]:[#6]:[#6]:3)-[#6]-1 | Q131287177 |
| Tabertingginane | C1(NC2=C3CCN4CC5CC42CCC5)=C3C=CC=C1 | [#6]12:[#7H]:[#6]3:[#6](-[#6]-[#6]-[#7]4-[#6]-[#6]5-[#6]-[#6]-3-4-[#6]-[#6]-[#6]-5):[#6]:1:[#6]:[#6]:[#6]:[#6]:2 | Q131287179 |
| Andranginane | C12=CC=CC=C1C(CCN3C4C5CCCC4CCC3)=C5N2 | [#6]12:[#6]:[#6]:[#6]:[#6]:[#6]:1:[#6]1-[#6]-[#6]-[#7]3-[#6]4-[#6](-[#6]-[#6]-[#6]-[#6]-4-[#6]-[#6]-[#6]-3)-[#6]:1:[#7H]:2 | Q131287180 |
| Conolutinane | CCC1CC2CCC34N(C5=CC=CC=C5C3CCN4C1)C2 | [#6]-[#6]-[#6]1-[#6]-[#6]2-[#6]-[#6]-[#6]34-[#7](-[#6]5:[#6]:[#6]:[#6]:[#6]:[#6]:5-[#6]-3-[#6]-[#6]-[#7]-4-[#6]-1)-[#6]-2 | Q131287181 |
| Lirofolane | C12CCC(C3C2)N(CN4C3=CC5=C4C=CC=C5)C1 | [#6]12-[#6]-[#6]-[#6]3-[#6](-[#6]-1)-[#6]1:[#7](-[#6]-[#7]-3-[#6]-2):[#6]2:[#6](:[#6]:1):[#6]:[#6]:[#6]:[#6]:2 | Q131287182 |
| Kopsane | C1(NC23CCC45CC2CC6C37C4N(CCC5)C6)=C7C=CC=C1 | [#6]12-[#7]-[#6]34-[#6]-[#6]-[#6]56-[#6]-[#6]-3-[#6]-[#6]3-[#6]-4(-[#6]-5-[#7](-[#6]-[#6]-[#6]-6)-[#6]-3)-[#6]:1:[#6]:[#6]:[#6]:[#6]:2 | Q131287184 |
| Lapidilectine | C1(NC23C4CCN5CCCC5(CC3)CC2)=C4C=CC=C1 | [#6]12-[#7]-[#6]34-[#6](-[#6]-[#6]-[#7]5-[#6]-[#6]-[#6]-[#6]-5(-[#6]-[#6]-3)-[#6]-[#6]-4)-[#6]:1:[#6]:[#6]:[#6]:[#6]:2 | Q131287185 |
| Leuconoxane | CCC12CCCN3CCC4C5=CC=CC=C5N(CCC2)C143 | [#6]-[#6]-[#6]12-[#6]-[#6]-[#6]-[#7]3-[#6]-[#6]-[#6]4-[#6]5:[#6]:[#6]:[#6]:[#6]:[#6]:5-[#7](-[#6]-[#6]-[#6]-1)-[#6]-2-3-4 | Q131287187 |
| Macroline | CC1C2NC(CC1C(C)CC)C3=C(C4=C(C=CC=C4)N3)C2 | [#6]-[#6]1-[#6]2-[#7]-[#6](-[#6]-[#6]-1-[#6](-[#6])-[#6]-[#6])-[#6]1:[#6](:[#6]3:[#6](:[#6]:[#6]:[#6]:[#6]:3):[#7H]:1)-[#6]-2 | Q131287188 |
| Malindane | CC1C2=C(CC3N1CCC4=C3NC5=CC=CC=C45)C=CN=C2 | [#6]-[#6]1-[#6]2:[#6](-[#6]-[#6]3-[#7]-1-[#6]-[#6]-[#6]1:[#6]-3:[#7H]:[#6]3:[#6]:[#6]:[#6]:[#6]:[#6]:1:3):[#6]:[#6]:[#7]:[#6]:2 | Q131287189 |
| Melodinus | C1(NCC2C34C5C(CCCN5CC4)C2)=C3C=CC=C1 | [#6]12-[#7]-[#6]-[#6]3-[#6]4(-[#6]5-[#6](-[#6]-[#6]-[#6]-[#7]-5-[#6]-[#6]-4)-[#6]-3)-[#6]:1:[#6]:[#6]:[#6]:[#6]:2 | Q131287190 |
| Mersinlonginane | C1(NC2NCCC34C2CCCC4)=C3C=CC=C1 | [#6]12-[#7]-[#6]3-[#7]-[#6]-[#6]-[#6]4(-[#6]-3-[#6]-[#6]-[#6]-[#6]-4)-[#6]:1:[#6]:[#6]:[#6]:[#6]:2 | Q131287191 |
| Melonine | CCC12CCC(NC3=C4C=CC=C3)(C5CC2)C4CCN5C1 | [#6]-[#6]-[#6]12-[#6]-[#6]-[#6]34-[#7]-[#6]5:[#6](:[#6]:[#6]:[#6]:[#6]:5)-[#6]-3-[#6]-[#6]-[#7](-[#6]-4-[#6]-[#6]-1)-[#6]-2 | Q131287193 |
| Nacycline | CCC1C2NCCC1CC3=NC=CC4=C3N2C5=C4C=CC=C5 | [#6]-[#6]-[#6]1-[#6]2-[#7]-[#6]-[#6]-[#6]-1-[#6]-[#6]1:[#7]:[#6]:[#6]:[#6]3:[#6]:1:[#7]-2:[#6]1:[#6]:3:[#6]:[#6]:[#6]:[#6]:1 | Q131287195 |
| Pandoline | CCC(C1)CN2C3C1CCC(C43CC2)NC5=C4C=CC=C5 | [#6]-[#6]-[#6]1-[#6]-[#6]2-[#6]3-[#7](-[#6]-1)-[#6]-[#6]-[#6]-31-[#6](-[#6]-[#6]-2)-[#7]-[#6]2:[#6]-1:[#6]:[#6]:[#6]:[#6]:2 | Q131287196 |
| Arboridinane | C12=NC3=CC=CC=C3C14CCN(CC2C5)CC5C4 | [#6]12=[#7]-[#6]3:[#6]:[#6]:[#6]:[#6]:[#6]:3-[#6]-13-[#6]-[#6]-[#7]1-[#6]-[#6]-2-[#6]-[#6](-[#6]-1)-[#6]-3 | Q131287197 |
| Arborisidinane | C12C3CCN1CCC4(C5=CC=CC=C5N=C24)CC3 | [#6]12-[#6]3-[#6]-[#6]-[#7]-1-[#6]-[#6]-[#6]1(-[#6]4:[#6]:[#6]:[#6]:[#6]:[#6]:4-[#7]=[#6]-2-1)-[#6]-[#6]-3 | Q131287198 |
| Pleiocarpamane | N12CCC(C3C2C4)C5=C(C=CC=C5)N3CC4CC1 | [#7]12-[#6]-[#6]-[#6]3-[#6]4-[#6]-1-[#6]-[#6](-[#6]-[#7]-4-[#6]1:[#6]-3:[#6]:[#6]:[#6]:[#6]:1)-[#6]-[#6]-2 | Q131287199 |
| Pyridocarbazole | CC1=C2C=CN=CC2=CC3=C1NC4=C3C=CC=C4 | [#6]-[#6]1:[#6]2:[#6]:[#6]:[#7]:[#6]:[#6]:2:[#6]:[#6]2:[#6]:1:[#7H]:[#6]1:[#6]:2:[#6]:[#6]:[#6]:[#6]:1 | Q131287200 |
| Quebrachamine | CCC12CN(CCC3=C(CC2)NC4=C3C=CC=C4)CCC1 | [#6]-[#6]-[#6]12-[#6]-[#7](-[#6]-[#6]-[#6]3:[#6](-[#6]-[#6]-1):[#7H]:[#6]1:[#6]:3:[#6]:[#6]:[#6]:[#6]:1)-[#6]-[#6]-[#6]-2 | Q131287201 |
| Rhazidane | C12=CC=CC=C1C3CC[N+]45CCCC(C5)CCC43N2 | [#6]12:[#6]:[#6]:[#6]:[#6]:[#6]:1-[#6]1-[#6]-[#6]-[#7+]34-[#6]-[#6]-[#6]-[#6](-[#6]-3)-[#6]-[#6]-[#6]-1-4-[#7]-2 | Q131287202 |
| Rhazinilam | O=C(CCC1C2C3=CCN2CCC1)NC4=C3C=CC=C4 | [#8]=[#6]1-[#6]-[#6]-[#6]2-[#6]3-[#6](=[#6]-[#6]-[#7]-3-[#6]-[#6]-[#6]-2)-[#6]2:[#6](-[#7]-1):[#6]:[#6]:[#6]:[#6]:2 | Q131287204 |
| Sarpagine | C1(NC2=C3CC4N5C2CC(CC5)C4)=C3C=CC=C1 | [#6]12:[#7H]:[#6]3:[#6](-[#6]-[#6]4-[#7]5-[#6]-3-[#6]-[#6](-[#6]-[#6]-5)-[#6]-4):[#6]:1:[#6]:[#6]:[#6]:[#6]:2 | Q131287205 |
| Schizozygane | C12CCN3CCCC4(CCN5C6=CC=CC=C26)CCC15C34 | [#6]12-[#6]-[#6]-[#7]3-[#6]-[#6]-[#6]-[#6]45-[#6]-[#6]-[#7](-[#6]6:[#6]:[#6]:[#6]:[#6]:[#6]:6-1)-[#6]-2(-[#6]-[#6]-4)-[#6]-3-5 | Q131287207 |
| Seco-  schizozygane | CCC1(CCC2)CCC34C1N2CCC3C5=CC=CC=C5N4 | [#6]-[#6]-[#6]12-[#6]-[#6]-[#6]-[#7]3-[#6]-1-[#6]1(-[#6]-[#6]-2)-[#6](-[#6]-[#6]-3)-[#6]2:[#6]:[#6]:[#6]:[#6]:[#6]:2-[#7]-1 | Q131287208 |
| Strictosidine | CCC1C(C(COC1)C)CC2NCCC3=C2NC4=CC=CC=C34 | [#6]-[#6]-[#6]1-[#6](-[#6](-[#6]-[#8]-[#6]-1)-[#6])-[#6]-[#6]1-[#7]-[#6]-[#6]-[#6]2:[#6]-1:[#7H]:[#6]1:[#6]:[#6]:[#6]:[#6]:[#6]:2:1 | Q131287209 |
| Pauridianthinane | C12=CN=CC=C1C(CC2)C3=NC=CC4=C3NC5=C4C=CC=C5 | [#6]12:[#6]:[#7]:[#6]:[#6]:[#6]:1-[#6](-[#6]-[#6]-2)-[#6]1:[#7]:[#6]:[#6]:[#6]2:[#6]:1:[#7H]:[#6]1:[#6]:2:[#6]:[#6]:[#6]:[#6]:1 | Q131287210 |
| Geissolaevane | C12=CC=CC=C1C(C=CN=C3C4OCCC4)=C3N2 | [#6]12:[#6]:[#6]:[#6]:[#6]:[#6]:1:[#6]1:[#6]:[#6]:[#7]:[#6](-[#6]3-[#8]-[#6]-[#6]-[#6]-3):[#6]:1:[#7H]:2 | Q131287212 |
| Strychnidine | CCC1CN2C3CC1C4C(C35CC2)N(CCC4)C6=C5C=CC=C6 | [#6]-[#6]-[#6]1-[#6]-[#7]2-[#6]3-[#6]-[#6]-1-[#6]1-[#6]4-[#6]-3(-[#6]-[#6]-2)-[#6]2:[#6](-[#7]-4-[#6]-[#6]-[#6]-1):[#6]:[#6]:[#6]:[#6]:2 | Q131287213 |
| Uleane | CC1C2CCN(C)C(C2CC)C3=C1NC4=C3C=CC=C4 | [#6]-[#6]1-[#6]2-[#6]-[#6]-[#7](-[#6])-[#6](-[#6]-2-[#6]-[#6])-[#6]2:[#6]-1:[#7H]:[#6]1:[#6]:2:[#6]:[#6]:[#6]:[#6]:1 | Q131287214 |
| Vallesamane | CCC1CN2CC3=C(CC1CC2)NC4=C3C=CC=C4 | [#6]-[#6]-[#6]1-[#6]-[#7]2-[#6]-[#6]3:[#6](-[#6]-[#6]-1-[#6]-[#6]-2):[#7H]:[#6]1:[#6]:3:[#6]:[#6]:[#6]:[#6]:1 | Q131287215 |
| Nor-seco-  vallesamane | CCC1CNCCC1CC2=CC3=CC=CC=C3N2 | [#6]-[#6]-[#6]1-[#6]-[#7]-[#6]-[#6]-[#6]-1-[#6]-[#6]1:[#6]:[#6]2:[#6]:[#6]:[#6]:[#6]:[#6]:2:[#7H]:1 | Q131287217 |
| Vallesiachotamane | CC1C(CC2C3=C(CCN2C1)C(C=CC=C4)=C4N3)C(CC)C | [#6]-[#6]1-[#6](-[#6]-[#6]2-[#6]3:[#6](-[#6]-[#6]-[#7]-2-[#6]-1):[#6]1:[#6]:[#6]:[#6]:[#6]:[#6]:1:[#7H]:3)-[#6](-[#6]-[#6])-[#6] | Q131287218 |
| Vindolinine | C1(NC23CCC4(C5C36CCN5CCC4)C2)=C6C=CC=C1 | [#6]12-[#7]-[#6]34-[#6]-[#6]-[#6]5(-[#6]6-[#6]-3(-[#6]-[#6]-[#7]-6-[#6]-[#6]-[#6]-5)-[#6]:1:[#6]:[#6]:[#6]:[#6]:2)-[#6]-4 | Q131287219 |
| Vobasine | O=C1C2=C(CC3NCCC(C3)C1)C(C=CC=C4)=C4N2 | [#8]=[#6]1-[#6]2:[#6](-[#6]-[#6]3-[#7]-[#6]-[#6]-[#6](-[#6]-3)-[#6]-1):[#6]1:[#6]:[#6]:[#6]:[#6]:[#6]:1:[#7H]:2 | Q131287220 |
| Ervitsinane | O=C1C2=C(C3CC(C1)C(CC)CN3)C4=CC=CC=C4N2 | [#8]=[#6]1-[#6]2:[#6](-[#6]3-[#6]-[#6](-[#6]-1)-[#6](-[#6]-[#6])-[#6]-[#7]-3):[#6]1:[#6]:[#6]:[#6]:[#6]:[#6]:1:[#7H]:2 | Q131287221 |
| Yohimbinoid | C12=C(C=CC=C2)C3=C(C4N(CC3)CC5CCCCC5C4)N1 | [#6]12:[#6](:[#6]:[#6]:[#6]:[#6]:1):[#6]1:[#6](-[#6]3-[#7](-[#6]-[#6]-1)-[#6]-[#6]1-[#6]-[#6]-[#6]-[#6]-[#6]-1-[#6]-3):[#7H]:2 | Q131287222 |
| Criofolinane | C1(NC2=C3CCN(CC4C5CCCC4)C5C2)=C3C=CC=C1 | [#6]12:[#7H]:[#6]3:[#6](-[#6]-[#6]-[#7]4-[#6]-[#6]5-[#6](-[#6]-[#6]-[#6]-[#6]-5)-[#6]-4-[#6]-3):[#6]:1:[#6]:[#6]:[#6]:[#6]:2 | Q131287223 |
| Scholarisiane | C12CNC3CC1CC4(CC2)C3NC5=CC=CC=C45 | [#6]12-[#6]-[#7]-[#6]3-[#6]-[#6]-1-[#6]-[#6]1(-[#6]-[#6]-2)-[#6]-3-[#7]-[#6]2:[#6]:[#6]:[#6]:[#6]:[#6]:2-1 | Q131287224 |
| Goniomitinane | CCC1(CC2)CCCNC1N3C2=CC4=C3C=CC=C4 | [#6]-[#6]-[#6]12-[#6]-[#6]-[#6]3:[#7](-[#6]-1-[#7]-[#6]-[#6]-[#6]-2):[#6]1:[#6](:[#6]:3):[#6]:[#6]:[#6]:[#6]:1 | Q131287225 |
| Spirooxindole-  aspidosperma | CCC1CCCN2CCC3(C(NC4=C3C=CC=C4)=O)C12 | [#6]-[#6]-[#6]1-[#6]-[#6]-[#6]-[#7]2-[#6]-[#6]-[#6]3(-[#6](-[#7]-[#6]4:[#6]-3:[#6]:[#6]:[#6]:[#6]:4)=[#8])-[#6]-1-2 | Q131287226 |
| Seco-  aspidosperma | CCC1(C)CCC2NC3=CC=CC=C3C24C1N(CC4)CC | [#6]-[#6]-[#6]1(-[#6])-[#6]-[#6]-[#6]2-[#7]-[#6]3:[#6]:[#6]:[#6]:[#6]:[#6]:3-[#6]-23-[#6]-1-[#7](-[#6]-[#6]-3)-[#6]-[#6] | Q131287228 |
| Leucolusinane | CCC12CCCN(CCC3C(NC4=CC=CC=C34)=O)C1OCC2 | [#6]-[#6]-[#6]12-[#6]-[#6]-[#6]-[#7](-[#6]-[#6]-[#6]3-[#6](-[#7]-[#6]4:[#6]:[#6]:[#6]:[#6]:[#6]:4-3)=[#8])-[#6]-1-[#8]-[#6]-[#6]-2 | Q131287229 |
| Mersicarpinane | N12CCCC3C1C(C4=CC=CC=C24)NCCC3 | [#7]12-[#6]-[#6]-[#6]-[#6]3-[#6]-1-[#6](-[#6]1:[#6]:[#6]:[#6]:[#6]:[#6]:1-2)-[#7]-[#6]-[#6]-[#6]-3 | Q131287230 |
| Angustinane | CCC1=CN=CC(C2)=C1CC(N2CC3)C4=C3C5=CC=CC=C5N4 | [#6]-[#6]-[#6]1:[#6]:[#7]:[#6]:[#6]2-[#6]-[#7]3-[#6](-[#6]-[#6]:1:2)-[#6]1:[#6](-[#6]-[#6]-3):[#6]2:[#6]:[#6]:[#6]:[#6]:[#6]:2:[#7H]:1 | Q131287232 |
| Fruticosinane | C1(NC23C45C(N(CCC6)CC5CC7C3)C76CC2)=C4C=CC=C1 | [#6]12-[#7]-[#6]34-[#6]5(-[#6]6-[#7]7-[#6]-[#6]-[#6]-[#6]-6(-[#6](-[#6]-[#6]-5-[#6]-7)-[#6]-3)-[#6]-[#6]-4)-[#6]:1:[#6]:[#6]:[#6]:[#6]:2 | Q131287234 |
| Dasyrachinane | C1(NC23C45C(N(CCC6)CC5C(C7)C3)C76CC2)=C4C=CC=C1 | [#6]12-[#7]-[#6]34-[#6]5(-[#6]6-[#7]7-[#6]-[#6]-[#6]-[#6]-6(-[#6]-[#6](-[#6]-5-[#6]-7)-[#6]-3)-[#6]-[#6]-4)-[#6]:1:[#6]:[#6]:[#6]:[#6]:2 | Q131287236 |
| Antirhinane | CCC(C)C1CCN(C2C1)CCC3=C2NC4=C3C=CC=C4 | [#6]-[#6]-[#6](-[#6])-[#6]1-[#6]-[#6]-[#7]2-[#6](-[#6]-1)-[#6]1:[#6](-[#6]-[#6]-2):[#6]2:[#6](:[#7H]:1):[#6]:[#6]:[#6]:[#6]:2 | Q131287237 |
| Sempervirinane | C12=CN3C=CC4=C5C=CC=CC5=NC4=C3C=C1CCCC2 | [#6]12:[#6]:[#7]3:[#6]:[#6]:[#6]4:[#6]5:[#6]:[#6]:[#6]:[#6]:[#6]:5:[#7]:[#6]-4:[#6]:3:[#6]:[#6]:1-[#6]-[#6]-[#6]-[#6]-2 | Q131287238 |
| Vincorinane | CCC1CN2CCC34C5=CC=CC=C5NC23CCC1C4 | [#6]-[#6]-[#6]1-[#6]-[#7]2-[#6]-[#6]-[#6]34-[#6]5:[#6]:[#6]:[#6]:[#6]:[#6]:5-[#7]-[#6]-2-3-[#6]-[#6]-[#6]-1-[#6]-4 | Q131287239 |
| Stemmadeninane | CCC1CN2CCC3=C(NC4=C3C=CC=C4)CC1CC2 | [#6]-[#6]-[#6]1-[#6]-[#7]2-[#6]-[#6]-[#6]3:[#6](:[#7H]:[#6]4:[#6]:3:[#6]:[#6]:[#6]:[#6]:4)-[#6]-[#6]-1-[#6]-[#6]-2 | Q131287240 |
| Seco-strychnidine | CCC1CNCCC23C4C(COCCN4C5=CC=CC=C35)C1CC2 | [#6]-[#6]-[#6]1-[#6]-[#7]-[#6]-[#6]-[#6]23-[#6]4-[#6](-[#6]-[#8]-[#6]-[#6]-[#7]-4-[#6]4:[#6]:[#6]:[#6]:[#6]:[#6]:4-2)-[#6]-1-[#6]-[#6]-3 | Q131287241 |
| Isocorymane | O=C(O1)C2CCC1C34C(C5=CC=CC=C5N4)2CCN3 | [#8]=[#6]1-[#8]-[#6]2-[#6]-[#6]-[#6]-1-[#6]13-[#6]-2(-[#7]-[#6]2:[#6]-1:[#6]:[#6]:[#6]:[#6]:2)-[#7]-[#6]-[#6]-3 | Q131287242 |
| Cymosidane | CCC1COCC2C1CC3NCCC4C5=C(NC234)C=CC=C5 | [#6]-[#6]-[#6]1-[#6]-[#8]-[#6]-[#6]2-[#6]-1-[#6]-[#6]1-[#7]-[#6]-[#6]-[#6]3-[#6]4:[#6](-[#7]-[#6]-2-1-3):[#6]:[#6]:[#6]:[#6]:4 | Q131287243 |
| Arbophyllidinane | CCC1CN2CCC13CC4=C(CC32)C5=CC=CC=C5N4 | [#6]-[#6]-[#6]1-[#6]-[#7]2-[#6]-[#6]-[#6]-13-[#6]-[#6]1:[#6](-[#6]-[#6]-2-3):[#6]2:[#6]:[#6]:[#6]:[#6]:[#6]:2:[#7H]:1 | Q131287244 |
| Arbophyllininane | CCC12CN(C3CC1CCO2)CCC4=C3NC5=CC=CC=C45 | [#6]-[#6]-[#6]12-[#6]-[#7]3-[#6](-[#6]-[#6]-1-[#6]-[#6]-[#8]-2)-[#6]1:[#6](-[#6]-[#6]-3):[#6]2:[#6](:[#7H]:1):[#6]:[#6]:[#6]:[#6]:2 | Q131287245 |
| Arboricinane | CCC1C(CC)CC2N(CCC3=C2NC4=CC=CC=C43)C1 | [#6]-[#6]-[#6]1-[#6](-[#6]-[#6])-[#6]-[#6]2-[#7](-[#6]-[#6]-[#6]3:[#6]-2:[#7H]:[#6]2:[#6]:[#6]:[#6]:[#6]:[#6]:3:2)-[#6]-1 | Q131287246 |
| Arboricininane | CCC1CCN(C2C1CC)CCC3=C2NC4=CC=CC=C43 | [#6]-[#6]-[#6]1-[#6]-[#6]-[#7]2-[#6](-[#6]-1-[#6]-[#6])-[#6]1:[#6](-[#6]-[#6]-2):[#6]2:[#6](:[#7H]:1):[#6]:[#6]:[#6]:[#6]:2 | Q131287247 |
| Apogeissoschizane | CCC1C2CC3C4C(C5=CC=CC=C5N4CC2)CCN3C1 | [#6]-[#6]-[#6]1-[#6]2-[#6]-[#6]3-[#6]4-[#6](-[#6]5:[#6]:[#6]:[#6]:[#6]:[#6]:5-[#7]-4-[#6]-[#6]-2)-[#6]-[#6]-[#7]-3-[#6]-1 | Q131287248 |
| Arboflorinane | O=C1NCC2CCCN3C2C1C4=C(CC3)C5=CC=CC=C5N4 | [#8]=[#6]1-[#7]-[#6]-[#6]2-[#6]-[#6]-[#6]-[#7]3-[#6]-2-[#6]-1-[#6]1:[#6](-[#6]-[#6]-3):[#6]2:[#6]:[#6]:[#6]:[#6]:[#6]:2:[#7H]:1 | Q131287249 |
| Arboduridinane | C12NC3=CC=CC=C3C14CCN5CCC(C5C4)C2 | [#6]12-[#7]-[#6]3:[#6]:[#6]:[#6]:[#6]:[#6]:3-[#6]-13-[#6]-[#6]-[#7]1-[#6]-[#6]-[#6](-[#6]-1-[#6]-3)-[#6]-2 | Q131287251 |
| Alstoscholactane | C12CCNCC1CCC(C3=C(N4)C=CC=C3)=C4C2 | [#6]12-[#6]-[#6]-[#7]-[#6]-[#6]-1-[#6]-[#6]-[#6]1:[#6]3:[#6](:[#7H]:[#6]:1-[#6]-2):[#6]:[#6]:[#6]:[#6]:3 | Q131287252 |
| Mappianinane | C1(NC2=C3CCN(CC4C5CCOC4)C5C2)=C3C=CC=C1 | [#6]12:[#7H]:[#6]3:[#6](-[#6]-[#6]-[#7]4-[#6]-[#6]5-[#6](-[#6]-[#6]-[#8]-[#6]-5)-[#6]-4-[#6]-3):[#6]:1:[#6]:[#6]:[#6]:[#6]:2 | Q131287254 |
| Meloteninane | CC1C2CCC(C34C2N(CCC1)CC4)NC5=C3C=CC=C5 | [#6]-[#6]1-[#6]2-[#6]-[#6]-[#6]3-[#6]4(-[#6]-2-[#7](-[#6]-[#6]-[#6]-1)-[#6]-[#6]-4)-[#6]1:[#6](-[#7]-3):[#6]:[#6]:[#6]:[#6]:1 | Q131287256 |
| Iboluteninane | CCC1CC2CC3C1N(CCC34NC5=C(C=CC=C5)C4=O)C2 | [#6]-[#6]-[#6]1-[#6]-[#6]2-[#6]-[#6]3-[#6]-1-[#7](-[#6]-[#6]-[#6]-31-[#7]-[#6]3:[#6](:[#6]:[#6]:[#6]:[#6]:3)-[#6]-1=[#8])-[#6]-2 | Q131287258 |
| Ervaoffinane | O=C1C2N(CCN3C4C2CC(CC4)C3)C5=C1C=CC=C5 | [#8]=[#6]1-[#6]2-[#7](-[#6]-[#6]-[#7]3-[#6]4-[#6]-2-[#6]-[#6](-[#6]-[#6]-4)-[#6]-3)-[#6]2:[#6]-1:[#6]:[#6]:[#6]:[#6]:2 | Q131287259 |
| Nor-iboga | CCC1C2N3CC(C1)CC2C4=C(C5=C(C=CC=C5)N4)C3 | [#6]-[#6]-[#6]1-[#6]2-[#7]3-[#6]-[#6](-[#6]-1)-[#6]-[#6]-2-[#6]1:[#6](:[#6]2:[#6](:[#6]:[#6]:[#6]:[#6]:2):[#7H]:1)-[#6]-3 | Q131287260 |
| Nor-seco-iboga | CCC1C2NCC(C1)CC2C3=CC4=C(C=CC=C4)N3 | [#6]-[#6]-[#6]1-[#6]2-[#7]-[#6]-[#6](-[#6]-1)-[#6]-[#6]-2-[#6]1:[#6]:[#6]2:[#6](:[#6]:[#6]:[#6]:[#6]:2):[#7H]:1 | Q131287261 |
| Voatingginane | C1(NC23C4CCN2CC5CCCC3C5)=C4C=CC=C1 | [#6]12-[#7]-[#6]34-[#6](-[#6]-[#6]-[#7]-3-[#6]-[#6]3-[#6]-[#6]-[#6]-[#6]-4-[#6]-3)-[#6]:1:[#6]:[#6]:[#6]:[#6]:2 | Q131287263 |
| Isoconolutinane | CCC12CCC34N(C5=CC=CC=C5C3CCN4CCC2)C1 | [#6]-[#6]-[#6]12-[#6]-[#6]-[#6]34-[#7](-[#6]5:[#6]:[#6]:[#6]:[#6]:[#6]:5-[#6]-3-[#6]-[#6]-[#7]-4-[#6]-[#6]-[#6]-1)-[#6]-2 | Q131287264 |
| Hunteracinane | CCC1C[N+]23CCC4C5=C(C=CC=C5)NC24CC1CC3 | [#6]-[#6]-[#6]1-[#6]-[#7+]23-[#6]-[#6]-[#6]4-[#6]5:[#6](:[#6]:[#6]:[#6]:[#6]:5)-[#7]-[#6]-2-4-[#6]-[#6]-1-[#6]-[#6]-3 | Q131287265 |
| Koumine | CCC12CNC3CC1CCC(NC4=C5C=CC=C4)C25C3 | [#6]-[#6]-[#6]12-[#6]-[#7]-[#6]3-[#6]-[#6]-1-[#6]-[#6]-[#6]1-[#7]-[#6]4:[#6](:[#6]:[#6]:[#6]:[#6]:4)-[#6]-2-1-[#6]-3 | Q131287266 |
| Tacamane | CCC1CC2CCN3C4=C(CCN(C42)C1)C5=C3C=CC=C5 | [#6]-[#6]-[#6]1-[#6]-[#6]2-[#6]-[#6]-[#7]3:[#6]4:[#6](-[#6]-[#6]-[#7](-[#6]-2-4)-[#6]-1):[#6]1:[#6]:3:[#6]:[#6]:[#6]:[#6]:1 | Q131287267 |
| Tronocarpinane | C1(CCNCC2(CCC3)CC3C4)=C2N4C5=C1C=CC=C5 | [#6]12-[#6]-[#6]-[#7]-[#6]-[#6]34-[#6]-[#6]-[#6]-[#6](-[#6]-3)-[#6]-[#7](:[#6]:1-4):[#6]1:[#6]:2:[#6]:[#6]:[#6]:[#6]:1 | Q131287268 |
| Secodine | CCC1CCCN(CCC2=C(NC3=C2C=CC=C3)CC)C1 | [#6]-[#6]-[#6]1-[#6]-[#6]-[#6]-[#7](-[#6]-[#6]-[#6]2:[#6](:[#7H]:[#6]3:[#6]:2:[#6]:[#6]:[#6]:[#6]:3)-[#6]-[#6])-[#6]-1 | Q131287269 |
| Meloscandoninane | CC1CC2(C34C5C(CCCN5CC4)1C2)CNC6=C3C=CC=C6 | [#6]-[#6]1-[#6]-[#6]23-[#6]4(-[#6]5-[#6]-1(-[#6]-[#6]-[#6]-[#7]-5-[#6]-[#6]-4)-[#6]-2)-[#6]1:[#6](-[#7]-[#6]-3):[#6]:[#6]:[#6]:[#6]:1 | Q131287270 |
| Spirooxindole-  eburna | CCC12CCCN3CCC4(N(C5=C(C4=O)C=CC=C5)CC2)C13 | [#6]-[#6]-[#6]12-[#6]-[#6]-[#6]-[#7]3-[#6]-[#6]-[#6]4(-[#7](-[#6]5:[#6](-[#6]-4=[#8]):[#6]:[#6]:[#6]:[#6]:5)-[#6]-[#6]-1)-[#6]-2-3 | Q131287271 |
| Meloyuninane | CCC12CCN3C4=C(C(C5=CC=CC=C35)=O)CN(CCC2)C41 | [#6]-[#6]-[#6]12-[#6]-[#6]-[#7]3:[#6]4:[#6](:[#6](:[#6]5:[#6]:[#6]:[#6]:[#6]:[#6]:3:5)=[#8])-[#6]-[#7](-[#6]-[#6]-[#6]-1)-[#6]-2-4 | Q131287272 |
| Gelsemamidane | NC1=C(C23CC4C5COC2CC5C(CC)CN4C3=O)C=CC=C1 | [#7]-[#6]1:[#6](-[#6]23-[#6]-[#6]4-[#6]5-[#6]-[#8]-[#6]-2-[#6]-[#6]-5-[#6](-[#6]-[#6])-[#6]-[#7]-4-[#6]-3=[#8]):[#6]:[#6]:[#6]:[#6]:1 | Q131287273 |
| Humanteninane | O=C1NC2=C(C=CC=C2)C13CC4CC(C(CC)CN4)CC3 | [#8]=[#6]1-[#7]-[#6]2:[#6](:[#6]:[#6]:[#6]:[#6]:2)-[#6]-12-[#6]-[#6]1-[#6]-[#6](-[#6](-[#6]-[#6])-[#6]-[#7]-1)-[#6]-[#6]-2 | Q131287274 |
| Seco-koumine | CNC1CC(CCC2NC3=C4C=CC=C3)C(CC)C24C1 | [#6]-[#7]-[#6]1-[#6]-[#6]2-[#6]-[#6]-[#6]3-[#7]-[#6]4:[#6](:[#6]:[#6]:[#6]:[#6]:4)-[#6]-3(-[#6]-2-[#6]-[#6])-[#6]-1 | Q131287275 |
| Flabelliformidane | CCC1CNCC2C1CCC3=C(C4=C(N3)C=CC=C4)CC2 | [#6]-[#6]-[#6]1-[#6]-[#7]-[#6]-[#6]2-[#6]-1-[#6]-[#6]-[#6]1:[#6](:[#6]3:[#6](:[#7H]:1):[#6]:[#6]:[#6]:[#6]:3)-[#6]-[#6]-2 | Q131287276 |
| Pandinane | C1(NC2C34CCN(C5CC6)C4C6C5C2)=C3C=CC=C1 | [#6]12-[#7]-[#6]3-[#6]4(-[#6]-[#6]-[#7]5-[#6]6-[#6]-[#6]-[#6](-[#6]-4-5)-[#6]-6-[#6]-3)-[#6]:1:[#6]:[#6]:[#6]:[#6]:2 | Q131287277 |
| Cleavincathicane | CC1C2=NC3=C(C24C5C(C1)CN(CC5CC)CC4)C=CC=C3 | [#6]-[#6]1-[#6]2=[#7]-[#6]3:[#6](-[#6]-24-[#6]2-[#6](-[#6]-1)-[#6]-[#7](-[#6]-[#6]-2-[#6]-[#6])-[#6]-[#6]-4):[#6]:[#6]:[#6]:[#6]:3 | Q131287278 |
| Cleavincaroseane | CCC(C1)C2CCN1CCC3C4=C(N=C32)C=CC=C4 | [#6]-[#6]-[#6]1-[#6]-[#7]2-[#6]-[#6]-[#6]-1-[#6]1-[#6](-[#6]-[#6]-2)-[#6]2:[#6](-[#7]=1):[#6]:[#6]:[#6]:[#6]:2 | Q131287279 |
| Kopsifolane | C1(NC2C3CC4(C5C26CCN5CCC4)CC3)=C6C=CC=C1 | [#6]12-[#7]-[#6]3-[#6]4-[#6]-[#6]5(-[#6]6-[#6]-3(-[#6]-[#6]-[#7]-6-[#6]-[#6]-[#6]-5)-[#6]:1:[#6]:[#6]:[#6]:[#6]:2)-[#6]-[#6]-4 | Q131287280 |
| Spirooxindole sarpagine | CCC(CN12)C3CC2C4(C(NC5=CC=CC=C54)=O)CC1C3 | [#6]-[#6]-[#6]1-[#6]-[#7]2-[#6]3-[#6]-[#6]-1-[#6]-[#6]-2-[#6]-[#6]-31-[#6](-[#7]-[#6]2:[#6]:[#6]:[#6]:[#6]:[#6]:2-1)=[#8] | Q131287281 |
| Meloyinane | CC12NC3=C(C=CC=C3)C1(CCN4CCC5)C4C65C(C)C2C6 | [#6]-[#6]12-[#7]-[#6]3:[#6](:[#6]:[#6]:[#6]:[#6]:3)-[#6]-13-[#6]-[#6]-[#7]1-[#6]-[#6]-[#6]-[#6]4(-[#6]-3-1)-[#6](-[#6])-[#6]-2-[#6]-4 | Q131287283 |
| Cleavamine | CCC1CC2CCC3=C(CCN(C2)C1)C4=CC=CC=C4N3 | [#6]-[#6]-[#6]1-[#6]-[#6]2-[#6]-[#6]-[#6]3:[#6](-[#6]-[#6]-[#7](-[#6]-2)-[#6]-1):[#6]1:[#6]:[#6]:[#6]:[#6]:[#6]:1:[#7H]:3 | Q131287284 |
| Cleaisovincathicane | CCC12CC3CCC4=NC5=CC=CC=C5C14CCN(C2)C3 | [#6]-[#6]-[#6]12-[#6]-[#6]3-[#6]-[#6]-[#6]4=[#7]-[#6]5:[#6]:[#6]:[#6]:[#6]:[#6]:5-[#6]-1-4-[#6]-[#6]-[#7](-[#6]-2)-[#6]-3 | Q131287285 |
| Cleavincazalidane | CCC1(C2)C[N+]34CC2CC(C4(OC)O1)C([N]C5=C6C=CC=C5)=C6CC3 | [#6]-[#6]-[#6]12-[#6]-[#6]3-[#6]-[#7+]4(-[#6]-1)-[#6](-[#6](-[#6]-3)-[#6]1-[#7]-[#6]3:[#6](:[#6]:[#6]:[#6]:[#6]:3)-[#6]=1-[#6]-[#6]-4)(-[#8]-[#6])-[#8]-2 | Q131287286 |
| Ellipticinane | CC1=C2C=CN=CC2=C(C)C3=C1NC4=CC=CC=C43 | [#6]-[#6]1:[#6]2:[#6]:[#6]:[#7]:[#6]:[#6]:2:[#6](-[#6]):[#6]2:[#6]:1:[#7H]:[#6]1:[#6]:[#6]:[#6]:[#6]:[#6]:2:1 | Q131287287 |
| Isopandolinane | CCC1(O)CN2CCC34C5=C(NC3=C(C)CC2(C4)C1)C=CC=C5 | [#6]-[#6]-[#6]1(-[#8])-[#6]-[#7]2-[#6]-[#6]-[#6]34-[#6]5:[#6](-[#7]-[#6]-3=[#6](-[#6])-[#6]-[#6]-2(-[#6]-4)-[#6]-1):[#6]:[#6]:[#6]:[#6]:5 | Q131287288 |
| Seco-  stemmadeninane | CC(C1=CC=CC=C1N2)=C2[C@@H](C)[C@@H](CCN(C)C/3)C3=C/C | [#6]-[#6]1:[#6]2:[#6]:[#6]:[#6]:[#6]:[#6]:2:[#7H]:[#6]:1-[#6@@H](-[#6])-[#6@H]1-[#6]-[#6]-[#7](-[#6])-[#6]/[#6]-1=[#6]/[#6] | Q131287289 |
| Nor-seco-  stemmadeninane | CC(C1=CC=CC=C1N2)=C2[C@@H](C)[C@@H](CCNC/3)C3=C/C | [#6]-[#6]1:[#6]2:[#6]:[#6]:[#6]:[#6]:[#6]:2:[#7H]:[#6]:1-[#6@@H](-[#6])-[#6@H]1-[#6]-[#6]-[#7]-[#6]/[#6]-1=[#6]/[#6] | Q131287290 |
| Dinor-seco-  stemmadeninane | C/C=C1[C@@H]([C@H](C)C2=CC3=CC=CC=C3N2)CC[N]C\1 | [#6]/[#6]=[#6]1\[#6@@H](-[#6@H](-[#6])-[#6]2:[#6]:[#6]3:[#6]:[#6]:[#6]:[#6]:[#6]:3:[#7H]:2)-[#6]-[#6]-[#7]-[#6]-1 | Q131287292 |
| Mersidasinane | C12CCC(CCCN3CC4)C3C14C(C=CC=C5)=C5NC2 | [#6]12-[#6]-[#6]-[#6]3-[#6]-[#6]-[#6]-[#7]4-[#6]-[#6]-[#6]-1(-[#6]-3-4)-[#6]1:[#6]:[#6]:[#6]:[#6]:[#6]:1-[#7]-[#6]-2 | Q131287293 |
| Naulafinane | O=C1C2CNCC3CCC(C4N1CCC5=C4NC6=C5C=CC=C6)C32 | [#8]=[#6]1-[#6]2-[#6]-[#7]-[#6]-[#6]3-[#6]-[#6]-[#6](-[#6]4-[#7]-1-[#6]-[#6]-[#6]1:[#6]-4:[#7H]:[#6]4:[#6]:1:[#6]:[#6]:[#6]:[#6]:4)-[#6]-2-3 | Q131287294 |
| Lundurane | C12=C(C34CCN5CCCC56CCC3(N2)C4C6)C=CC=C1 | [#6]12:[#6](-[#6]34-[#6]-[#6]-[#7]5-[#6]-[#6]-[#6]-[#6]-56-[#6]-[#6]-[#6]-3(-[#7]-1)-[#6]-4-[#6]-6):[#6]:[#6]:[#6]:[#6]:2 | Q131287296 |
| Angustinane | CCC1=CN=CC2=C1CC3N(CCC4=C3NC5=CC=CC=C45)C2 | [#6]-[#6]-[#6]1:[#6]:[#7]:[#6]:[#6]2:[#6]:1-[#6]-[#6]1-[#7](-[#6]-[#6]-[#6]3:[#6]-1:[#7H]:[#6]1:[#6]:[#6]:[#6]:[#6]:[#6]:3:1)-[#6]-2 | Q131287298 |


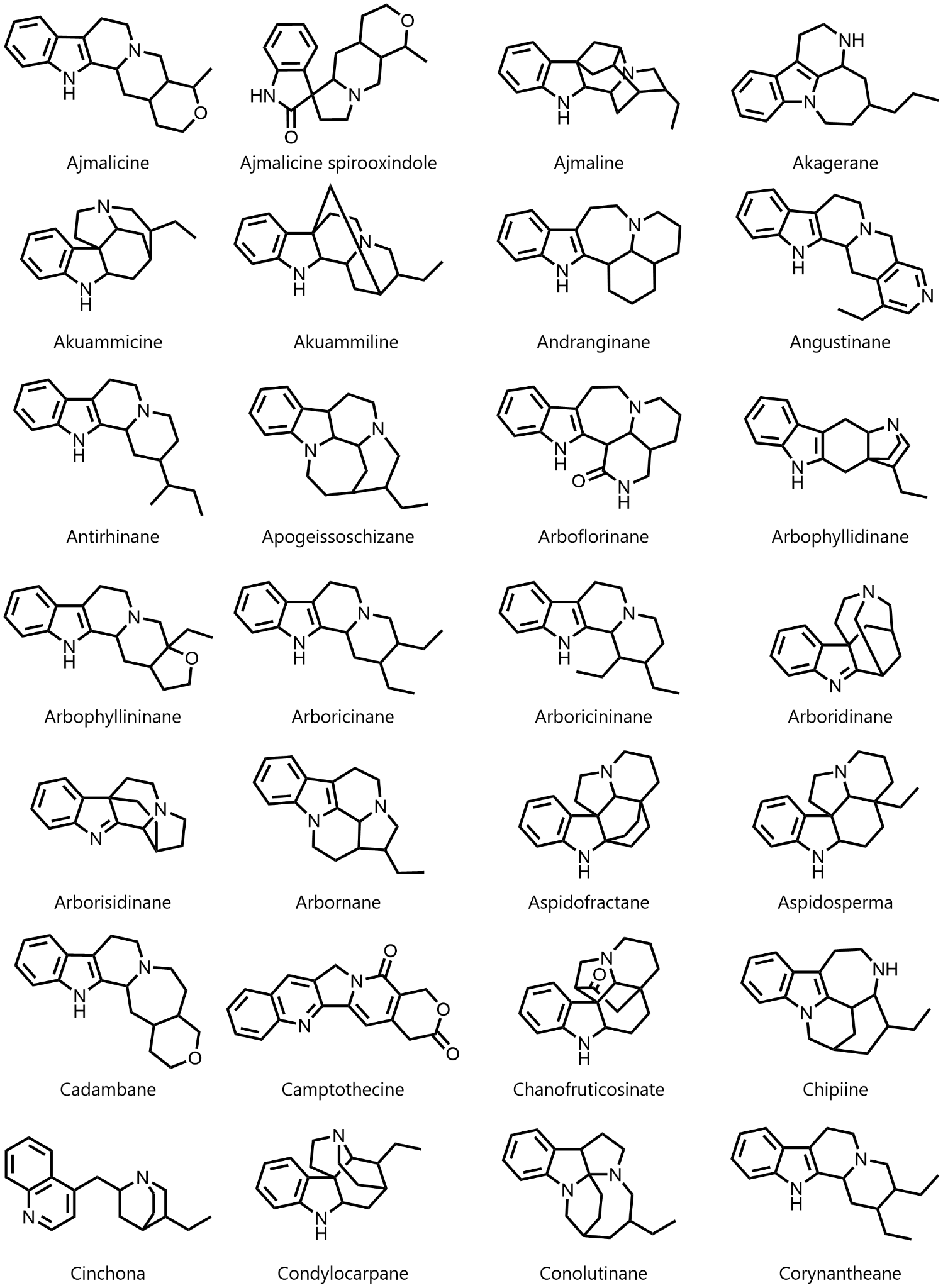


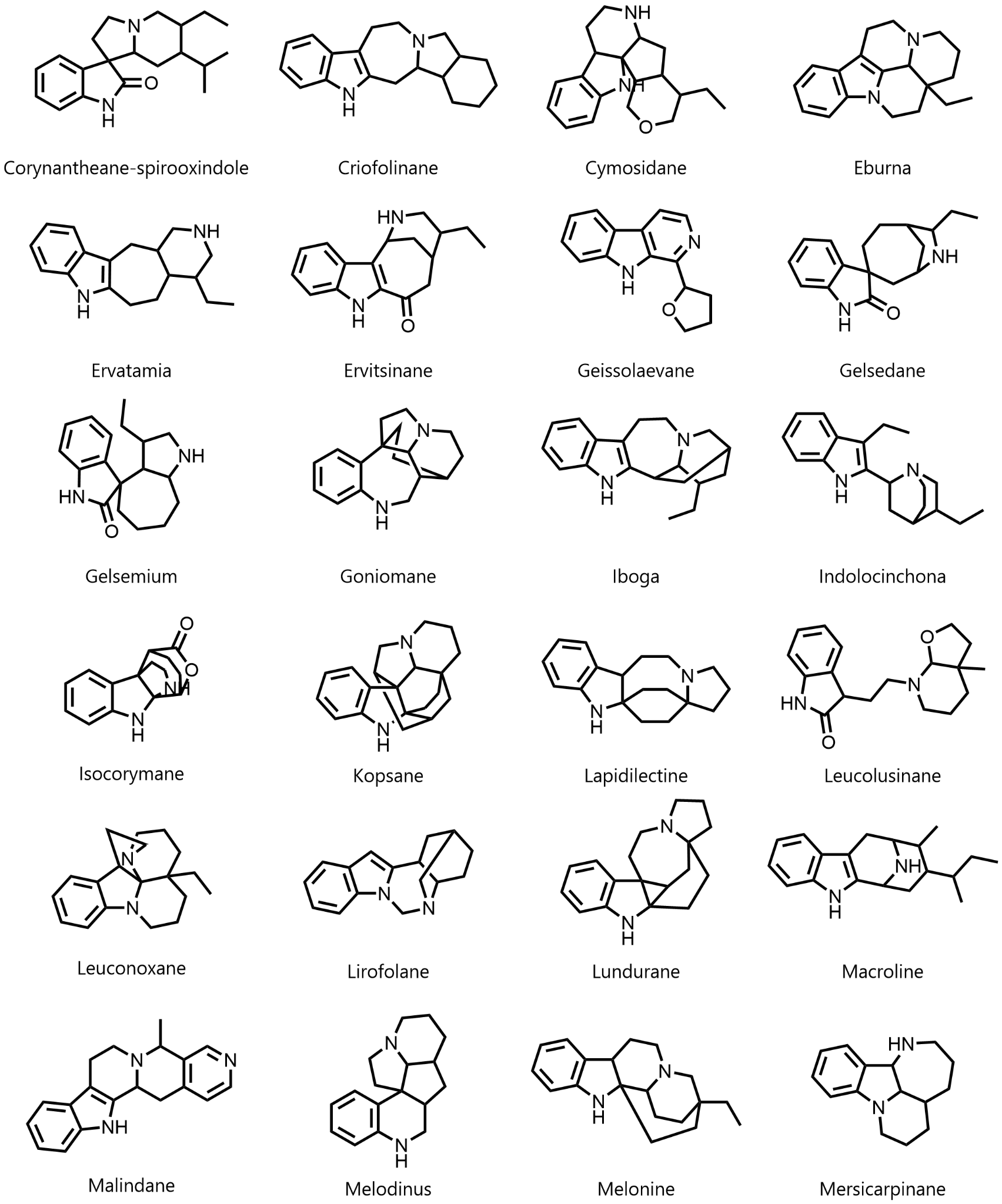

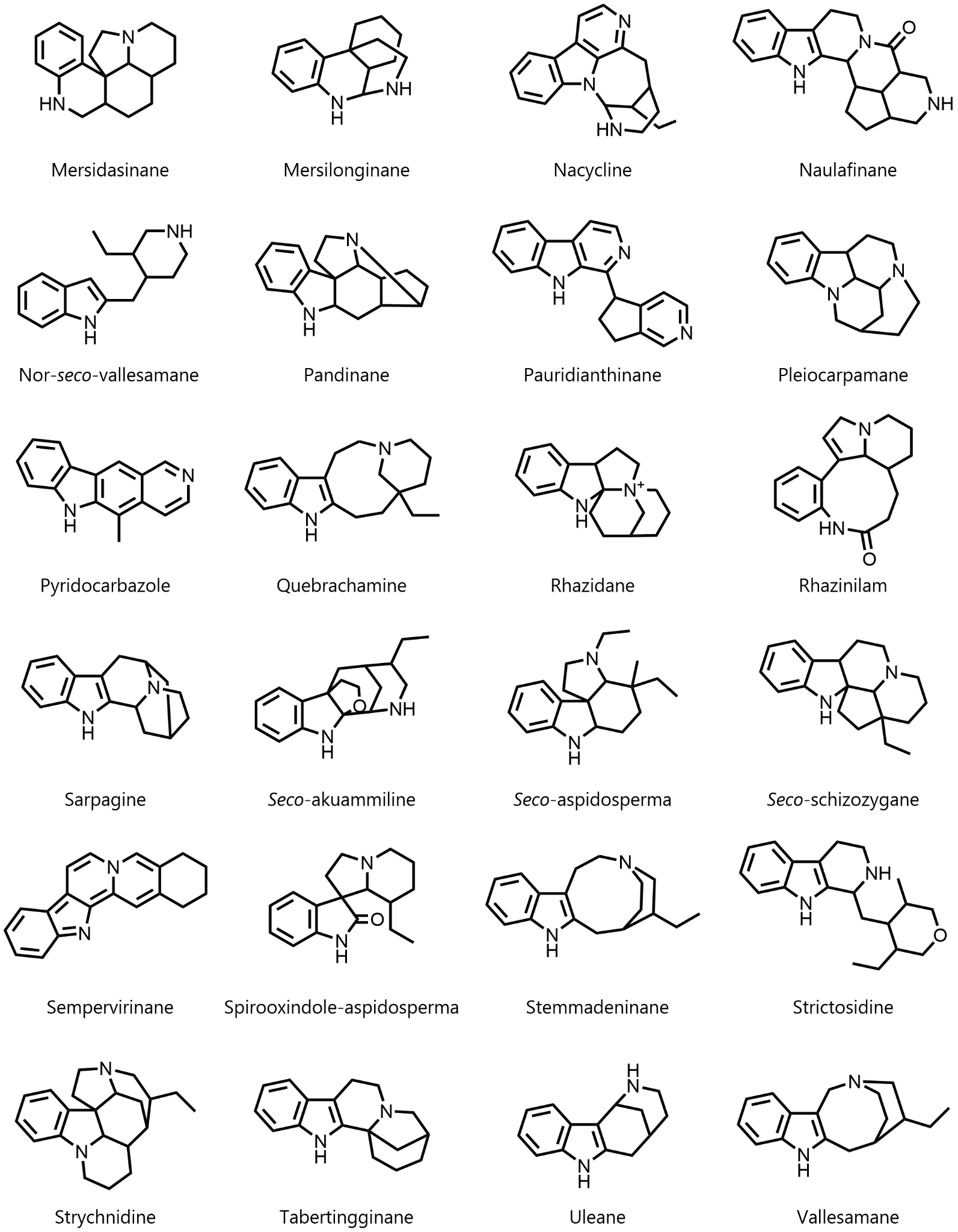

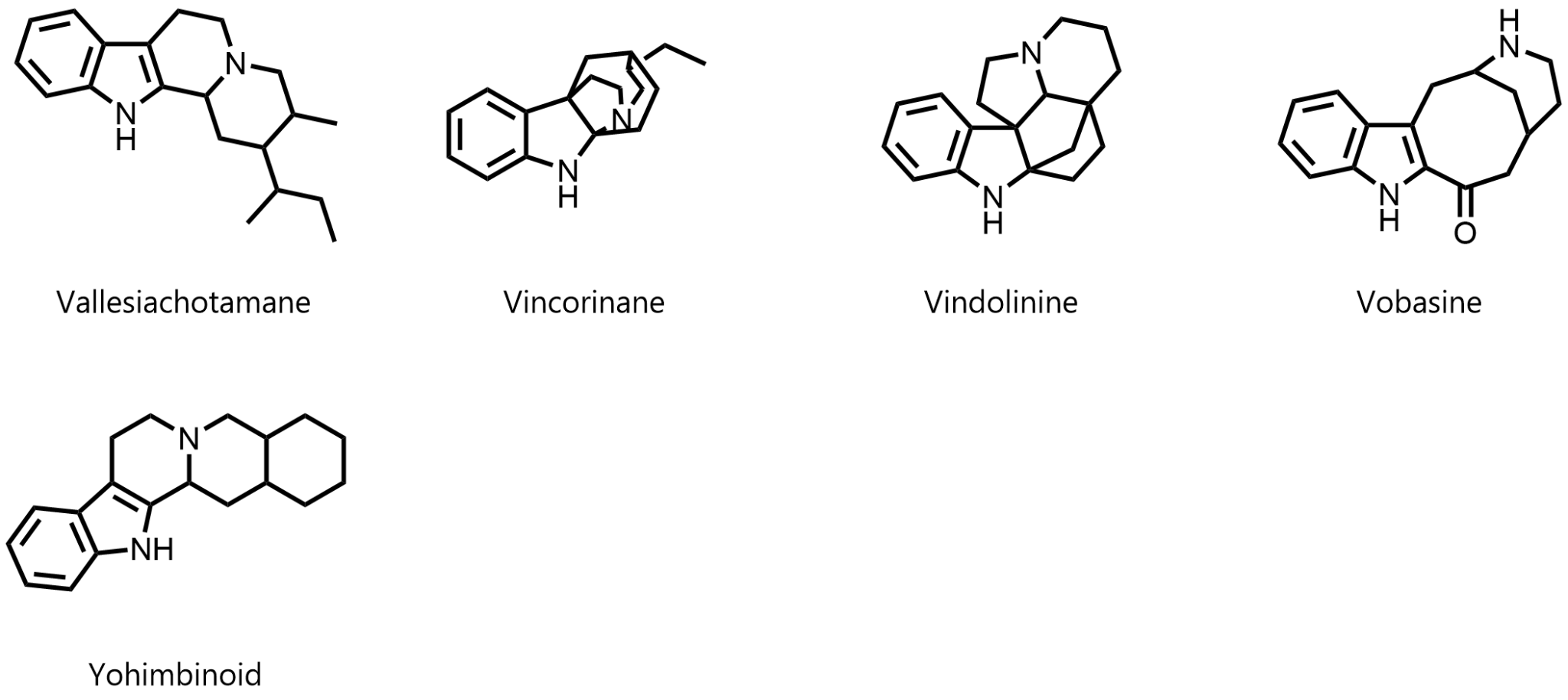


Fig. S12 Monoterpene Indole Alkaloids skeletons included in the MIADB


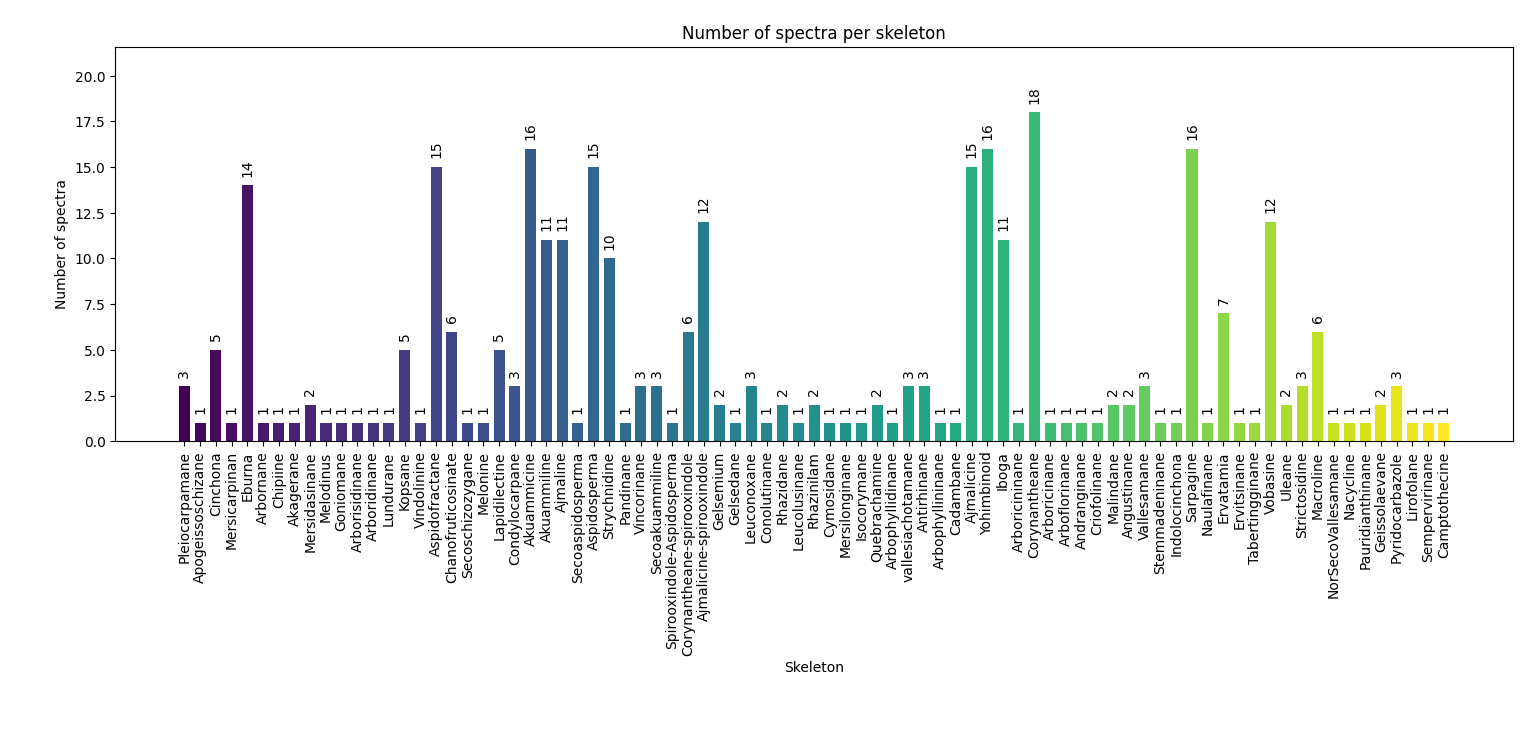


Fig. S13 Number of spectra per skeleton included in the MIADB


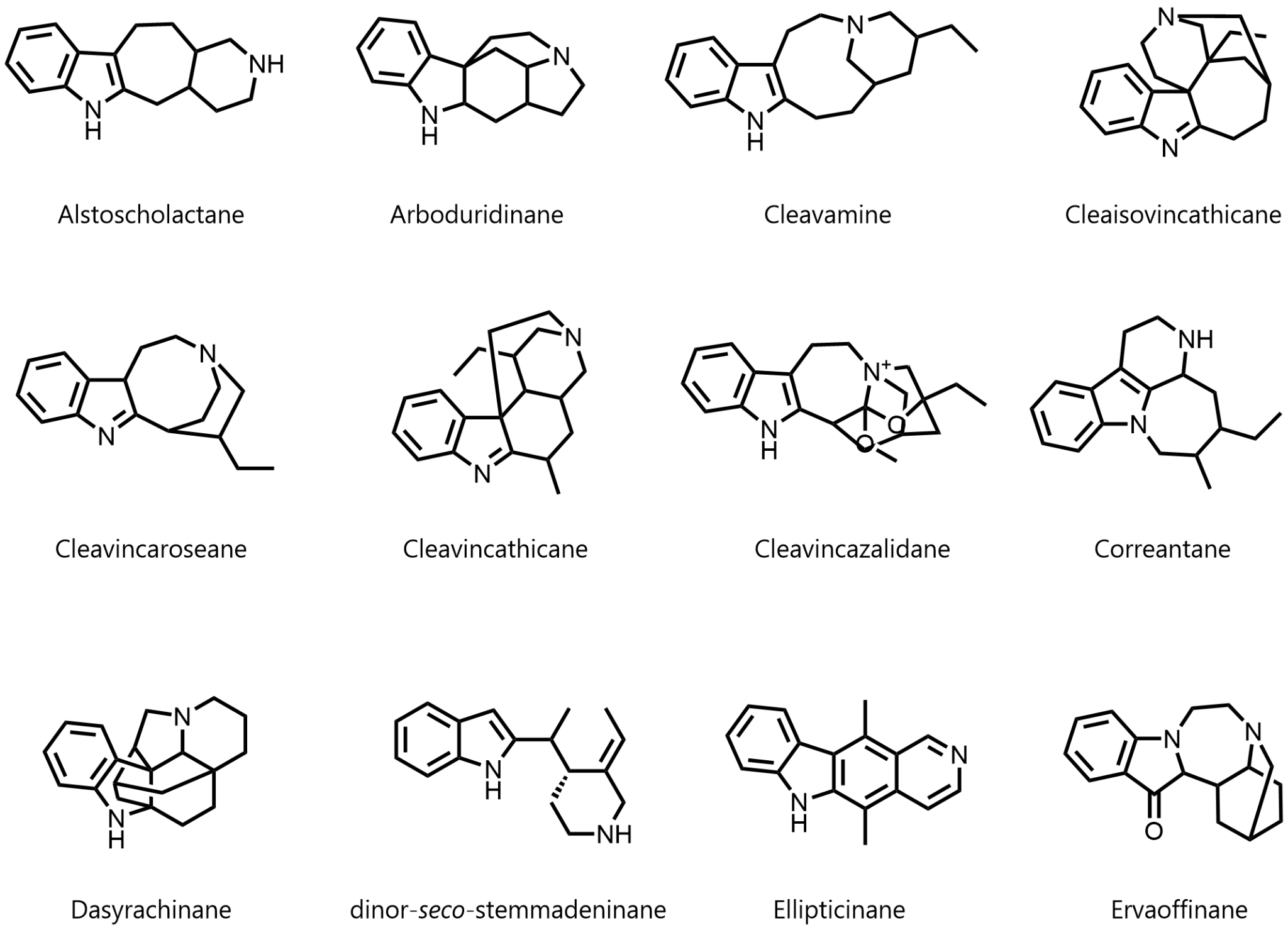

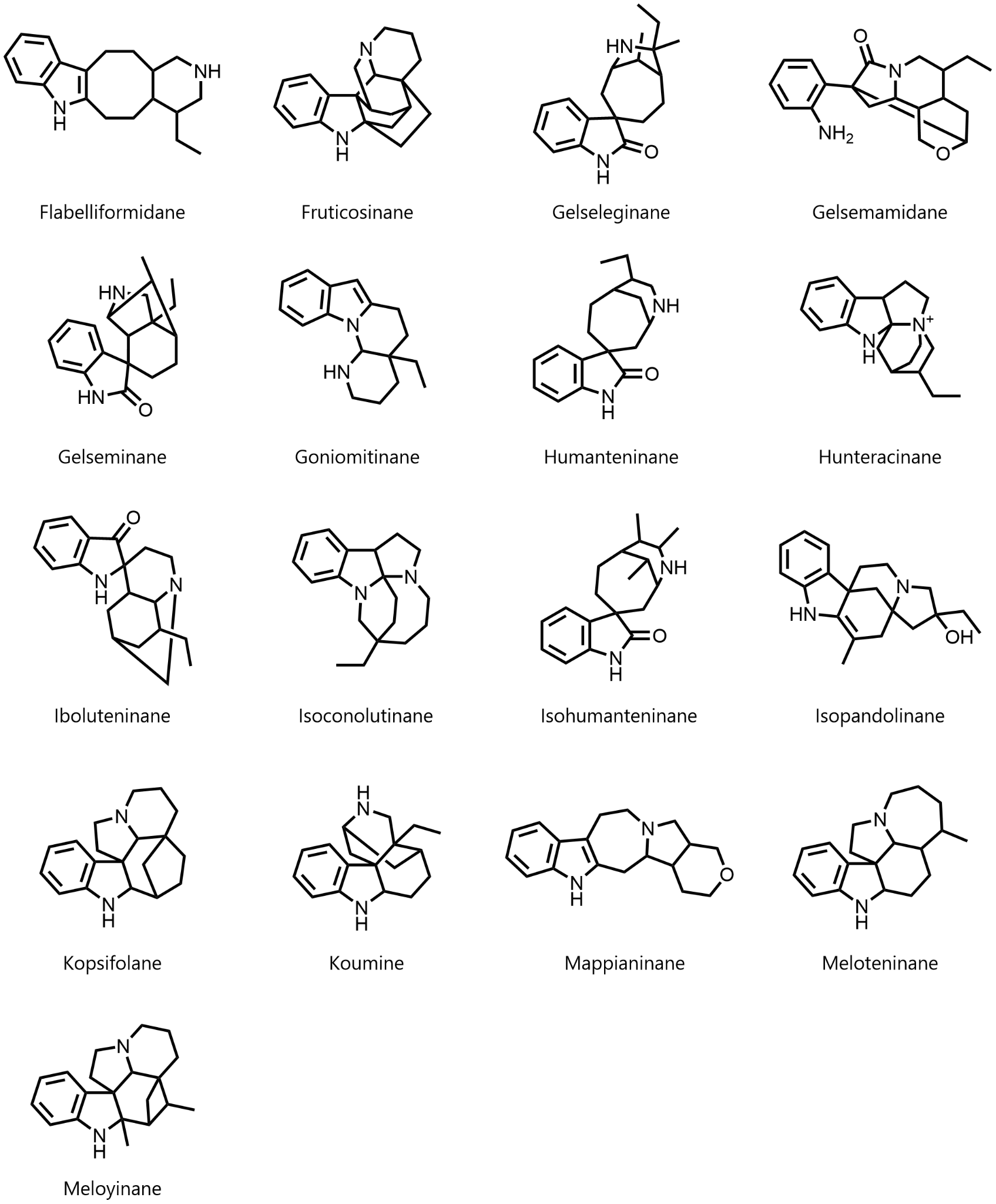


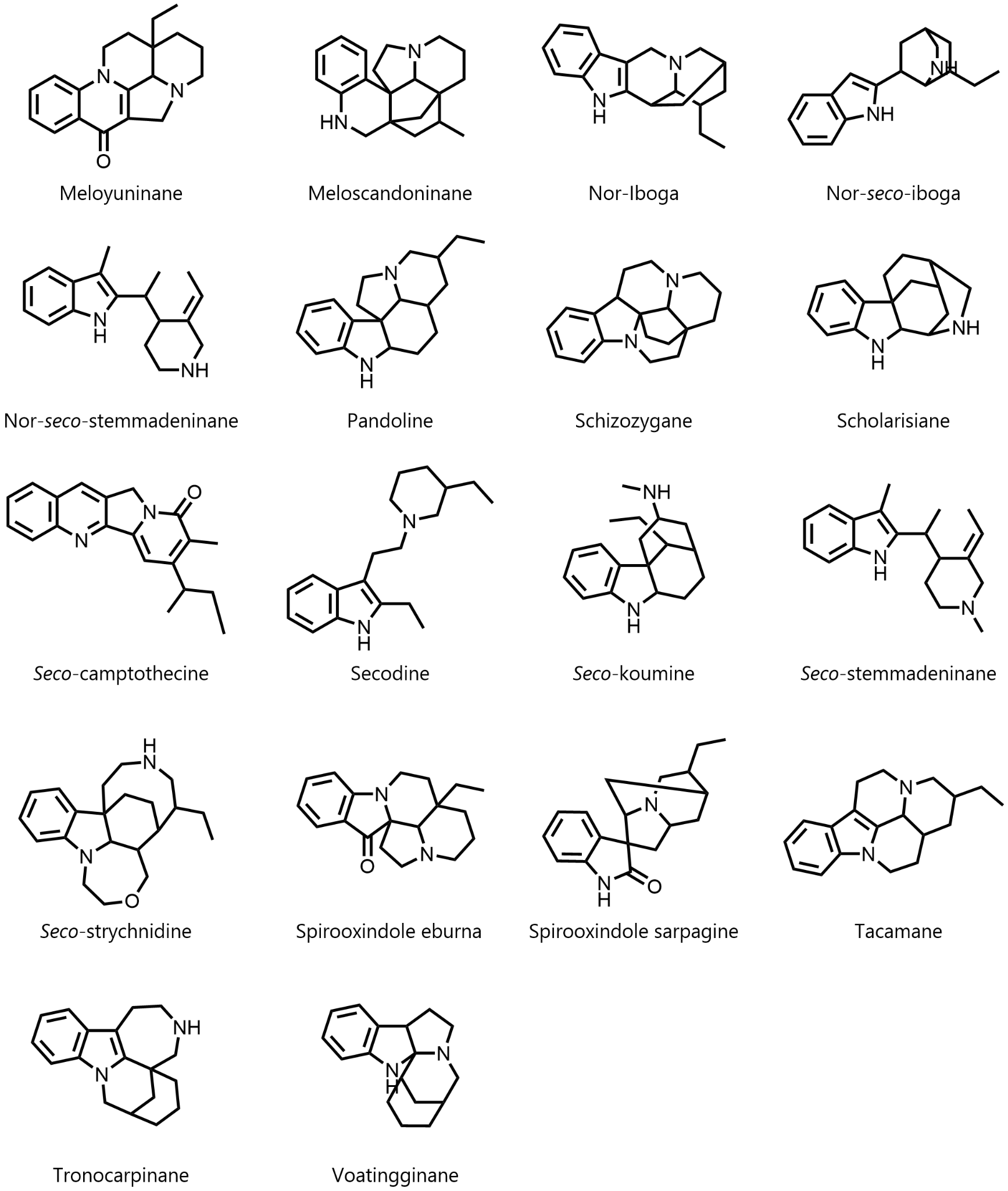


Fig. S14 Additional Monoterpene Indole Alkaloids skeletons

Table S2 Plant extracts selected for analysis

| sample_id | biosource | species_qid | source_botanical_family | family_qid |
| --- | --- | --- | --- | --- |
| AP_018-023 | *Catharanthus roseus* | Q161093 | Apocynaceae | Q173756 |
| AP_075-051 | *Melodinus cochinchinensis* | Q15353708 | Apocynaceae | Q173756 |
| AP_077-022 | *Melodinus honbaensis* | Q15353681 | Apocynaceae | Q173756 |
| AP_077-082 | *Tabernaemontana bovina* | Q15377309 | Apocynaceae | Q173756 |
| AP_086-026 | *Hunteria zeylanica* | Q10884098 | Apocynaceae | Q173756 |
| AP_098-065 | *Spirolobium cambodianum* | Q15613616 | Apocynaceae | Q173756 |
| AP_101-072 | *Alstonia macrophylla* | Q1944104 | Apocynaceae | Q173756 |
| AP_106-088 | *Alstonia mairei* | Q15225128 | Apocynaceae | Q173756 |
| AP_114-094 | *Melodinus fusiformis* | Q15225115 | Apocynaceae | Q173756 |
| AP_116-012 | *Tabernaemontana granatum** |  | Apocynaceae | Q173756 |
| AP_122-024 | *Melodinus cochinchinensis* | Q15353708 | Apocynaceae | Q173756 |
| AP_122-057 | *Tabernaemontana pauciflora* | Q14862282 | Apocynaceae | Q173756 |
| AP_126-012 | *Melodinus cochinchinensis* | Q15353708 | Apocynaceae | Q173756 |
| AP_126-014 | *Melodinus cochinchinensis* | Q15353708 | Apocynaceae | Q173756 |
| AP_130-028 | *Melodinus cochinchinensis* | Q15353708 | Apocynaceae | Q173756 |
| AP_134-057 | *Rauvolfia micrantha* | Q15376032 | Apocynaceae | Q173756 |
| AP_152-029 | *Melodinus cochinchinensis* | Q15353708 | Apocynaceae | Q173756 |
| AP_203-046 | *Rauvolfia littoralis* | Q15374366 | Apocynaceae | Q173756 |
| AP_210-014 | *Stemmadenia pubescens* | Q15376776 | Apocynaceae | Q173756 |
| AP_210-087 | *Trachelospermum asiaticum* | Q11238988 | Apocynaceae | Q173756 |
| AP_230-012 | *Plumeria obtusa* | Q956237 | Apocynaceae | Q173756 |
| AP_266-039 | *Melodinus orientalis* | Q15367892 | Apocynaceae | Q173756 |
| AP_267-048 | *Tabernaemontana bovina* | Q15377309 | Apocynaceae | Q173756 |
| AP_268-056 | *Vincetoxicum flexuosum* | Q65951333 | Apocynaceae | Q173756 |
| AP_287-008 | *Dyera costulata* | Q2714937 | Apocynaceae | Q173756 |
| AP_294-055 | *Cynanchum pulchellum* | Q42684136 | Apocynaceae | Q173756 |
| AP_296-068 | *Rauvolfia verticillata* | Q14861850 | Apocynaceae | Q173756 |
| AP_309-054 | *Pagiantha curvisepala** |  | Apocynaceae | Q173756 |
| AP_316-020 | *Rauvolfia tetraphylla* | Q3595081 | Apocynaceae | Q173756 |
| AP_325-088 | *Melodinus fusiformis* | Q15225115 | Apocynaceae | Q173756 |
| AP_336-068 | *Melodinus cochinchinensis* | Q15353708 | Apocynaceae | Q173756 |
| AP_338-028 | *Leuconotis eugeniifolia* | Q15359729 | Apocynaceae | Q173756 |
| AP_350-045 | *Kopsia singapurensis* | Q5457406 | Apocynaceae | Q173756 |
| LO_074-096 | *Strychnos ovata* | Q15235649 | Loganiaceae | Q500842 |
| LO_152-095 | *Strychnos axillaris* | Q15248035 | Loganiaceae | Q500842 |
| LO_175-003 | *Strychnos minor* | Q18076290 | Loganiaceae | Q500842 |
| LO_175-017 | *Strychnos sonlaensis* | Q18075480 | Loganiaceae | Q500842 |
| LO_175-084 | *Strychnos thorelii* | Q18074753 | Loganiaceae | Q500842 |
| LO_214-072 | *Strychnos chlorantha* | Q15347551 | Loganiaceae | Q500842 |
| LO_277-069 | *Cynanchum purpureum* | Q15225375 | Loganiaceae | Q500842 |
| LO_284-059 | *Strychnos angustiflora* | Q15224463 | Loganiaceae | Q500842 |
| LO_348-033 | *Strychnos cathayensis* | Q10906001 | Loganiaceae | Q500842 |
| LO_351-003 | *Gardenia linifolia** |  | Loganiaceae | Q500842 |
| RU_032-005 | *Mitragyna rotundifolia* | Q11060537 | Rubiaceae | Q156569 |
| RU_051-026 | *Neolamarckia cadamba* | Q573791 | Rubiaceae | Q156569 |
| RU_086-061 | *Adina pilulifera* | Q11129706 | Rubiaceae | Q156569 |
| RU_101-090 | *Neonauclea purpurea* | Q15476837 | Rubiaceae | Q156569 |
| RU_106-016 | *Mitragyna hirsuta* | Q15472603 | Rubiaceae | Q156569 |
| RU_114-006 | *Cinchona officinalis* | Q3091779 | Rubiaceae | Q156569 |
| RU_122-055 | *Timonius flavescens* | Q15460460 | Rubiaceae | Q156569 |
| RU_126-003 | *Uncaria scandens* | Q11078794 | Rubiaceae | Q156569 |
| RU_126-053 | *Nauclea orientalis* | Q1894456 | Rubiaceae | Q156569 |
| RU_139-047 | *Neonauclea sessilifolia* | Q11085620 | Rubiaceae | Q156569 |
| RU_142-063 | *Psychotria peduncularis* var. *peduncularis* | Q104119589 | Rubiaceae | Q156569 |
| RU_152-009 | *Timonius arboreus* | Q15245847 | Rubiaceae | Q156569 |
| RU_152-100 | *Mitragyna hirsuta* | Q15472603 | Rubiaceae | Q156569 |
| RU_231-090 | *Morinda angustifolia* | Q11177601 | Rubiaceae | Q156569 |
| RU_246-060 | *Palicourea azurea* | Q1330547 | Rubiaceae | Q156569 |
| RU_255-018 | *Cephalanthus occidentalis* | Q4992358 | Rubiaceae | Q156569 |
| RU_276-044 | *Ophiorrhiza baviensis* | Q15463501 | Rubiaceae | Q156569 |
| RU_281-013 | *Guettarda hirsuta* | Q15458480 | Rubiaceae | Q156569 |
| RU_282-013 | *Palicourea amethystina* | Q15482482 | Rubiaceae | Q156569 |
| RU_305-020 | *Isertia laevis* | Q15464388 | Rubiaceae | Q156569 |
| RU_308-020 | *Chimarrhis parviflora* | Q15399935 | Rubiaceae | Q156569 |
| RU_316-015 | *Spermacoce ovalifolia* | Q15451632 | Rubiaceae | Q156569 |
| RU_318-048 | *Spermacoce articularis* | Q11174485 | Rubiaceae | Q156569 |
| RU_319-083 | *Morinda panamensis* | Q15476545 | Rubiaceae | Q156569 |
| RU_330-064 | *Guettarda combsii* | Q15458581 | Rubiaceae | Q156569 |
| RU_334-054 | *Palicourea galeottiana* | Q39814612 | Rubiaceae | Q156569 |
| RU_338-008 | *Uncaria lanosa* | Q15460581 | Rubiaceae | Q156569 |
| RU_338-037 | *Uncaria gambir* | Q910384 | Rubiaceae | Q156569 |
| RU_345-017 | *Palicourea padifolia* | Q15483171 | Rubiaceae | Q156569 |
| RU_348-074 | *Ophiorrhiza alatiflora* | Q11065092 | Rubiaceae | Q156569 |
| RU_349-088 | *Spermacoce alata* | Q15449863 | Rubiaceae | Q156569 |
| RU_351-031 | *Ophiorrhiza japonica* | Q11086605 | Rubiaceae | Q156569 |

* These plant names were provided by local botanists; however they are not enlisted in databases, and therefore no IDs could be assigned to them.

Table S3 MassQL queries* using product ions and neutral losses

Only queries exhibiting a fscore ≥ 0.95 were tested. The lapidilectine-skeleton query was discarded due to the absence of this skeleton within the GNPS annotated features.

| Skeleton | Query | Query link | N° of features detected | N° of GNPS annotated features |
| --- | --- | --- | --- | --- |
| Ajmalicine spirooxindole | QUERY scaninfo(MS2DATA) WHERE MS2PROD=158.0609:TOLERANCEMZ=0.01 AND MS2PROD=160.0811:TOLERANCEMZ=0.01 AND MS2PROD=108.0818:TOLERANCEMZ=0.01 AND MS2PROD=124.0477:TOLERANCEMZ=0.01 AND MS2NL=237.1365:TOLERANCEMZ=0.01 AND MS2NL=211.1154:TOLERANCEMZ=0.01 AND MS2NL=252.1266:TOLERANCEMZ=0.01 AND MS2NL=237.0967:TOLERANCEMZ=0.01 AND MS2NL=227.1088:TOLERANCEMZ=0.01 | https://gnps.ucsd.edu/ProteoSAFe/status.jsp?task=3e385e5bd89e434e910acca9b9d0f210 | 78 | 16 |
| Corynantheane spirooxindole | QUERY scaninfo(MS2DATA) WHERE MS2PROD=158.0609:TOLERANCEMZ=0.01 AND MS2PROD=160.0811:TOLERANCEMZ=0.01 AND MS2PROD=108.0818:TOLERANCEMZ=0.01 AND MS2PROD=130.0617:TOLERANCEMZ=0.01 AND MS2PROD=129.0561:TOLERANCEMZ=0.01 AND MS2NL=241.1256:TOLERANCEMZ=0.01 AND MS2NL=253.1641:TOLERANCEMZ=0.01 AND MS2NL=243.1449:TOLERANCEMZ=0.01 AND MS2NL=198.1252:TOLERANCEMZ=0.01 AND MS2NL=267.1462:TOLERANCEMZ=0.01 AND MS2NL=213.1226:TOLERANCEMZ=0.01 AND MS2NL=268.1437:TOLERANCEMZ=0.01 | https://gnps.ucsd.ede/ProteoSAFe/index.jsp?task=737b11b4dd904808af7776a1c9a4e9ed | 38 | 7 |
| Combined Ajmalicine spirooxindole/ Corynantheane spirooxindole | QUERY scaninfo(MS2DATA) WHERE MS2PROD=158.0609:TOLERANCEMZ=0.01 AND MS2PROD=160.0811:TOLERANCEMZ=0.01 AND MS2PROD=108.0818:TOLERANCEMZ=0.01 AND MS2PROD=124.0477:TOLERANCEMZ=0.01 AND MS2PROD=132.0474:TOLERANCEMZ=0.01 AND MS2NL=252.1266:TOLERANCEMZ=0.01 | https://gnps.ucsd.edu/ProteoSAFe/index.jsp?task=4cd9ff97d0fd45adb3e2a4e9dd8be4a5 | 108 | 18 |

**It is worth noting that those spectral patterns, disseminated herein as MassQL queries, were retrieved from CID MS/MS spectra acquired in DDA mode at a specific collision energy. Consequently, their interoperability with other MS platforms (e.g., HCD, or other novel fragmentation techniques such as EAD or UVPD) may be limited*.

Table S4 GNPS-annotation based accuracy and recall of the MassQL queries on the 75 plant dataset

| Skeleton | MassQL query accuracy | MassQL query recall |
| --- | --- | --- |
| Ajmalicine spirooxindole | 43.75 % (7/16) | 100 % (7/7) |
| Corynantheane spirooxindole | 42.86 % (3/7) | 75 % (3/4) |
| Ajmalicine spirooxindole or Corynantheane spirooxindole | 55.56 % (10/18) | 90.91 % (10/11) |

Table S5 Information related to the different features being tentatively MassQL tagged as “ajmalicine spirooxindole” in the molecular network obtained from the 75 plant extracts dataset. “Biosource” column indicates the plant species where the signal intensity was the most important in all the dataset. In case an experimental MS/MS annotation was proposed by the GNPS, its name, SMILES strings and corresponding MIA skeleton are shown.

| Cluster index | Biosource | GNPS annotation | SMILES of the GNPS annotated ion | Skeleton type of the GNPS annotated ion |
| --- | --- | --- | --- | --- |
| 2057 | *Uncaria scandens* | Uncarine F | C[C@H]1[C@@H]2CN3CC[C@]4([C@H]3C[C@@H]2C(=CO1)C(=O)OC)C5=CC=CC=C5NC4=O | Ajmalicine spirooxindole |
| 2322 | *Mitragyna rotundifolia* | Corynoxeine | O=C(OC)C(=COC)C1CC2N(CCC32C(=O)NC=4C=CC=CC43)CC1C=C | Corynantheane spirooxindole |
| 2273 | *Uncaria scandens* |  |  |  |
| 1809 | *Rauvolfia littoralis* | Rauwolscine | COC(=O)C1C(O)CCC2CN3CCC4=C(NC5=CC=CC=C45)C3CC12 | Yohimbinoid |
| 2754 | *Mitragyna rotundifolia* |  |  |  |
| 2220 | *Mitragyna rotundifolia* |  |  |  |
| 3114 | *Mitragyna rotundifolia* |  |  |  |
| 3210 | *Mitragyna rotundifolia* |  |  |  |
| 2784 | *Mitragyna rotundifolia* |  |  |  |
| 2269 | *Mitragyna hirsuta* | Isomitraphylline | C[C@H]1[C@H]2CN3CC[C@@]4([C@@H]3C[C@@H]2C(=CO1)C(=O)OC)C5=CC=CC=C5NC4=O | Ajmalicine spirooxindole |
| 2752 | *Mitragyna hirsuta* | Isocorynoxine | O=C1NC2=CC=CC=C2[C@@]13CN4[C@](C[C@H](/C(C(OC)=O)=C\\OC)[C@H](CC)C4)([H])C3 | Corynantheane spirooxindole |
| 2653 | *Mitragyna hirsuta* |  |  |  |
| 1595 | *Tabernaemontana bovina* | Tabernaemontanine | CN1C(C2([H])C(OC)=O)CC3=C(C(CC2[C@H](CC)C1)=O)NC4=C3C=CC=C4 | Vobasine |
| 1604 | *Stemmadenia pubescens* |  |  |  |
| 1119 | *Stemmadenia pubescens* |  |  |  |
| 2400 | *Mitragyna rotundifolia* |  |  |  |
| 3660 | *Stemmadenia pubescens* |  |  |  |
| 3470 | *Mitragyna hirsuta* |  |  |  |
| 2172 | *Rauvolfia littoralis* |  |  |  |
| 1521 | *Rauvolfia tetraphylla* |  |  |  |
| 2469 | *Rauvolfia tetraphylla* |  |  |  |
| 1395 | *Strychnos thorelii* |  |  |  |
| 2279 | *Rauvolfia tetraphylla* | Carapanaubine | COC(C([C@]1(C2)[H])=CO[C@H]([C@@]1(CN3CCC4(C(NC(C=C5OC)=C4C=C5OC)=O)[C@]23[H])[H])C)=O | Ajmalicine spirooxindole |
| 702 | *Rauvolfia tetraphylla* |  |  |  |
| 2237 | *Rauvolfia littoralis* |  |  |  |
| 2425 | *Mitragyna rotundifolia* |  |  |  |
| 2005 | *Rauvolfia littoralis* | Carapanaubine | COC(C([C@]1(C2)[H])=CO[C@H]([C@@]1(CN3CCC4(C(NC(C=C5OC)=C4C=C5OC)=O)[C@]23[H])[H])C)=O | Ajmalicine spirooxindole |
| 1654 | *Psychotria peducunlaris* var. *peduncularis* |  |  |  |
| 3456 | *Stemmadenia pubescens* |  |  |  |
| 3859 | *Uncaria scandens* |  |  |  |
| 1877 | *Rauvolfia littoralis* |  |  |  |
| 2956 | *Mitragyna rotundifolia* |  |  |  |
| 2928 | *Mitragyna hirsuta* |  |  |  |
| 2874 | *Uncaria scandens* | Speciophylline | COC(=O)C1=CO[C@@H](C)[C@@H]2CN3CC[C@]4([C@H]3C[C@H]12)C(O)=NC1=CC=CC=C41 | Ajmalicine spirooxindole |
| 3564 | *Tabernaemontana granatum* |  |  |  |
| 1867 | *Uncaria scandens* |  |  |  |
| 695 | *Rauvolfia micrantha* |  |  |  |
| 2289 | *Tabernaemontana bovina* | Ervatamine | CN(C1)C[C@@H](CC)[C@]([C@]1(C(OC)=O)C2)([H])CC(C3=C2C4=CC=CC=C4N3)=O | Ervatamia |
| 2725 | *Rauvolfia littoralis* |  |  |  |
| 3517 | *Mitragyna hirsuta* | Reserpinine | O=C(OC)C1=COC(C)C2CN3CCC=4C=5C=CC(OC)=CC5NC4C3CC12 | Ajmalicine |
| 1706 | *Tabernaemontana bovina* |  |  |  |
| 2285 | *Rauvolfia tetraphylla* |  |  |  |
| 1818 | *Tabernaemontana bovina* | 7α-voacangine hydroxyindolenine | CC[C@@H]1C[C@@H]2CN3CCC4(O)C5=C(C=CC(OC)=C5)N=C4C(C2)(C13)C(=O)OC | Iboga |
| 1427 | *Rauvolfia micrantha* |  |  |  |
| 2618 | *Timonius arboreus* |  |  |  |
| 3285 | *Mitragyna hirsuta* | Corynantheidine | [H][C@](N1CC2)(C[C@@](/C(C(OC)=O)=C\\OC)([H])[C@H](CC)C1)C3=C2C4=CC=CC=C4N3 | Corynantheane |
| 1737 | *Mitragyna rotundifolia* |  |  |  |
| 3234 | *Stemmadenia pubescens* |  |  |  |
| 3241 | *Uncaria scandens* |  |  |  |
| 1011 | *Strychnos thorelii* |  |  |  |
| 2264 | *Mitragyna rotundifolia* | Methyl-(16*Z*)-9-hydroxy-16-(methoxymethylene)-2-oxocoryxan-17-oate | CCC1CN2CCC3(C2CC1\\C(=C\\OC)C(=O)OC)C(=O)NC4=C3C(=CC=C4)O | Corynantheane spirooxindole |
| 2680 | *Mitragyna rotundifolia* |  |  |  |
| 773 | *Rauvolfia tetraphylla* |  |  |  |
| 1097 | *Stemmadenia pubescens* |  |  |  |
| 1326 | *Rauvolfia littoralis* |  |  |  |
| 1583 | *Rauvolfia littoralis* |  |  |  |
| 2628 | *Mitragyna rotundifolia* |  |  |  |
| 1798 | *Stemmadenia pubescens* |  |  |  |
| 817 | *Rauvolfia micrantha* |  |  |  |
| 2522 | *Rauvolfia littoralis* |  |  |  |
| 1649 | *Tabernaemontana granatum* |  |  |  |
| 3647 | *Isertia laevis* |  |  |  |
| 3188 | *Rauvolfia littoralis* |  |  |  |
| 3081 | *Stemmadenia pubescens* |  |  |  |
| 1189 | *Rauvolfia micrantha* |  |  |  |
| 3990 | *Rauvolfia littoralis* |  |  |  |
| 763 | *Strychnos thorelii* |  |  |  |
| 2886 | *Rauvolfia littoralis* |  |  |  |
| 2695 | *Mitragyna rotundifolia* |  |  |  |
| 3040 | *Tabernaemontana granatum* |  |  |  |
| 3005 | *Uncaria scandens* | Speciophylline | COC(=O)C1=CO[C@@H](C)[C@@H]2CN3CC[C@]4([C@H]3C[C@H]12)C(O)=NC1=CC=CC=C41 | Ajmalicine spirooxindole |
| 1291 | *Rauvolfia micrantha* |  |  |  |
| 1414 | *Stemmadenia pubescens* |  |  |  |
| 2810 | *Rauvolfia littoralis* |  |  |  |
| 2510 | *Mitragyna rotundifolia* |  |  |  |
| 3507 | *Mitragyna hirsuta* |  |  |  |
| 1513 | *Uncaria scandens* |  |  |  |
| 1882 | *Uncaria scandens* | Formosanine | COC(=O)C1=CO[C@H](C)[C@H]2CN3CC[C@@]4([C@@H]3C[C@H]12)C(O)=NC1=CC=CC=C41 | Ajmalicine spirooxindole |

Table S6 Most common plant sources genera for ions with the highest signal intensity for features tentatively identified by MassQL as an ajmalicine spirooxindole. It can be noted that all these plant genera are established MIA producers.

| Genus |  |
| --- | --- |
| *Rauvolfia* | 30.8 % (24/78) |
| *Mitragyna* | 29.4 % (23/78) |
| *Uncaria* | 11.5 % (9/78) |
| *Stemmadenia* | 11.5 % (9/78) |
| *Tabernaemontana* | 9.0 % (7/78) |
| *Strychnos* | 3.8 % (3/78) |
| *Psychotria* | 1.3 % (1/78) |
| *Isertia* | 1.3 % (1/78) |
| *Timonius* | 1.3 % (1/78) |

Table S7 Information related to the different features being tentatively MassQL tagged as “corynantheane spirooxindole” in the molecular network obtained from the 75 plant extracts dataset. “Biosource” column indicates the plant species where the signal intensity was the most important in all the dataset. In case an experimental MS/MS annotation was proposed by the GNPS, its name, SMILES strings and corresponding MIA skeleton are shown.

| Cluster index | Biosource | GNPS annotation | SMILES of the GNPS annotated ion | Skeleton type |
| --- | --- | --- | --- | --- |
| 2322 | *Mitragyna rotundifolia* | Corynoxeine | O=C(OC)C(=COC)C1CC2N(CCC32C(=O)NC=4C=CC=CC43)CC1C=C | Corynantheane spirooxindole |
| 2754 | *Mitragyna rotundifolia* |  |  |  |
| 3114 | *Mitragyna rotundifolia* |  |  |  |
| 3210 | *Mitragyna rotundifolia* |  |  |  |
| 2784 | *Mitragyna rotundifolia* |  |  |  |
| 2752 | *Mitragyna hirsuta* | Isocorynoxine | O=C1NC2=CC=CC=C2[C@@]13CN4[C@](C[C@H](/C(C(OC)=O)=C\\OC)[C@H](CC)C4)([H])C3 | Corynantheane spirooxindole |
| 1604 | *Stemmadenia pubescens* |  |  |  |
| 2400 | *Mitragyna rotundifolia* |  |  |  |
| 2882 | *Mitragyna rotundifolia* |  |  |  |
| 3660 | *Stemmadenia pubescens* |  |  |  |
| 1386 | *Rauvolfia littoralis* |  |  |  |
| 1521 | *Rauvolfia tetraphylla* |  |  |  |
| 2279 | *Rauvolfia tetraphylla* | Carapanaubine | COC(C([C@]1(C2)[H])=CO[C@H]([C@@]1(CN3CCC4(C(NC(C=C5OC)=C4C=C5OC)=O)[C@]23[H])[H])C)=O | Ajmalicine spirooxindole |
| 2005 | *Rauvolfia littoralis* | Carapanaubine | COC(C([C@]1(C2)[H])=CO[C@H]([C@@]1(CN3CCC4(C(NC(C=C5OC)=C4C=C5OC)=O)[C@]23[H])[H])C)=O | Ajmalicine spirooxindole |
| 1654 | *Psychotria peducunlaris* var. *peduncularis* |  |  |  |
| 3456 | *Stemmadenia pubescens* |  |  |  |
| 2956 | *Mitragyna rotundifolia* |  |  |  |
| 2928 | *Mitragyna hirsuta* |  |  |  |
| 1923 | *Psychotria peducunlaris var. peduncularis* |  |  |  |
| 3564 | *Tabernaemontana granatum* |  |  |  |
| 1867 | *Uncaria scandens* |  |  |  |
| 3668 | *Mitragyna hirsuta* |  |  |  |
| 2725 | *Rauvolfia littoralis* |  |  |  |
| 734 | *Rauvolfia tetraphylla* |  |  |  |
| 1706 | *Tabernaemontana bovina* |  |  |  |
| 1818 | *Tabernaemontana bovina* | 7α-voacangine hydroxyindolenine | CC[C@@H]1C[C@@H]2CN3CCC4(O)C5=C(C=CC(OC)=C5)N=C4C(C2)(C13)C(=O)OC | Iboga |
| 1125 | *Rauvolfia micrantha* |  |  |  |
| 2264 | *Mitragyna rotundifolia* | Methyl-(16*Z*)-9-hydroxy-16-(methoxymethylene)-2-oxocoryxan-17-oate | CCC1CN2CCC3(C2CC1\\C(=C\\OC)C(=O)OC)C(=O)NC4=C3C(=CC=C4)O | Corynantheane spirooxindole |
| 2628 | *Mitragyna rotundifolia* |  |  |  |
| 2030 | *Tabernaemontana granatum* |  |  |  |
| 1798 | *Stemmadenia pubescens* |  |  |  |
| 2522 | *Rauvolfia littoralis* |  |  |  |
| 1649 | *Tabernaemontana granatum* |  |  |  |
| 3081 | *Stemmadenia pubescens* |  |  |  |
| 2256 | *Stemmadenia pubescens* |  |  |  |
| 2555 | *Rauvolfia littoralis* | Isoreserpiline | C[C@H]1[C@@H]2CN3CCC4=C([C@@H]3C[C@@H]2C(=CO1)C(=O)OC)NC5=CC(=C(C=C45)OC)OC | Ajmalicine |
| 3306 | *Mitragyna rotundifolia* |  |  |  |
| 3796 | *Mitragyna hirsuta* |  |  |  |

Table S8 Most common plant sources genera for ions with the highest signal intensity for features tentatively identified by MassQL as a corynantheane spirooxindole. It can be noted that all these plant genera are established MIA producers.

| Genus | Percentage |
| --- | --- |
| *Mitragyna* | 39.5 % (15/38) |
| *Rauvolfia* | 23.7 % (9/38) |
| *Stemmadenia* | 15.8 % (6/38) |
| *Tabernaemontana* | 13.2 % (5/38) |
| *Psychotria* | 5.3 % (2/38) |
| *Uncaria* | 2.6 % (1/38) |

Table S9 Information related to the different features being tentatively MassQL tagged as “corynantheane spirooxindole or ajmalicine spirooxindole” in the molecular network obtained from the 75 plant extracts dataset. “Biosource” column indicates the plant species where the signal intensity was the most important in all the dataset. In case an experimental MS/MS annotation was proposed by the GNPS, its name, SMILES strings and corresponding MIA skeleton are shown.

| Cluster index | Biosource | GNPS annotation | SMILES of the GNPS annotated ion | Skeleton type |
| --- | --- | --- | --- | --- |
| 2057 | *Uncaria scandens* | Uncarine F | C[C@H]1[C@@H]2CN3CC[C@]4([C@H]3C[C@@H]2C(=CO1)C(=O)OC)C5=CC=CC=C5NC4=O | Ajmalicine spirooxindole |
| 2322 | *Mitragyna rotundifolia* | Corynoxeine | O=C(OC)C(=COC)C1CC2N(CCC32C(=O)NC=4C=CC=CC43)CC1C=C | Corynantheane spirooxindole |
| 2273 | *Uncaria scandens* |  |  |  |
| 1809 | *Rauvolfia littoralis* | Rauwolscine | COC(=O)C1C(O)CCC2CN3CCC4=C(NC5=CC=CC=C45)C3CC12 | Yohimbinoid |
| 2754 | *Mitragyna rotundifolia* |  |  |  |
| 2220 | *Mitragyna rotundifolia* |  |  |  |
| 3114 | *Mitragyna rotundifolia* |  |  |  |
| 3210 | *Mitragyna rotundifolia* |  |  |  |
| 2859 | *Uncaria scandens* |  |  |  |
| 2784 | *Mitragyna rotundifolia* |  |  |  |
| 2269 | *Mitragyna hirsuta* | Isomitraphylline | C[C@H]1[C@H]2CN3CC[C@@]4([C@@H]3C[C@@H]2C(=CO1)C(=O)OC)C5=CC=CC=C5NC4=O | Ajmalicine spirooxindole |
| 2752 | *Mitragyna hirsuta* | Isocorynoxine | O=C1NC2=CC=CC=C2[C@@]13CN4[C@](C[C@H](/C(C(OC)=O)=C\\OC)[C@H](CC)C4)([H])C3 | Corynantheane spirooxindole |
| 2653 | *Mitragyna hirsuta* |  |  |  |
| 1595 | *Tabernaemontana bovina* | Tabernaemontanine | CN1C(C2([H])C(OC)=O)CC3=C(C(CC2[C@H](CC)C1)=O)NC4=C3C=CC=C4 | Vobasine |
| 1604 | *Stemmadenia pubescens* |  |  |  |
| 1119 | *Stemmadenia pubescens* |  |  |  |
| 2400 | *Mitragyna rotundifolia* |  |  |  |
| 2882 | *Mitragyna rotundifolia* |  |  |  |
| 3660 | *Stemmadenia pubescens* |  |  |  |
| 3470 | *Mitragyna hirsuta* |  |  |  |
| 2172 | *Rauvolfia littoralis* |  |  |  |
| 1741 | *Uncaria scandens* | Pumiloside | OC[C@H]1O[C@@H](O[C@@H]2O\\C=C3/[C@@H](C[C@@H]4N(CC5=C(O)C6=CC=CC=C6N=C45)C3=O)[C@H]2C=C)[C@H](O)[C@@H](O)[C@@H]1O | Camptothecin |
| 1521 | *Rauvolfia tetraphylla* |  |  |  |
| 2469 | *Rauvolfia tetraphylla* |  |  |  |
| 1395 | *Strychnos thorelii* |  |  |  |
| 2279 | *Rauvolfia tetraphylla* | Carapanaubine | COC(C([C@]1(C2)[H])=CO[C@H]([C@@]1(CN3CCC4(C(NC(C=C5OC)=C4C=C5OC)=O)[C@]23[H])[H])C)=O | Ajmalicine spirooxindole |
| 702 | *Rauvolfia tetraphylla* |  |  |  |
| 2237 | *Rauvolfia littoralis* |  |  |  |
| 3602 | *Mitragyna hirsuta* |  |  |  |
| 2425 | *Mitragyna rotundifolia* | Carapanaubine | COC(C([C@]1(C2)[H])=CO[C@H]([C@@]1(CN3CCC4(C(NC(C=C5OC)=C4C=C5OC)=O)[C@]23[H])[H])C)=O | Ajmalicine spirooxindole |
| 2005 | *Rauvolfia littoralis* | Carapanaubine | COC(C([C@]1(C2)[H])=CO[C@H]([C@@]1(CN3CCC4(C(NC(C=C5OC)=C4C=C5OC)=O)[C@]23[H])[H])C)=O | Ajmalicine spirooxindole |
| 1654 | *Psychotria peducunlaris* var. *peduncularis* |  |  |  |
| 3456 | *Stemmadenia pubescens* |  |  |  |
| 3859 | *Uncaria scandens* |  |  |  |
| 1877 | *Rauvolfia littoralis* |  |  |  |
| 2956 | *Mitragyna rotundifolia* |  |  |  |
| 2928 | *Mitragyna hirsuta* |  |  |  |
| 2874 | *Uncaria scandens* | Speciophylline | COC(=O)C1=CO[C@@H](C)[C@@H]2CN3CC[C@]4([C@H]3C[C@H]12)C(O)=NC1=CC=CC=C41 | Ajmalicine spirooxindole |
| 3564 | *Tabernaemontana granatum* |  |  |  |
| 1867 | *Uncaria scandens* |  |  |  |
| 695 | *Rauvolfia micrantha* |  |  |  |
| 3668 | *Mitragyna hirsuta* |  |  |  |
| 2289 | *Tabernaemontana bovina* | Ervatamine | CN(C1)C[C@@H](CC)[C@]([C@]1(C(OC)=O)C2)([H])CC(C3=C2C4=CC=CC=C4N3)=O | Ervatamia |
| 2725 | *Rauvolfia littoralis* |  |  |  |
| 3517 | *Mitragyna hirsuta* |  |  |  |
| 1706 | *Tabernaemontana bovina* |  |  |  |
| 2285 | *Rauvolfia tetraphylla* |  |  |  |
| 1818 | *Tabernaemontana bovina* | 7α-voacangine hydroxyindolenine | CC[C@@H]1C[C@@H]2CN3CCC4(O)C5=C(C=CC(OC)=C5)N=C4C(C2)(C13)C(=O)OC | Iboga |
| 2618 | *Timonius arboreus* |  |  |  |
| 3285 | *Mitragyna hirsuta* | Corynantheidine | [H][C@](N1CC2)(C[C@@](/C(C(OC)=O)=C\\OC)([H])[C@H](CC)C1)C3=C2C4=CC=CC=C4N3 | Corynantheane |
| 1737 | *Mitragyna rotundifolia* |  |  |  |
| 3234 | *Stemmadenia pubescens* |  |  |  |
| 3241 | *Uncaria scandens* |  |  |  |
| 1011 | *Strychnos thorelii* |  |  |  |
| 2264 | *Mitragyna rotundifolia* | Methyl-(16*Z*)-9-hydroxy-16-(methoxymethylene)-2-oxocoryxan-17-oate | CCC1CN2CCC3(C2CC1\\C(=C\\OC)C(=O)OC)C(=O)NC4=C3C(=CC=C4)O | Corynantheane spirooxindole |
| 2680 | *Mitragyna rotundifolia* |  |  |  |
| 773 | *Rauvolfia tetraphylla* |  |  |  |
| 1097 | *Stemmadenia pubescens* |  |  |  |
| 1326 | *Rauvolfia littoralis* |  |  |  |
| 1583 | *Rauvolfia littoralis* |  |  |  |
| 2628 | *Mitragyna rotundifolia* |  |  |  |
| 2030 | *Tabernaemontana granatum* |  |  |  |
| 1798 | *Stemmadenia pubescens* |  |  |  |
| 817 | *Rauvolfia micrantha* |  |  |  |
| 2522 | *Rauvolfia littoralis* |  |  |  |
| 1649 | *Tabernaemontana granatum* |  |  |  |
| 3647 | *Isertia laevis* |  |  |  |
| 3188 | *Rauvolfia littoralis* |  |  |  |
| 3081 | *Stemmadenia pubescens* |  |  |  |
| 1189 | *Rauvolfia micrantha* |  |  |  |
| 3990 | *Rauvolfia littoralis* |  |  |  |
| 2800 | *Uncaria scandens* |  |  |  |
| 2555 | *Rauvolfia littoralis* | Isoreserpiline | C[C@H]1[C@@H]2CN3CCC4=C([C@@H]3C[C@@H]2C(=CO1)C(=O)OC)NC5=CC(=C(C=C45)OC)OC | Ajmalicine |
| 3865 | *Uncaria scandens* |  |  |  |
| 763 | *Strychnos thorelii* |  |  |  |
| 452 | *Rauvolfia littoralis* |  |  |  |
| 1922 | *Uncaria scandens* |  |  |  |
| 2886 | *Rauvolfia littoralis* |  |  |  |
| 2363 | *Rauvolfia littoralis* |  |  |  |
| 2695 | *Mitragyna rotundifolia* |  |  |  |
| 3040 | *Tabernaemontana granatum* |  |  |  |
| 1478 | *Strychnos ovata* |  |  |  |
| 3005 | *Uncaria scandens* | Speciophylline | COC(=O)C1=CO[C@@H](C)[C@@H]2CN3CC[C@]4([C@H]3C[C@H]12)C(O)=NC1=CC=CC=C41 | Ajmalicine spirooxindole |
| 1291 | *Rauvolfia micrantha* |  |  |  |
| 3683 | *Melodinus cochinchinensis* |  |  |  |
| 3097 | *Isertia laevis* |  |  |  |
| 1682 | *Mitragyna rotundifolia* |  |  |  |
| 3892 | *Mitragyna rotundifolia* |  |  |  |
| 954 | *Rauvolfia micrantha* |  |  |  |
| 2229 | *Mitragyna rotundifolia* |  |  |  |
| 3266 | *Mitragyna rotundifolia* |  |  |  |
| 2915 | *Rauvolfia littoralis* |  |  |  |
| 3880 | *Kopsia singapurensis* |  |  |  |
| 1530 | *Rauvolfia littoralis* |  |  |  |
| 1688 | *Uncaria scandens* |  |  |  |
| 2810 | *Rauvolfia littoralis* |  |  |  |
| 1118 | *Tabernaemontana granatum* |  |  |  |
| 2510 | *Mitragyna rotundifolia* |  |  |  |
| 2583 | *Uncaria scandens* |  |  |  |
| 3507 | *Mitragyna hirsuta* |  |  |  |
| 3258 | *Mitragyna rotundifolia* |  |  |  |
| 2027 | *Rauvolfia littoralis* |  |  |  |
| 1670 | *Psychotria peducunlaris* var. *peduncularis* |  |  |  |
| 1513 | *Uncaria scandens* |  |  |  |
| 651 | *Uncaria scandens* |  |  |  |
| 2036 | *Uncaria scandens* |  |  |  |
| 1882 | *Uncaria scandens* | Formosanine | COC(=O)C1=CO[C@H](C)[C@H]2CN3CC[C@@]4([C@@H]3C[C@H]12)C(O)=NC1=CC=CC=C41 | Ajmalicine spirooxindole |
| 3931 | *Mitragyna rotundifolia* |  |  |  |

Table S10 Plant sources genera for ions with the highest signal intensity for features tentatively identified by MassQL as “corynantheane spirooxindole or ajmalicine spirooxindole”. It can be noted that all these plant genera are established MIA producers.

| Genus |  |
| --- | --- |
| *Mitragyna* | 29.6 % (32/108) |
| *Rauvolfia* | 27.8 % (30/108) |
| *Uncaria* | 16.7 % (18/108) |
| *Tabernaemontana* | 8.3 % (9/108) |
| *Stemmadenia* | 7.4 % (8/108) |
| *Strychnos* | 3.7 % (4/108) |
| *Psychotria* | 1.9 % (2/108) |
| *Isertia* | 1.9 % (2/108) |
| *Kopsia* | 0.9 % (1/108) |
| *Melodinus* | 0.9 % (1/108) |
| *Timonius* | 0.9 % (1/108) |


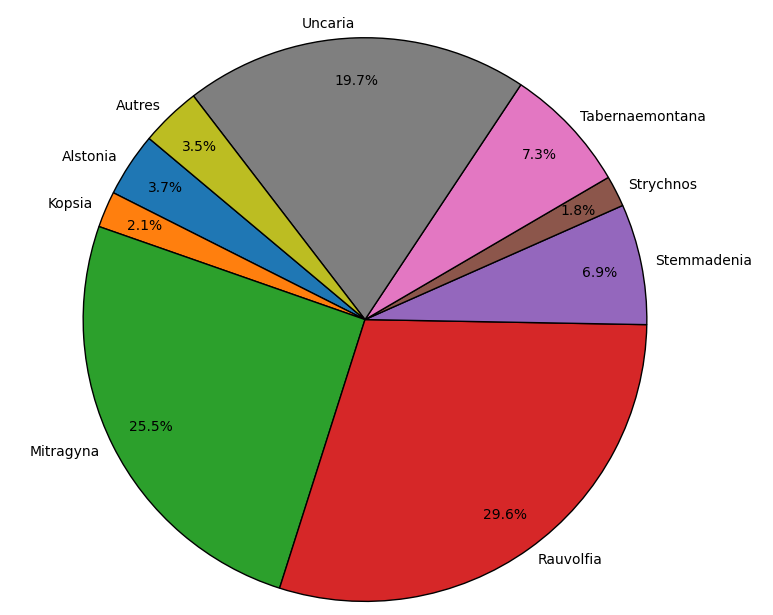


Fig. S15 Genus repartition of the ions annotated as ajmalicine spirooxindoles by the MassQL query in the 75 plant extracts dataset


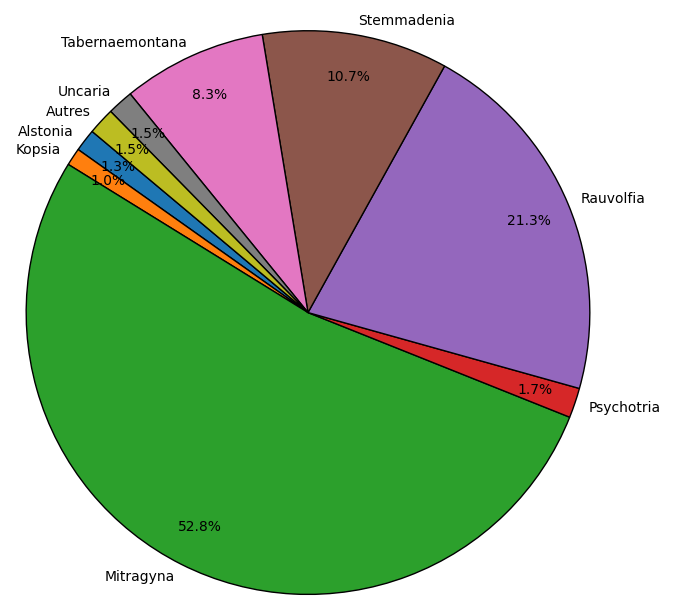


Fig. S16 Genus repartition of the ions annotated as corynantheane spirooxindoles by the MassQL query in the 75 plant extracts dataset


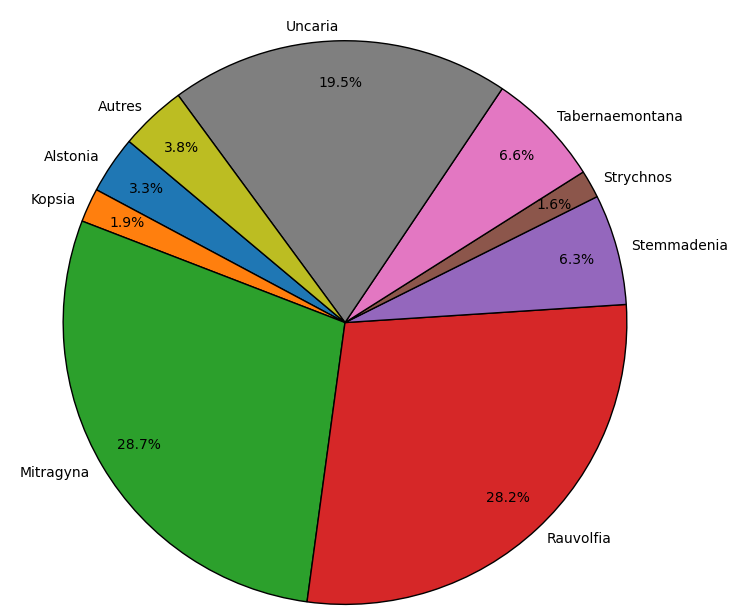
Fig. S17 Genus repartition of the ions annotated as “ajmalicine spirooxindole or corynantheane spirooxindole” by the MassQL query in the 75 plant extracts dataset


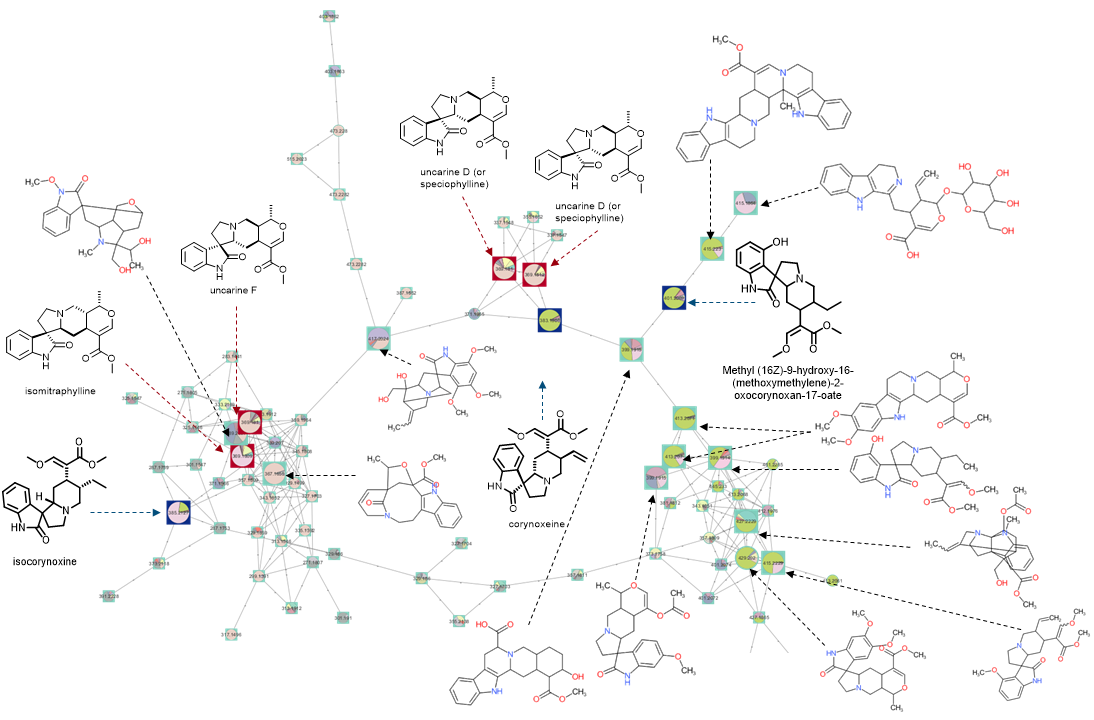
Fig. S18 Spirooxindole-type MIA containing cluster of the molecular network obtained from the 75 plant extracts revealing nodes tentatively annotated by the GNPS, the MassQL query and/or TIMA. Emphasized nodes refer to MassQL spirooxindole-tagged ions. TIMA annotated nodes are shown as squares. Red and blue squares had been tentatively annotated against the GNPS spectral repositories as ajmalicine spirooxindole or corynantheane spirooxindole, respectively.


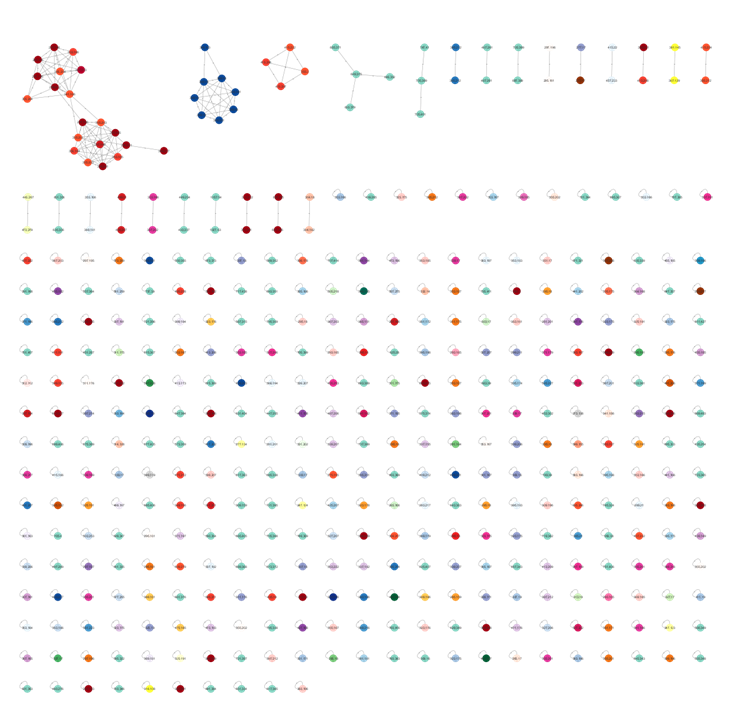


Fig. S19 Global MIADB network with skeleton-annotated features (job link: <https://gnps.ucsd.edu/ProteoSAFe/status.jsp?task=fca55f3aa80d421fb0d090099234983d>)


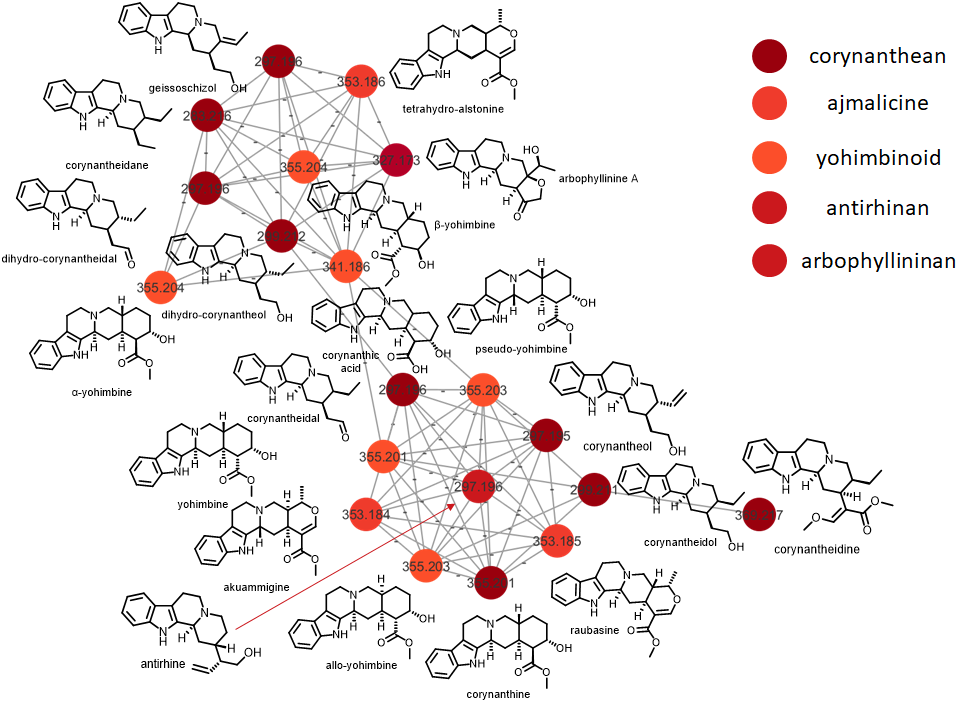


Fig. S20 Global MIADB network: zoom on the indoloquinolizidine-containing MIA cluster


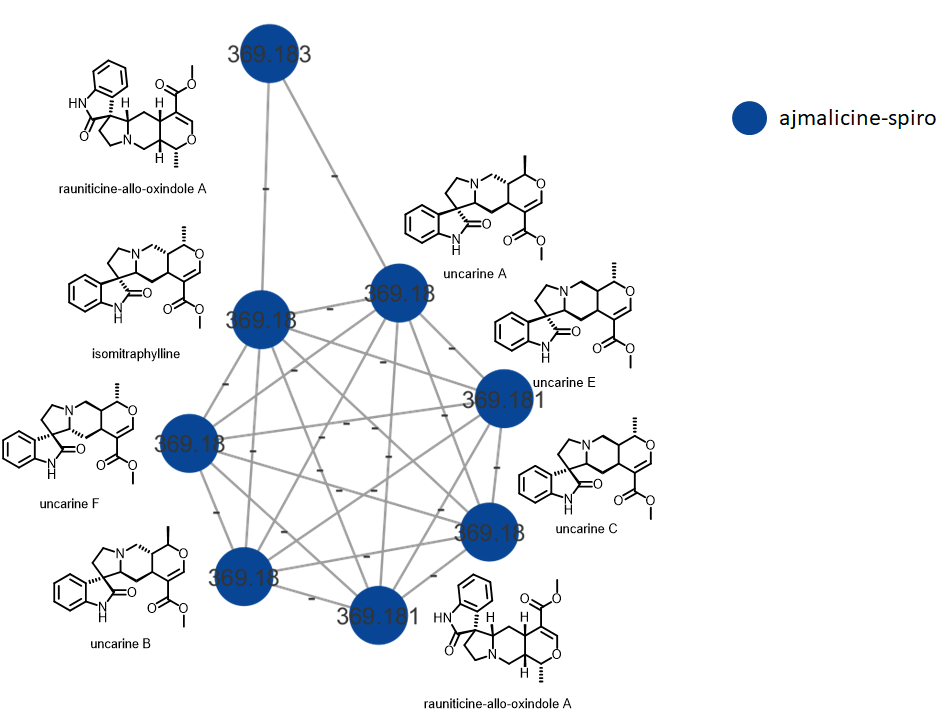
Fig. S21 Global MIADB network : zoom on the ajmalicine spirooxindole cluster
